# Cation-directed assembly and sequential functionalization enable superprotonic polyanion–organic frameworks for high-power fuel cells

**DOI:** 10.1038/s41557-026-02169-8

**Published:** 2026-06-01

**Authors:** Qixin Zhao, Bo Li, Dhruv Menon, Chunmei Zhu, Mohammad Reza Alizadeh Kiapi, Xu Chen, De-Liang Long, Jianfeng Liu, Zhou Xiao, Dongsheng Yang, David Fairen-Jimenez, Hong-Ying Zang, Weimin Xuan

**Affiliations:** 1 State Key Laboratory of Advanced Fiber Materials, College of Chemistry and Chemical Engineering, Donghua University, Shanghai, P. R. China; 2 Key Lab of Polyoxometalate and Reticular Material Chemistry of Ministry of Education, Northeast Normal University, Changchun, P. R. China; 3 State Key Laboratory of Organometallic Chemistry, Shanghai Institute of Organic Chemistry, University of Chinese Academy of Sciences, Chinese Academy of Sciences, Shanghai, P. R. China; 4 The Adsorption & Advanced Materials Laboratory, Department of Chemical Engineering & Biotechnology, University of Cambridge, Cambridge, UK; 5 School of Chemistry, The University of Glasgow, Glasgow, UK; 6 BSD Instrument Technology (Beijing) Co., Ltd, Beijing, P. R. China

**Keywords:** Self-assembly, Energy, Fuel cells, Nanoscale materials

## Abstract

Proton-exchange membranes are critical for high-performance fuel cells, yet simultaneous enhancement of their power density and operational stability remains challenging. Here we introduce a supramolecular engineering approach for constructing polyoxometalate–organic frameworks through precise integration of trigonal-shaped cationic tectons and polyoxometalate anions. Size-matched anchoring of polyoxometalates via directional C–H···anion hydrogen bonds—enabled by shape-persistent tectons—creates ordered one-dimensional channels lined with imidazolium groups. Post-synthetic modification with sulfonate groups enabled our frameworks to achieve high proton conductivity, with proton transport governed by channel-selective hydration, percolated hydrogen-bond networks and host–guest interactions under confinement. The porous ionic structure and flexible hybrid nature of these frameworks provide exceptional solution processability and compatibility with polymer matrices. Integration into Nafion resins dramatically enhances the proton conductivity and chemical stability of the resulting hybrid membranes, as well as enhancing the peak power density and current density over commercial Nafion. This strategy provides a promising blueprint for developing fillers in proton-exchange membrane design to advance fuel cells towards decarbonization targets.

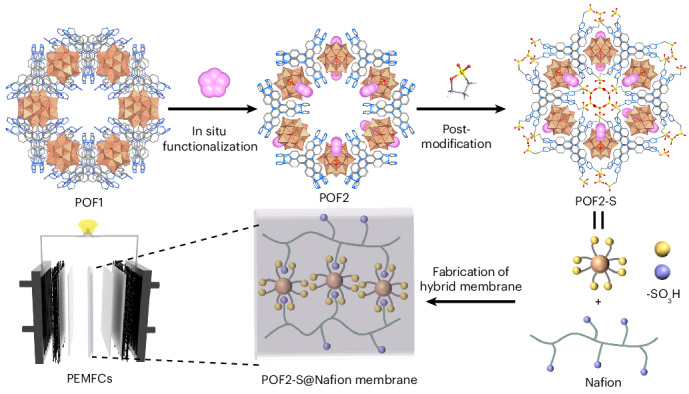

## Abstract (web summary)

Achieving simultaneous gains in power density and operational stability in proton-exchange membranes remains a formidable challenge. Now it has been shown that cation-directed assembly and sequential functionalization enable superprotonic polyanion–organic frameworks to serve as multifunctional fillers, delivering both high performance and durability in hybrid membranes for next-generation fuel cells.

## Main

The transition from fossil fuels to renewable energy is crucial for achieving global net-zero targets. In a hydrogen-based energy economy, hydrogen serves as both an energy carrier and storage medium to buffer fluctuations in renewable supply^1^. Green hydrogen, produced from renewable sources such as photocatalytic water splitting^2^, minimizes greenhouse gas emissions and supports decarbonization goals^1,3^. However, efficient conversion of hydrogen’s chemical energy into electricity remains a key challenge. Proton-exchange membrane fuel cells (PEMFCs) offer a promising solution, yet their core component—the proton-exchange membrane (PEM)—limits current and power density^1^. These constraints arise from the intrinsic drawbacks of commercial PEMs such as Nafion and sulfonated poly(ether ether ketone)^4,5^, whose complex microphase-separated morphologies impose a selectivity–conductivity trade-off and obscure proton-transport mechanisms, restricting rational design^3–5^. With projected PEM improvements of ~20%^1^, fundamentally new material classes are probably required to achieve step changes in performance.

Crystalline porous materials are a promising alternative due to their structural tunabilities and well-defined architectures. Unlike traditional systems such as zeolites, metal–organic frameworks (MOFs) and covalent organic frameworks (COFs), which rely on strong directional covalent or coordination bonds^6–9^, emerging materials based on weak intermolecular interactions have demonstrated notable potential. Examples include crystalline porous organic salts (CPOSs) and charge-assisted hydrogen-bonded organic frameworks (HOFs), assembled through ionic interactions and hydrogen bonding^10–12^. These frameworks assemble directional acid and base tectons via N–H···anion hydrogen bonds (Supplementary Fig. 1)^13–15^, yielding high charge densities within their pores and enabling spatially oriented synthesis analogous to reticular chemistry^16,17^. This design flexibility makes them promising PEM candidates, as demonstrated by the porous, highly proton-conductive isoreticular ammonium halides reported by Cooper and others^18,19^.

**Fig. 1:**
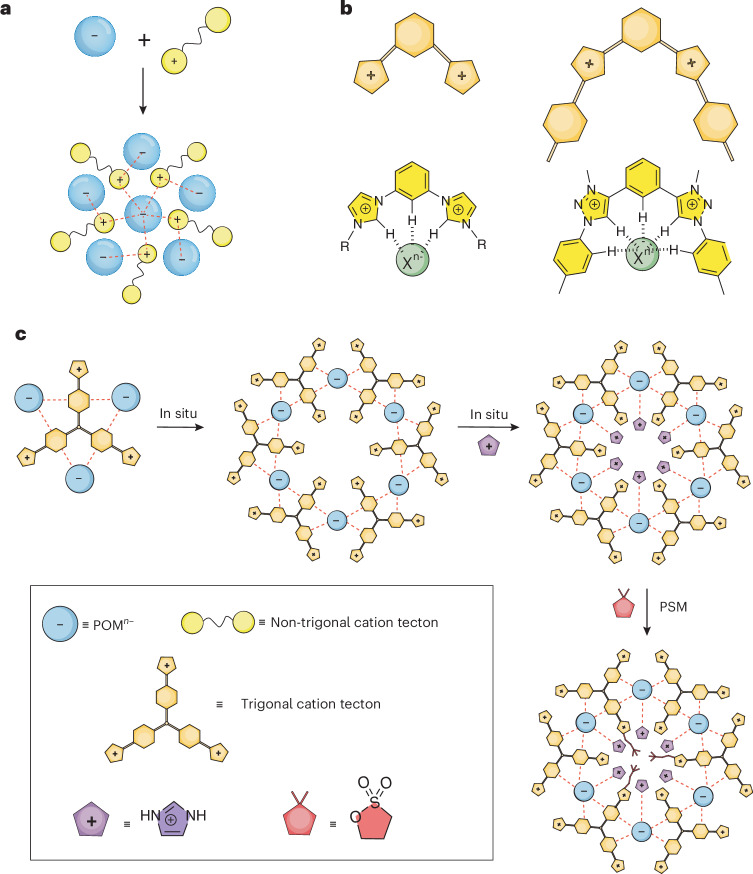
Schematic synthesis and functionalization of porous polyoxometalate–organic frameworks. **a**, Formation of nonporous frameworks from POMs and size-mismatched cation tectons. **b**, Representative V-shaped cationic C–H anion acceptors. **c**, The current work. Synthesis of porous POFs using trigonal cationic tectons bearing V-shaped pockets via ionic interactions and multiple weak hydrogen bonds, followed by sequential functionalization through in situ imidazolium incorporation and post-synthetic sulfonation.

Building on MOF chemistry, where structural diversity and stability arise from extending single metal nodes into high-valency clusters^20,21^, similar strategies could extend to CPOSs and HOFs. However, replacing simple acid radicals or halides with rigid polyanions such as oligomeric phosphates, silicates, borates and polynuclear metal–oxo clusters remains challenging^22–24^. Among these, polyoxometalates (POMs)—nanoscale polyanionic metal–oxo clusters comprising elements such as Mo, W, V, Nb and Ta—are promising candidates due to their rigidity and multiple hydrogen-bonding sites^25,26^. Nevertheless, attempts to assemble POMs with cationic tectons such as ammonium, amidinium or guanidinium have yielded nonporous structures^27–29^. This arises from the size mismatch between cationic tectons and POMs, leading to nondirectional electrostatic interactions and dense packing (Fig. 1a). Overcoming this requires cationic tectons rationally designed to direct assembly and preserve porosity.

In this Article, to address these challenges, we propose a design strategy using shape-persistent, trigonal cationic tectons. Inspired by cationic C–H anion acceptors in supramolecular chemistry (Fig. 1b)^30,31^, these tectons encapsulate POMs within V-shaped pockets through electrostatic interactions and C–H···anion hydrogen bonds (Fig. 1c)^32–34^. This strategy addresses any size mismatch and promotes a self-adjusting assembly into porous POM–organic frameworks (POFs; Fig. 1c), where the rigid POMs confer high stability^35^. Unlike CPOSs and HOFs, which rarely undergo post-synthetic modifications (PSMs)^36,37^, POFs provide a robust platform for further functionalization.

**Extended Data Fig. 1:**
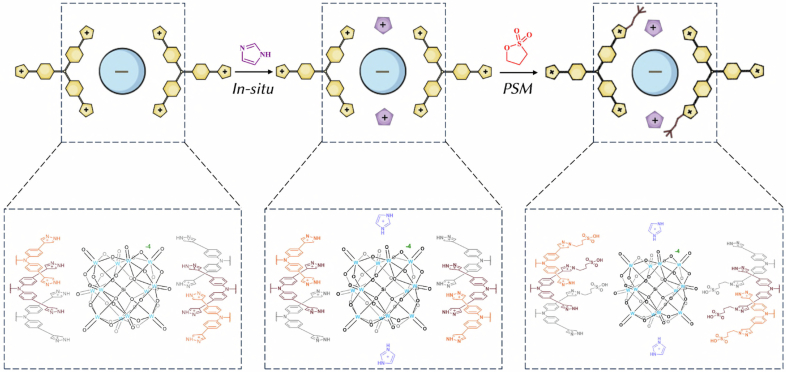
Charge balance of the SiW-POF series during stepwise synthesis. In SiW-POF1, the formal −4 charge of {SiW_12_} is compensated by the adjacent protonated triazolium on cationic TTPA tecton. In SiW-POF2(0.3), the charge neutrality is achieved by both the protonated triazolium on the cationic TTPA tecton and imidazolium incorporated during *in situ* assembly. In SiW-POF2(0.3)-S(60%), the negative charge is compensated by sulfonated and protonated triazolium units on the TTPA tecton together with imidazolium. The colour scheme is consistent with Fig. 1.

We report, here, a family of porous POFs constructed from trigonal tecton tris(4-(4*H*-1,2,4-triazol-4-yl)phenyl)amine (TTPA) and Keggin-type POMs {PW_12_}/{SiW_12_}/{BW_12_} ({PW_12_} = [PW_12_O_40_]^3−^, {SiW_12_} = [SiW_12_O_40_]^4−^, {BW_12_} = [BW_12_O_40_]^5−^) (Fig. 1c and Extended Data Fig. 1). These robust frameworks facilitate in situ imidazolium incorporation and subsequent post-synthetic sulfonation (Fig. 1c). Their permanent porosity was confirmed through CO_2_, SO_2_, NH_3_ and water vapour adsorption, alongside dye uptake. The sulfonated POFs exhibit high crystallinity, with one-dimensional (1D) channels lined by pendant alkyl sulfonic acids, enabling dual-channel proton transport via synergistic hydrogen-bonded networks. This yielded a superprotonic conductivity of 7.04 × 10^−2^ S cm^−1^ at 85 °C and 98% relative humidity (RH). When incorporated into Nafion, the hybrid membranes achieved a maximum current density of 3,400 mA cm^−2^ and power density of 1,367 mW cm^−2^ at 85 °C and 100% RH, demonstrating their potential to advance PEMFC technology.

## Results and discussion

### Molecular design

**Fig. 2:**
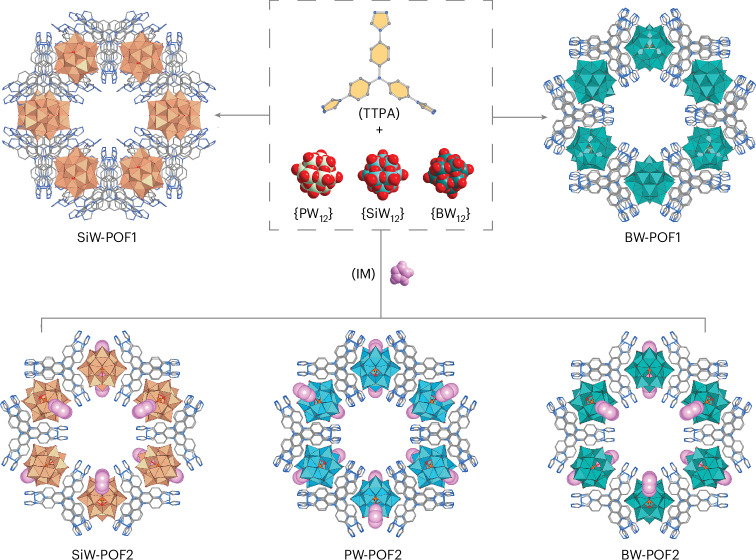
Synthetic routes for POF1 and POF2 and their porous structures. Cyan, tan and green, W; red, O; blue, N; light grey, C; light purple, imidazolium (IM).

Figure 2 outlines the one-pot hydrothermal synthesis of POFs, which comprises heating an aqueous suspension of H_4_[SiW_12_O_40_]/H_3_[PW_12_O_40_]/H_5_[BW_12_O_40_] with TTPA (0.83 ratio) at 180 °C for three days. This yields two families: POF1 (imidazolium-free; SiW-POF1, BW-POF1) and POF2 (imidazolium-containing; PW-POF2, SiW-POF2, BW-POF2). Under hydrothermal conditions, proton transfer from POM clusters to TTPA generates a cationic tecton that directs assembly via electrostatic interactions and C–H···O hydrogen bonds within V-shaped pockets (Extended Data Fig. 1), enabling the size-matched assembly of porous frameworks. Imidazolium acts as a structure-directing agent, promoting POF2 structures with hexagonal 1D channels. Varying the imidazole/TTPA ratio (0.1–2) yields POF2(X), as quantified by ^1^H NMR of digested samples. Post-synthetic modification using 1,3-propanesulfone affords sulfonated derivatives POF2(X)-S; for example, SiW-POF2(0.3)-S(60%) corresponds to 60% sulfonation of triazole units. Under strongly acidic conditions, nonporous POF3 phases (PW-POF3, SiW-POF3, BW-POF3) form due to strong N–H···O interactions. Systematic variation of tecton symmetry and geometry (Supplementary Figs. 3–5 and Supplementary Table 1) demonstrates that trigonal topology, V-shaped pockets and size-matching are essential for porous-framework formation. Template screening showed that only imidazole and its derivatives direct POF2 formation, with other N-heterocycles yielding unidentified solids (Supplementary Figs. 6 and 7). Supplementary Figs. 20–27 and Supplementary Figs. 30–37 present powder X-ray diffraction (PXRD) patterns and Fourier-transform infrared (FTIR) spectra of the POFs.

Single-crystal X-ray diffraction (SCXRD) images show that SiW-POF1 crystallizes in the monoclinic space group *C*2/*m* (Supplementary Table 2). The asymmetric unit comprises three halves of crystallographically independent [SiW_12_O_40_]^4−^ anions, four-and-a-half TTPA and five water molecules (Supplementary Fig. 8). All TTPA tectons adopt trigonal configurations with arms twisted out of the central plane (Supplementary Fig. 9)^38^. Assembly is governed by electrostatic interactions and C–H···O hydrogen bonds (H···O of 2.01–2.69 Å) between {SiW_12_} and TTPA (Supplementary Fig. 10 and Supplementary Table 5). Although a short contact is observed between the triazole ring and {SiW_12_} (C95–H95···O66, H···O = 2.01 Å), most interactions are weak (H···O ≥ 2.2 Å). The N–H···O interaction between protonated TTPA and {SiW_12_} is also weak (H···O = 2.67 Å). The presence of five distinct TTPA types leads to asymmetric packing and irregular hexagonal 1D channels along the *c* axis (10.2 Å × 7.8 Å; Fig. 2 and Supplementary Fig. 11).

In contrast to SiW-POF1, BW-POF1 crystallizes in the higher-symmetry *R*–3*m* space group (Supplementary Table 2). Its asymmetric unit comprises one-quarter of a [BW_12_O_40_]^5−^, half a TTPA and two water molecules (Supplementary Fig. 12). The *C*_3_-symmetric arrangement of TTPA enables uniform entrapment of {BW_12_} clusters within its three pockets by C–H···O interactions (2.11–2.62 Å; Supplementary Table 6). This ordered assembly yields regular 1D hexagonal channels (11.6 Å) along the *c* axis (Fig. 2 and Supplementary Fig. 12). Compared to SiW-POF1, the single TTPA environment produces more open channels, highlighting the role of tecton symmetry in controlling pore geometry.

The introduction of imidazolium yields isostructural POF2 frameworks (Fig. 2). SiW-POF2(0.3) crystallizes in the *R*–3*m* space group (Supplementary Table 2), with an asymmetric unit containing one-quarter [SiW_12_O_40_]^4−^, half a TTPA and half an imidazolium (Supplementary Fig. 13). Protonation occurs preferentially on imidazole, followed by TTPA, to balance the framework charge. The structure retains hexagonal 1D channels (11.6 Å) similar to those of BW-POF1, constructed from C–H···O interactions between TTPA and {SiW_12_} (2.10–2.63 Å; Supplementary Table 7). Imidazolium is anchored between {SiW_12_} clusters by N–H···O hydrogen bonds (2.54–2.66 Å; Supplementary Fig. 14 and Supplementary Table 7). Similar hydrogen-bonding patterns are observed in PW-POF2(0.3) and BW-POF2(0.3) (Supplementary Figs. 15 and 16 and Supplementary Tables 8 and 9). This architecture strengthens the hydrogen-bonding network while preserving channel accessibility for further modification.

In contrast to porous POF1 and POF2, BW-POF3 exhibits nonporous, dense packing (Supplementary Fig. 18). Although weak C–H···O interactions (2.16–2.69 Å) are present, strong N–H···O interactions (1.85 Å) between TTPA and {BW_12_} clusters and N–H···N interactions (1.83–1.89 Å) between neighbouring TTPA tectons dominate the assembly (Supplementary Table 10). These short hydrogen bonds, comparable to nonporous POM-guanidium frameworks and pyrazole-based HOFs^36,37^, disrupt the formation of hexagonal channels and result in a condensed network structure^39,40^. Similarly dense, nonporous structures are observed for SiW-POF3 and PW-POF3 (Supplementary Fig. 19).

Owing to their highly charged, polar inner surfaces, POFs exhibit negligible affinity for nonpolar N_2_ (Supplementary Fig. 91), but enhanced adsorption of polar or polarizable guests^41^. SiW-POF1 and SiW-POF2(0.3) show CO_2_ uptakes of 0.38 mmol g^−1^ and 0.54 mmol g^−1^, respectively, at 1 bar and 25 °C (Supplementary Fig. 92). This behaviour is further corroborated by the higher uptake of more polar NH_3_ (2.80 mmol g^−1^) and SO_2_ (1.18 mmol g^−1^) in SiW-POF2(0.3) at 25 °C (Supplementary Figs. 94 and 96). At 0 °C, both gases retain their characteristic type-I isotherms with increased capacities (4.20 mmol g^−1^ for NH_3_ and 1.58 mmol g^−1^ for SO_2_; Supplementary Figs. 95 and 97). Correspondingly, SiW-POF1 shows moderate uptakes of 3.5 mmol g^−1^ and 1.33 mmol g^−1^. These results confirm that POFs are inherently microporous and preferentially adsorb polar guests. This extends to water vapour, where SiW-POF2(0.3) exhibits a type-I + IV isotherm with a capacity of 0.15 g g^−1^ at 25 °C (Supplementary Fig. 98). Channel accessibility is further demonstrated by solution-phase uptake of bulky cationic dye (*trans*-4-[4-(dimethylamino)styryl]-1-methylpyridinium iodide; 13.7 Å × 6.6 Å) into SiW-POF2(0.3) (Supplementary Figs. 100–104).

### Post-synthetic modification of POFs

To enable PSM, we first established the chemical and thermal stabilities of the POF1 and POF2 series. SiW-POF1 and SiW-POF2 retain their crystallinity across pH 1–11 and in boiling water, with minimal changes in PXRD patterns (Supplementary Figs. 28 and 29). With thermal stability extending to ~350 °C (Supplementary Figs. 38–45), these frameworks remain robust beyond the typical operating temperature of modification (that is, <100 °C), making them good candidates for chemical modifications.

The uniformly aligned N sites of TTPA along the 1D channels provide ideal reactive sites for post-synthetic grafting of sulfonates onto the porosity^42,43^. We thus functionalized SiW-POF2(0.3) using 1,3-propanesulfone to produce a series of modified frameworks, SiW-POF2(0.3)-S, with tunable sulfonation (17–60%), as determined by ^1^H NMR (Supplementary Figs. 69–72). Due to its superior proton conductivity, SiW-POF2(0.3)-S(60%) was selected for further study. NMR analysis confirmed that sulfonation selectively targets triazole groups on TTPA, with no modification on imidazolium (Supplementary Figs. 74 and 75). We attribute this to steric hindrance around the imidazole N sites, rendering them inaccessible to the 1,3-propanesulfone (Supplementary Fig. 17).

**Fig. 3:**
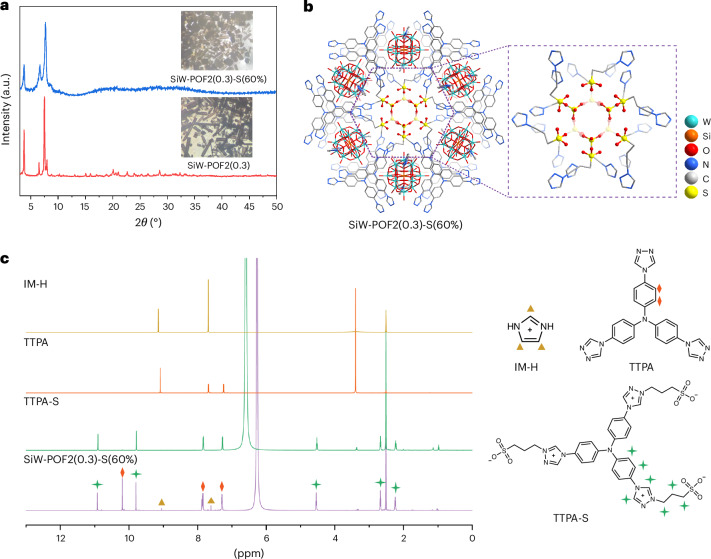
Structural characterization of SiW-POF2(0.3)-S(60%). **a**, PXRD patterns of SiW-POF2(0.3) and SiW-POF2(0.3)-S(60%). **b**, Crystal structure showing alkyl sulfonic acids within the 1D channels of SiW-POF2(0.3)-S(60%) (150 K, 40% RH). **c**, ^1^H NMR spectra of SiW-POF2(0.3)-S(60%), imidazolium chloride, TTPA and TTPA-S. Source data

PXRD patterns confirmed that crystallinity was preserved after modification (Fig. 3a), and SCXRD analysis revealed a disordered yet dense distribution of alkyl sulfonic acids within the 1D channels (Fig. 3b and Supplementary Table 12). ^1^H NMR spectra of digested samples confirmed a sulfonation ratio of 60% (Fig. 3c) compared with imidazolium, TTPA and TTPA-S. Meanwhile, the FTIR spectra show characteristics for S=O bands at 1,042 and 1,190 cm^−1^ (Supplementary Fig. 76)^44,45^. Thermogravimetric analysis indicated that increasing sulfonation leads to higher mass loss, and the fundamental thermal stability remains robust up to >350 °C (Supplementary Fig. 77). Although functionalization substantially reduces CO_2_ uptake, water adsorption remains largely intact (Supplementary Figs. 92 and 98), suggesting that the channels remain permeable to small polar molecules for ion transport. The framework also displays humidity-dependent swelling; the unit cell volume expands from 24,704 Å^3^ to 25,800 Å^3^ as the RH increases from 40% to 98% (Supplementary Table 13), accompanied by increased water adsorption and a more extensive hydrogen-bonded water network (Supplementary Fig. 67). This structural flexibility supports efficient water transport within the channels.

### Fabrication and characterization of hybrid POF@Nafion membrane

**Fig. 4:**
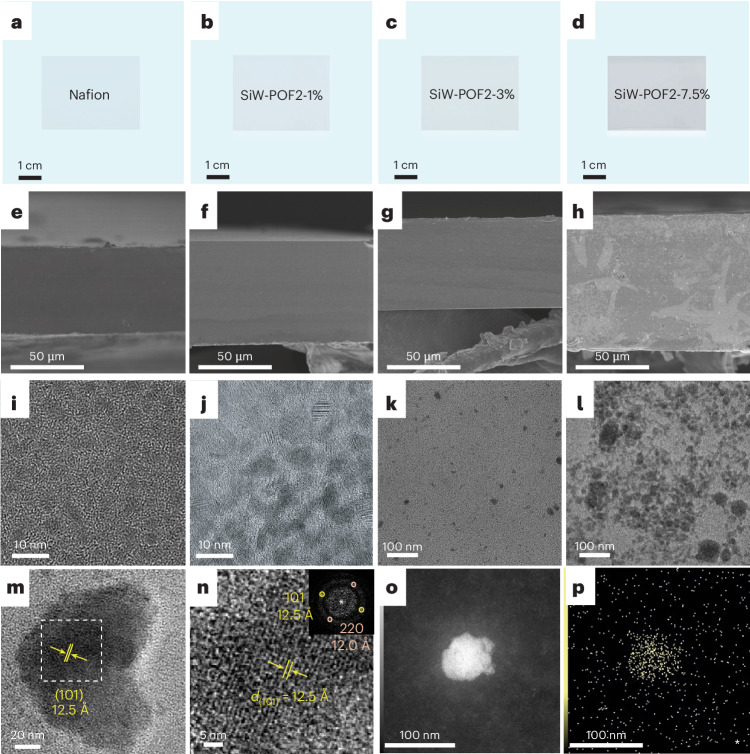
Macroscopic morphology and microphase structure of hybrid membranes. **a**–**d**, Photographs of Nafion and SiW-POF2-S(60%)@Nafion membranes; 1–7.5% represent mass ratio of SiW-POF2-S(60%) within Nafion matrix. **e**–**h**, Cross-sectional SEM images of the corresponding membranes in **a**–**d**, respectively. **i**–**l**, HRTEM images of Nafion (**i**), SiW-POF2-S(60%)@Nafion-3% (**j**,**k**) and SiW-POF2-S(60%)@Nafion-7.5% (**l**). **m**, HRTEM image of a SiW-POF2-S(60%) nanoparticle in the 3 wt% membrane. **n**, Magnified HRTEM image of the white-boxed region in **m**, showing the lattice fringe of the (101) plane. Inset: FFT pattern (zone axis $(1\bar{1}\bar{1})$). **o**, HAADF-STEM image of a nanoparticle and surrounding region in 3 wt% hybrid membrane. **p**, EDX mapping of S.

Mixing SiW-POF2(0.3)-S(60%) with Nafion stock solution followed by casting and heating at 100 °C for 12 h yielded hybrid SiW-POF2-S(60%)@Nafion-*x*% (*x* = 1, 2, 3, 4, 5, 7.5, representing the mass ratio of SiW-POF2-S(60%)). The membranes showed macroscopic transparency at loadings below 3 wt%, becoming translucent at 3 wt% and opaque at higher loadings (Fig. 4a–d and Supplementary Fig. 107). Cross-sectional scanning electron microscopy (SEM) images show a uniform surface topography at 1–3 wt% (Fig. 4e–g), but interconnected morphology at higher loadings (4–7.5 wt%) (Fig. 4h and Supplementary Fig. 107). PXRD analysis confirmed the emergence of the SiW-POF2-S(60%) crystalline phase at a loading of ≥4 wt% (Supplementary Fig. 108). Compared to pristine Nafion, hybrid membranes display enhanced breaking strength, but with slightly reduced elongation (Supplementary Fig. 109 and Supplementary Table 21). These results identify 3 wt% as the optimal loading to maintain macroscopic homogeneity while providing sufficient proton-conductive sites and pathways.

At the nanoscale, high-resolution transmission electron microscopy (HRTEM) images reveal uniform dispersion of SiW-POF2-S(60%) nanoparticles and enhanced fusion of Nafion ionic nanophases (dark spheres) into a continuous interconnected network at 3 wt% (Fig. 4i,j). This structural evolution probably enhances the crystallinity of Nafion, providing extensive proton-transport networks^46^. Nanoscale SiW-POF2-S aggregates are evenly distributed within Nafion, with an average size of 16.63 ± 5.25 nm (Fig. 4k and Supplementary Fig. 110), although larger nanoparticles (50–80 nm) are occasionally observed (Supplementary Fig. 111). Higher loadings lead to aggregation and macroscopic phase separation (Fig. 4h,l and Supplementary Figs. 107 and 112). HRTEM analysis of individual SiW-POF2-S(60%) nanoparticles within the 3% membrane shows clear lattice fringes, confirming the retention of a single-crystalline porous structure (Fig. 4m). Fast Fourier transform (FFT) analysis yielded lattice spacings of 12.5 and 12.0 Å for the (101) and (220) planes (Fig. 4n). High-angle annular dark-field scanning transmission electron microscopy (HAADF-STEM) and energy dispersive X-ray spectroscopy (EDX) mapping images show sulfur enrichment around nanoparticles relative to the Nafion matrix (Fig. 4o,p), indicating that surface alkyl sulfonic acids extend outward to interact with Nafion pendant sulfonate groups, bridging ionic nanophases to form a more continuous proton-conducting network. Atomic force microscopy and small-angle X-ray scattering confirmed that loadings of 1–3 wt% preserve intrinsic hydrophobic/hydrophilic nanophase separation of Nafion (Supplementary Figs. 114 and 115)^47^.

### Proton conductivity and fuel-cell performance of hybrid membrane

**Fig. 5:**
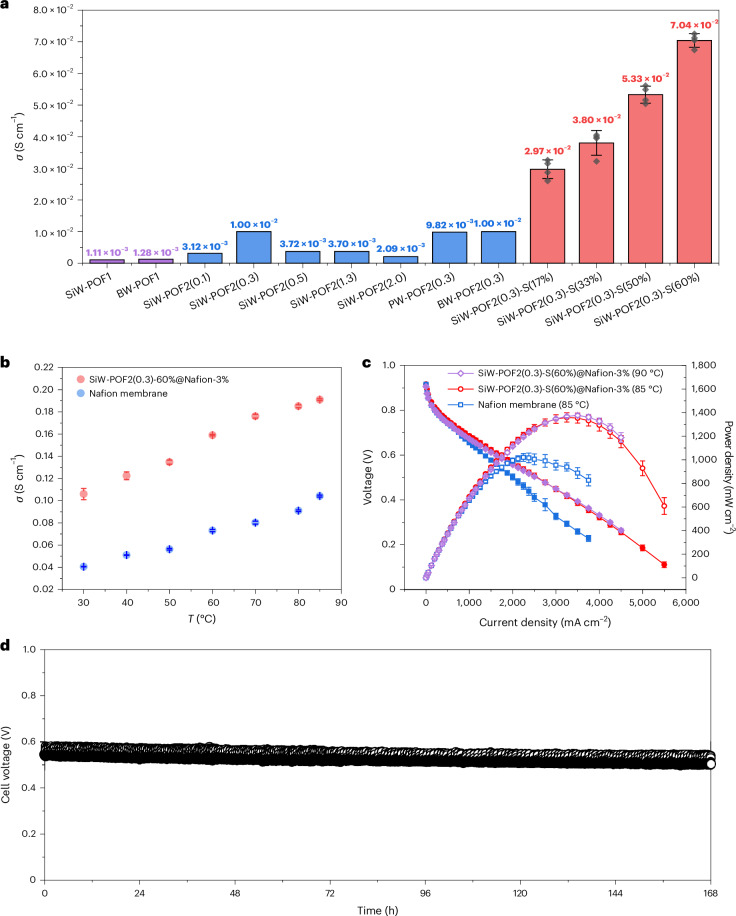
Proton conducting and fuel-cell performance of POFs and POF@Nafion membranes. **a**, Proton conductivities of the POF1 series (purple), POF2 series (blue) and POF2-S series (red) at 85 °C and 98% RH. **b**, Temperature-dependent proton conductivity of Nafion and SiW-POF2(0.3)-S(60%)@Nafion-3% (30–85 °C, 98% RH). **c**, H_2_–O_2_ fuel-cell performance of Nafion and SiW-POF2(0.3)-S(60%)@Nafion-3% at 85 °C and 90 °C. The open and closed symbols represent power density curve and polarization curve, respectively. The flow rates of H_2_ and O_2_ are 0.3/0.3 l min^−1^. **d**, Durability of the SiW-POF2(0.3)-S(60%)@Nafion membrane at 100 mA cm^−2^ over 168 h at 85 °C and 100% RH. Flow rates are as in **c**. Data are presented as mean ± s.d. (*n* = 4) in **a** and as mean ± s.d. (*n* = 3) in **b**,**c**. Source data

**Extended Data Fig. 2:**
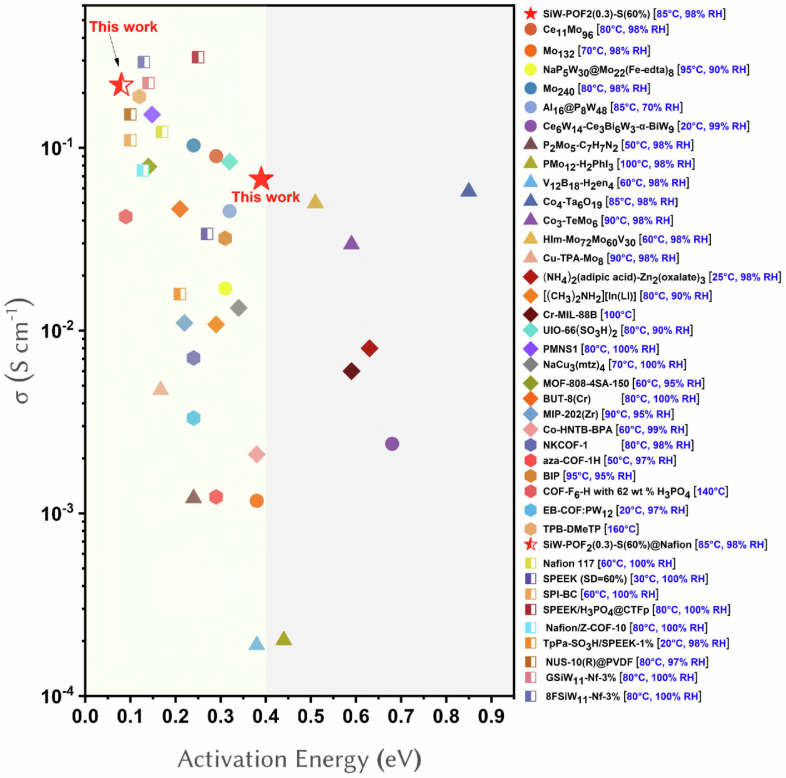
Performance comparison of SiW-POF2(0.3)-S(60%) and SiW-POF2(0.3)-S(60%)@Nafion-3% membrane with representative crystalline solid conductors and membrane-based conductors. The figure benchmarks proton conductivity and *E*a. The crystalline solid conductors are represented in fully-filled symbols, while membrane-based conductors are denoted by half-filled symbols. All comparative data are adapted from references (refs. ^19–54^) in Supporting Information. Source data

The functionalized channels provide a dense matrix for proton conduction, as confirmed by an alternating current impedance test at 85 °C and 98% RH (Fig. 5a, Supplementary Figs. 54–62 and Supplementary Tables 17–20). The POF2 series exhibit higher conductivities than members of the POF1 series, which is attributed to the presence of imidazolium (Fig. 5a) Conversely, the nonporous POF3 series show substantially lower values (Supplementary Tables 17 and 18), highlighting the importance of porosity. Optimizing the imidazolium content (0.1–2.0 equiv. relative to TTPA) yielded a maximum conductivity of 1.00 × 10^−2^ S cm^−1^ for SiW-POF2(0.3), an ~9-fold increase over SiW-POF1 (Fig. 5a and Supplementary Fig. 57). PW-POF2(0.3) and BW-POF2(0.3) exhibited analogous values (Supplementary Table 18). Post-synthetic sulfonation further enhanced performance, with SiW-POF2(0.3)-S(60%) reaching 7.04 × 10^−2^ S cm^−1^, a 700-fold increase over SiW-POF1 (Fig. 5a and Supplementary Table 19). Supplementary Fig. 81 shows the impedance spectra of SiW-POF2(0.3)-S(60%) under 98% RH at varying temperatures. These values are competitive with leading POM-based conductors, CPOSs, MOFs, COFs, HOFs, polymer membranes, composite systems and commercial Nafion (Extended Data Fig. 2)^46–52^. Arrhenius plots show activation energies (*E*_a_) of 1.15 eV for SiW-POF2(0.3) and 0.39 eV for SiW-POF2(0.3)-S(60%) (Supplementary Figs. 58 and 84), indicating a transition from vehicle to Grotthuss transport upon sulfonation. This is supported by the isotope effect, which reveals a proton/deuteron conductivity, *σ*_Η+_/*σ*_D+_, of 1.42, closely matching the theoretical value (1.41) for proton hopping (Supplementary Fig. 87)^53^. Single-crystal proton-conductive measurements of SiW-POF2(0.3) show strong anisotropy, with conductivity along the *c* axis ~200 times higher than in the perpendicular directions (Supplementary Figs. 60 and 61), indicating that primary proton transport occurs along the *c* axis. Despite the lack of data for SiW-POF2(0.3)-S(60%), its close structural relationship to SiW-POF2(0.3) suggests a similar proton pathway. PXRD confirmed its structural integrity before and after impedance analysis (Supplementary Fig. 89).

Leveraging the exceptional conductivity of SiW-POF2(0.3)-S(60%), we evaluated hybrid SiW-POF2-S(60%)@Nafion membranes for proton transport and H_2_–O_2_ fuel-cell performance. The 3 wt% hybrid membrane exhibited optimal conductivity across 30–85 °C and 50–98% RH (Fig. 5b and Supplementary Fig. 116), reaching 0.191 S cm^−1^ at 85 °C and 98% RH, which is 84% higher than for Nafion (0.104 S cm^−1^), and exceeding the performance of most reported POM-doped Nafion membranes (Supplementary Table 25)^54–57^. The reduction in *E*_a_ from 0.17 eV (Nafion) to 0.13 eV (hybrid membrane) (Supplementary Fig. 117) indicates facilitated proton transport arising from synergistic interactions between SiW-POF2(0.3)-S(60%) and Nafion, which promote the percolation of proton-conducting channels and improve water retention (Supplementary Fig. 118 and Supplementary Note 8). A control {SiW_12_}@Nafion-3% hybrid membrane showed rapid conductivity decay within 4 h (Supplementary Fig. 119), confirming the immobilizing stability of the POF. H_2_–O_2_ fuel-cell testing (85–95 °C, 100% RH) delivered a peak current density of 3,400 mA cm^−2^ and power density of 1,367 mW cm^−2^ at 85 °C, as well as an open-circuit voltage of 0.789 V, indicating a 133% enhancement over Nafion and excellent hydrogen resistance (Fig. 5c)^46,58^. Long-term operation at 85 °C and 100% RH (100 mA, 168 h) showed a low voltage decay rate of 0.23 mV h^−1^ and a total loss of 6.76% (Fig. 5d). The membrane remained stable at elevated temperatures, achieving highest peak current and power density values of 3,400 mA cm^−2^ and 1,407 mW cm^−2^, respectively, at 90 °C, with long-term stability (Fig. 5c and Supplementary Figs. 123 and 124). The membrane also showed strong chemical stability across pH 0–11, as well as oxidative resistance (Supplementary Figs. 120 and 121), with only 4.8% weight loss after Fenton treatment compared to 21% for pristine Nafion (Supplementary Fig. 122). Under low humidity (40% RH), the hybrid membrane delivered a power density of 680 mW cm^−2^ and current density of 1,800 mA cm^−2^, a 147% improvement over Nafion (Supplementary Fig. 125).

### Mechanistic insights

**Fig. 6:**
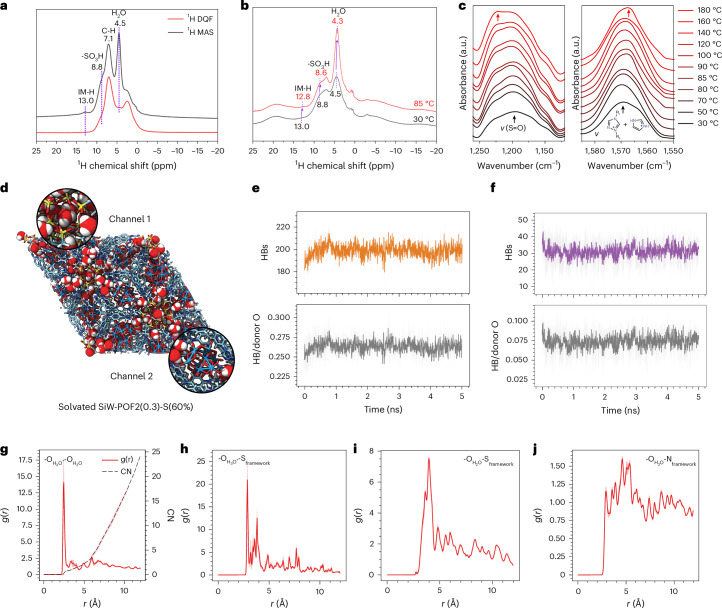
Mechanistic insights into proton conduction in SiW-POF2(0.3)-S(60%). **a**, ^1^H MAS and ^1^H DQF NMR spectra. **b**, Temperature-dependent ^1^H MAS NMR spectra (30 and 85 °C). **c**, Variable-temperature IR spectra highlighting sulfonate and imidazolium/triazolium vibrations. **d**, Representative equilibrated host–guest configuration under equilibrium hydration, showing two crystallographically distinct channels. **e**,**f**, Time evolution of the total hydrogen bond population (top) and the number of hydrogen bonds (HB) per donor oxygen (bottom) for channel 1 (**e**) and channel 2 (**f**). **g**, O(H_2_O)-O(H_3_O^+^) RDF with peaks at ~2.4 Å and ~2.7–2.8 Å. **h**, O(H_3_O^+^)-S(framework) RDF showing a strong first-shell peak at ~2.5–2.6 Å. **i**, O(H_2_O)-S(framework) showing weak ordering. **j**, O(H_2_O)-N(framework) showing diffuse, non-specific correlations. Source data

To probe the proton dynamics and transfer pathways in SiW-POF2(0.3)-S(60%), we performed solid-state ^1^H magic-angle spinning (^1^H MAS) NMR. The obtained spectrum exhibits broad peaks at 13.0, 8.8, 7.1 and 4.5 ppm, assigned to imidazolium N–H, -SO_3_H, aromatic C–H and adsorbed water, respectively (Fig. 6a and Supplementary Fig. 105)^59–61^. Double-quantum filtered (DQF) spectra show a notable decrease in the signals at 13.0, 8.8 and 4.5 ppm, indicating weak ^1^H–^1^H dipole–dipole coupling due to rapid proton motion^62^. Temperature-dependent spectra (Fig. 6b) show slight downfield shifts and pronounced linewidth narrowing for imidazolium, -SO_3_H and H_2_O resonances up to 85 °C. This indicates accelerated proton exchange between these species, contributing to proton conductivity. Conversely, the aromatic C–H signal at 7.1 ppm remains unchanged in both ^1^H DQF and temperature-dependent spectra, confirming its minimal contribution to conductivity. Variable-temperature infrared spectra show a blueshift of the S=O vibration and redshifts of imidazolium/triazolium bands (Fig. 6c), evidencing the facile proton dissociation from sulfonic acid and N-heterocycles that enables efficient proton hopping^63,64^.

**Extended Data Fig. 3:**
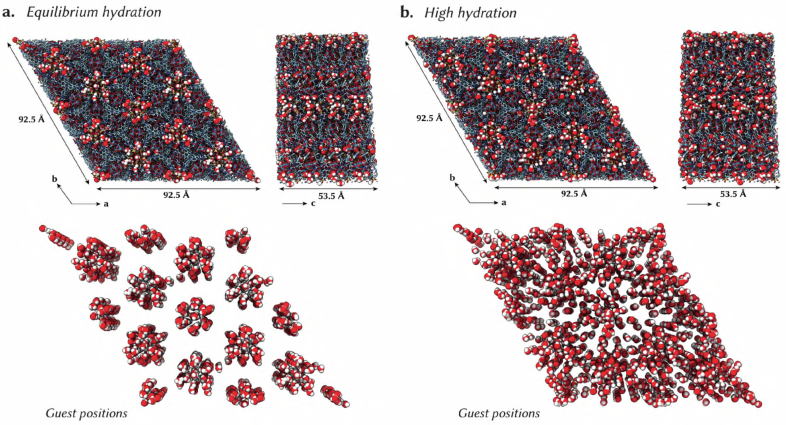
SiW-POF2(0.3)-S(60%) supercell used for MD calculations. The supercell is a 2 × 2 × 4 replicate of the experimental unit cell, consisting of ~33,000 atoms. The guest positions are calculated in two hydration regimes. **a**. Equilibrium hydration, wherein GCMC simulations are performed (n = 3) to calculate the equilibrium number of H_2_O molecules in the host at 94% RH and 358 K, while the number of H_3_O^+^ molecules are fixed to ensure charge neutrality of the system. In this regime, the guests preferentially cluster around the sulfonate-rich channel (Channel 1) of SiW-POF2(0.3)-S(60%). **b**. High hydration, where fixed loading MC simulations are performed (n = 3) to calculate the equilibrium configurations of H_2_O and H_3_O^+^ molecules in the host at 358 K. In this regime, the guests occupy both channels SiW-POF2(0.3)-S(60%).

To elucidate the molecular origins of proton transport in SiW-POF2(0.3)-S(60%), we combined grand canonical Monte Carlo (GCMC) simulations to establish equilibrium and high hydration conditions with classical molecular dynamics (MD) simulations to probe the structures and dynamics of confined H_2_O and H_3_O^+^. Representative host–guest configurations obtained from GCMC at 94% RH and 358 K were replicated into a supercell and propagated under periodic boundary conditions. Figure 6d shows a representative unit cell under equilibrium hydration, and Extended Data Fig. 3 illustrates the scale of the constructed supercell and guest configurations under both hydration regimes. The equilibrium guest configurations qualitatively match the guest positions from the experimental single-crystal structure. The framework was treated as rigid, with fully mobile H_2_O and H_3_O^+^, enabling analysis of hydrogen-bond networks, relaxation dynamics and the local solvation structure. Charge neutrality was enforced by assigning negative charge to deprotonated sulfonate oxygens.

Under equilibrium hydration, the simulations revealed notable spatial heterogeneity in hydrogen-bond connectivity between the two crystallographically distinct channels. The sulfonate-rich channel 1 sustains a highly connected and persistent hydrogen-bond network (Fig. 6d,e), whereas channel 2 exhibits a weaker, fragmented network (Fig. 6d,f). Channel-resolved analysis over 5-ns production trajectories (*n* = 3) showed that channel 1 maintains ~200 hydrogen bonds (~0.26 per donor oxygen) within the channel volume (Fig. 6e), whereas channel 2 sustains only ~30–35 hydrogen bonds (~0.07–0.08 per donor oxygen), indicating limited connectivity (Fig. 6f). These stationary statistics support that sulfonation selectively stabilizes a percolated hydrogen-bond network in channel 1, whereas channel 2 remains below the connectivity threshold for continuous pathways, consistent with the reduced activation energy observed for post-sulfonation.

The relaxation dynamics of the confined fluid were assessed using velocity and orientational autocorrelation functions (Supplementary Note 7). Velocity autocorrelation functions decay on ultrafast timescales for both H_2_O and H_3_O^+^, indicating rapid local momentum relaxation (Supplementary Fig. 127). Slower collective dynamics were probed by orientational autocorrelation functions, which capture dipole reorientation and hydrogen-bond network restructuring. Both species exhibited a rapid subpicosecond decay associated with librational motion, followed by convergence to a long-time non-zero plateau within ~1–2 ns (Supplementary Fig. 127). The plateau reflects persistent orientational ordering under confinement^65,66^, and the corresponding timescale defines the slowest collective relaxation mode of the confined fluid.

Within the equilibrated hydrogen-bond environment, H_3_O^+^ ions adopt a mixed local solvation structure. O(H_2_O)-O(H_3_O^+^) radial distribution functions (RDFs) show a pronounced first peak at ~2.4–2.5 Å (Fig. 6g), consistent with Zundel-like (H_5_O_2_^+^) and Eigen-like (H_9_O_4_^+^) motifs^67,68^. Their coexistence indicates that excess protons are not locked in a single solvation geometry, but instead fluctuate between localized and shared configurations. These motifs are characteristic precursors for proton hopping in hydrogen-bonded networks and demonstrate that confinement preserves the local environments required for proton transfer^69^. O(H_3_O^+^)-S(framework) correlations show an intense, first-shell peak at ~2.5–2.6 Å (Fig. 6h), indicating preferential localization of H_3_O^+^ near deprotonated sulfonate oxygens in channel 1. Conversely, O(H_2_O)-S(framework) correlations are weaker (Fig. 6i), highlighting selective hydronium localization. Interactions of H_2_O and H_3_O^+^ with the framework N and O are diffuse and non-specific (Fig. 6j and Supplementary Fig. 126), indicating limited structural ordering outside sulfonate-rich regions. Under high hydration (Supplementary Fig. 128), the O(H_2_O)-O(H_3_O^+^) and O(H_3_O^+^)-S(framework) correlations persist, confirming preservation of local solvation and proton–sulfonate interactions. Medium-range features become more pronounced due to the increased guest density, especially in channel 2.

The simulations show that sulfonation induces a channel-selective increase in hydrogen-bond connectivity, forming a percolated network in channel 1 that provides structural preconditions for Grotthuss-type proton hopping. In contrast, channel 2 lacks continuous pathways and supports only localized, weakly connected water clusters. Although explicit proton transfer was not modelled, the combined analysis of channel-resolved statistics, global relaxation dynamics, and hydronium solvation explains the reduced activation energy upon sulfonation, indicating that proton transport is governed by dynamically connected hydrogen-bonded networks under confinement.

## Conclusion

We have presented a supramolecular engineering strategy for constructing porous POFs through programmable electrostatic assembly of POMs and trigonal-shaped cationic tectons. By leveraging size-matched interactions and directing C–H···anion hydrogen bonding, we have achieved robust frameworks with well-defined 1D channels. These charge-complementary architectures, built from anion–cation pairs, are rare examples capable of sequential functionalization, enabling controllable channel polarity and ionic charge density. The integration of imidazolium and sulfonate groups into the POF architecture creates a synergistic, dense proton-conduction matrix. The optimized material, SiW-POF2(0.3)-S(60%), achieves a superprotonic conductivity of 7.04 × 10^−2^ S cm^−1^ at 85 °C and 98% RH, rivalling state-of-the-art conductors. Spectral analysis and MD simulations show that post-synthetic sulfonation induces channel-selective hydration and stabilizes percolated hydrogen-bond networks under confinement, creating structural features that favour Grotthuss-type proton transport over localized, vehicle-dominated motion. Demonstrating practical relevance, a hybrid 3% POF@Nafion membrane achieves a power density of 1,367 mW cm^−2^ in H_2_–O_2_ fuel cells, a 33% improvement over commercial Nafion, with exceptional durability under operational conditions. Our work expands the design principles of porous materials, merging high ionic density with structural precision. Beyond energy conversion, we foresee that these POFs hold promise for applications in catalysis and atmospheric water harvesting, establishing a blueprint for next-generation functional materials.

## Methods

### Synthesis of tris(4-(4H-1,2,4-triazol-4-yl)phenyl)amine (TTPA)

*N*,*N*-dimethylformamide azine dihydrochloride (1.28 g, 9 mmol), tris(4-aminophenyl)amine (0.55 g, 3 mmol), dimethylformamide (DMF; 10 ml) and pyridine (10 ml) were added to a 50-ml pear-shaped bottle equipped with a condenser and a stir bar. The reaction mixture was refluxed at 130 °C for 84 h. After cooling the reaction mixture to room temperature, the solvent was removed by filtration, and the crude product was washed with acetonitrile (3 × 20 ml) and then placed in an oven at 80 °C and dried for one day to obtain a pale yellow product, TTPA (yield: 0.36 g, 40%; ^1^H NMR (600 MHz, DMSO-*d*_6_): 9.08 (s, 6 H), 7.69 (d, *J* = 8.4 Hz, 6 H), 7.24 (d, *J* = 8.4 Hz, 6 H))^70^.

### Synthesis of SiW-POF1 and other POF1 derivatives

H_4_SiW_12_O_40_ (120 mg, 0.0414 mmol) and TTPA (22.5 mg, 0.05 mmol) were placed into 5 ml of deionized water. After stirring for 0.5 h, the pH value of the mixture was adjusted to 5.5 with 1 M NaOH. Afterwards, the mixture was transferred into a 10-ml Teflon reactor and heated for 72 h at 180 °C under autogenous pressure. After cooling to room temperature, brown strip-shaped crystals were obtained by filtration and washed with deionized water. Yield: 63 mg, 87%. Elemental analysis % calcd (found): C 19.83 (19.86), H 1.70 (1.74), N 9.64 (9.79), Si 0.64 (0.61), W 50.67 (50.87). Formula: H_6_{(C_24_H_18_N_10_)_4.5_(SiW_12_O_40_)_1.5_}·12H_2_O. BW-POF1 was synthesized in the same way but substituting H_4_SiW_12_O_40_ (120 mg, 0.0414 mmol) with H_5_BW_12_O_40_ (120 mg, 0.0414 mmol)^71^. Yield: 60.0 mg, 61%. Elemental analysis % calcd (found): C 14.52 (14.59), H 1.64 (1.67), N 7.06 (7.10), B 0.28 (0.27), W 55.67 (55.72). Formula: H_5_{(C_24_H_18_N_10_)_2_(BW_12_O_40_)}·12H_2_O.

### Synthesis of SiW-POF2(0.3) and other POF2 derivatives

H_4_SiW_12_O_40_ (120 mg, 0.0414 mmol), TTPA (22.5 mg, 0.05 mmol) and imidazole (1 mg, 0.015 mmol) were placed into 5 ml of deionized water. After stirring for 0.5 h, the pH value of the mixture was adjusted to 5.5 with 1 M NaOH. Afterwards, the mixture was transferred into a 10-ml Teflon reactor and heated for 72 h at 180 °C under autogenous pressure. After cooling to room temperature, brown strip-shaped crystals were obtained by filtration and washed with deionized water. The resulted POF was denoted SiW-HPOF2(0.3). The real ratio of imidazole/TTPA was determined by ^1^H NMR when digesting the related SiW-POF2 with deuterated HCl. Yield: 53.6 mg, 50%. Elemental analysis % calcd (found): C 14.38 (14.40), H 1.94 (1.95), N 7.14 (7.12), Si 0.67 (0.66), W 53.14 (53.16). Formula: H_4_{(C_24_H_18_N_10_)_2_(C_3_N_2_H_4_)_0.6_(SiW_12_O_40_)}·19H_2_O. The synthetic conditions for SiW-POF2(0.1)/(0.5)/(1.3)/(2.0) are the same as for SiW-POF2(0.3) but with different amounts of imidazole (0.3 mg, 0.005 mmol)/(1.7 mg, 0.025 mmol)/(6.8 mg, 0.1 mmol)/(34 mg, 0.5 mmol). PW-POF2(0.3) and BW-POF2(0.3) were synthesized according to the same procedure as for SiW-POF2(0.3) but substituting H_4_SiW_12_O_40_ (120 mg, 0.0414 mmol) with H_3_PW_12_O_40_ (120 mg, 0.0414 mmol) and H_5_BW_12_O_40_ (120 mg, 0.0414 mmol), respectively. BW-POF2(1.3) was obtained in high yield by adding 2.0 equiv. imidazole (molar ratio of imidazole to TTPA) under the same conditions as for BW-POF2(0.3). PW-POF2(0.3): Yield: 38.6 mg, 36%. Elemental analysis % calcd (found): C 14.83 (14.80), H 1.62 (1.60), N 7.36 (7.38), P 0.77 (0.76), W 54.78 (54.80). Formula: H_3_{(C_24_H_18_N_10_)_2_(C_3_N_2_H_4_)_0.6_(PW_12_O_40_)}·12H_2_O. BW-POF2(0.3): Yield: 50.0 mg, 48%. Elemental analysis % calcd (found): C 14.69 (14.71), H 1.80 (1.83), N 7.30 (7.27), B 0.27 (0.26), W 54.29 (54.30). Formula: H_5_{(C_24_H_18_N_10_)_2_(C_3_N_2_H_4_)_0.6_[BW_12_O_40_]}·15H_2_O.

### Synthesis of SiW-POF3 and other POF3 derivatives

H_4_SiW_12_O_40_ (120 mg, 0.0414 mmol) and TTPA (22.5 mg, 0.05 mmol) were placed into 5 ml of deionized water. After stirring for 0.5 h, the pH value of the mixture was adjusted to 1.8 with 1 M HCl. Afterwards, the mixture was transferred into a 10-ml Teflon reactor and heated for 72 h at 180 °C under autogenous pressure. After cooling to room temperature, brown strip-shaped crystals were obtained by filtration and washed with deionized water. Yield: 33.8 mg, 48%. Elemental analysis % calcd (found): C 14.64 (14.40), H 1.47 (1.50), N 7.12 (7.36), Si 0.71 (0.66), W 56.13 (56.23). Formula: H_4_{(C_24_H_18_N_10_)_2_(SiW_12_O_40_)}·9H_2_O. PW-POF3 and BW-POF3 were synthesized according to the same procedure, but substituting H_4_SiW_12_O_40_ (120 mg, 0.0414 mmol) with H_3_PW_12_O_40_ (120 mg, 0.0414 mmol) and H_5_BW_12_O_40_ (120 mg, 0.0414 mmol) while adjusting the pH from 1.8 to 1.2 and 1.5, respectively. PW-POF3: Yield: 31.7 mg, 45%. Elemental analysis % calcd (found): C 15.19 (15.28), H 1.08 (1.23), N 7.38 (7.16), P 0.82 (0.76), W 58.23 (57.98). Formula: H_3_{(C_24_H_18_N_10_)_2_(PW_12_O_40_)}·1H_2_O. BW-POF3: Yield: 51.4 mg, 73%. Elemental analysis % calcd (found): C 20.36 (20.40), H 1.48 (1.50), N 9.90 (9.86), B 0.26 (0.25), W 52.02 (52.10). Formula: H_5_{(C_24_H_18_N_10_)_3_[BW_12_O_40_]}·2H_2_O.

### Characterization

SCXRD data were collected on a Bruker D8 VENTURE diffractometer with Mo/Cu Kα radiation at 150 K, or obtained at 173 K on the BL17B/BL18U macromolecular crystallography beamline at the National Facility for Protein Science, Shanghai Synchrotron Radiation Facility. All structures were solved and refined using standard crystallographic procedures. Full experimental and refinement details are provided in Supplementary Section 1. The infrared spectra were recorded on a NEXUS-670 FTIR spectrometer in the range of 500–4,000 cm^−1^. The PXRD data were collected on a Haoyuan DX-2700B instrument with Cu Kα radiation (*λ* = 1.54056 Å) in the range of 3° ≤ 2*θ* ≤ 50° with a scanning rate of 0.01° s^−1^. Thermogravimetric analysis was performed on a METTLER TOLEDO TG8000 thermogravimetric analyser under nitrogen flow at a typical heating rate of 10 °C min^−1^. Element analyses for P, Si, B and W were performed on a Leeman Prodigy Plus inductively coupled plasma optical emission spectroscope, and C, N and H contents were determined using a VARIDEL III elemental analyser. CO_2_ adsorption–desorption isotherms and water vapour adsorption isotherms were measured on a BSD-660M A6B6M adsorptometer (BSD Instruments). Other gas adsorptions were performed on a BSD-PM (3H-2000PM) gas adsorbent analyser. ^1^H NMR spectra were recorded on a Bruker AV 600-MHz spectrometer in DMSO-*d*_6_ with tetramethylsilane as internal standard. Confocal laser scanning microscopy micrographs were collected using an Olympus Fluoview FV1000 system with *λ*_ex_ = 450 nm. Scanning electron microscopy images were taken using a ZEISS Sigma 360 microscope. TEM images and energy dispersive spectrometer mapping were obtained using a JEOL JEM-F200 electron microscope operating at 200 kV, which was equipped with a Super-X EDS system. Aberration-corrected HAADF-STEM images were acquired using a FEI Titan cubed Themis instrument operating at 300 kV, featuring an X-FEG electron gun and a DCOR aberration corrector. Atomic force microscopy was performed by Oxford Instruments (Cypher ES). Small-angle X-ray scattering measurements were recorded on a Xeuss3.0 system with an X-ray wavelength of 1.542 Å.

#### Proton conductivity of POFs

Proton conductivity was measured on pressed pellets of POF materials using electrochemical impedance spectroscopy on a Solartron SI 1260 impedance/gain phase analyser. Impedance spectra were collected over a broad frequency range using an a.c. excitation voltage, and the resistance was extracted from the low-frequency intercept of Nyquist plots. Variable-temperature and variable-humidity measurements were performed after allowing sufficient equilibration at each condition. Activation energies for proton transport were obtained from Arrhenius analysis of the temperature-dependent conductivity data. Full experimental details, including pellet preparation, measurement conditions, impedance fitting procedures and data analysis, are provided in Supplementary Section 2. Proton conductivity experiments on selected POF-S are described in Supplementary Section 4.

#### Fabrication of POF@Nafion hybrid membranes

Hybrid proton-exchange membranes were prepared by incorporating SiW-POF2(0.3)-S(60%) into a Nafion matrix via solution casting. POF particles were dispersed in Nafion solution using a polar aprotic solvent and processed by sonication and prolonged stirring to ensure homogeneous distribution. The resulting mixtures were cast onto a flat substrate and dried under low vacuum to afford free-standing composite membranes. The POF loadings were varied to investigate the effect of filler content on membrane structure and performance. Supplementary Section 8 describes the complete characterization and experimental details of the hybrid membranes.

#### Fuel-cell measurements

Fuel-cell performance was evaluated with membrane-electrode assemblies incorporating the POF@Nafion hybrid membranes on a Scribner 850e fuel-cell system. H_2_–O_2_ fuel-cell tests were conducted under controlled temperature and humidity conditions. Long-term durability was assessed under constant-current operation. Additional measurements were performed under reduced humidity to evaluate membrane performance under more demanding conditions. Full experimental details—including electrode preparation, catalyst loadings, operating parameters and testing protocols—are provided in Supplementary Section 9.

#### Computational methods

GCMC simulations were performed in RASPA to generate representative hydrated host–guest configurations within the experimental SiW-POF2(0.3)-S(60%) framework^72^. The framework structure was geometry-optimized and subsequently treated as rigid. Guest configurations were sampled in two hydration regimes: (1) equilibrium hydration at 94% RH, where H_2_O molecules were exchanged with a reservoir, the grand canonical ensemble, and (2) high hydration, where the guest composition was fixed to match the experimentally motivated water uptake. In both cases, H_3_O^+^ ions were included to model proton defects originating from sulfonate deprotonation, with charge neutrality enforced by assigning fixed negative charge to the sulfonate groups. Following GCMC sampling, equilibrated host–guest configurations were replicated to construct large supercells for MD simulations. All-atom classical MD simulations were performed under periodic boundary conditions using large-scale atomic/molecular massively parallel simulator (LAMMPS) to characterize the hydrogen-bond networks, solvation structure and relaxation dynamics of confined H_2_O and H_3_O^+^ species^73^. Long-range electrostatics were treated using Ewald-based methods, and established non-polarizable models were used for H_2_O (SPC/E) and H_3_O^+^. The framework atoms were kept rigid, and the confined fluid was fully mobile. Production trajectories were collected over a 5-ns timescale (*n* = 3), and the convergence of key structural and dynamical observables was verified. We note that the simulations were not designed to model explicit proton-transfer events, but rather to establish the structural and dynamical preconditions—such as hydrogen-bond connectivity, solvation motifs and network stability—that underpin proton transport under confinement. Full simulation protocols, force-field parameters, equilibration procedures and analysis methods are provided in Supplementary Section 10.

## Online content

Any methods, additional references, Nature Portfolio reporting summaries, source data, extended data, supplementary information, acknowledgements, peer review information; details of author contributions and competing interests; and statements of data and code availability are available at 10.1038/s41557-026-02169-8.

## Supplementary information

Supplementary Information Supplementary Figs. 1–128, Discussion (Notes 1–8) and Tables 1–27.

## Source data

Source Data Fig. 3 PXRD and NMR source data.

Source Data Fig. 5 Proton conductivity and fuel-cell performance source data.

Source Data Fig. 6 NMR, IR and simulation source data.

Source Data Extended Data Fig. 2 Statistical performance of PEM source data.

## Data Availability

All data that support the findings of this study are available in the main text and Supplementary Information (experimental procedures and characterization data). The crystallography data in this work have been deposited in the Cambridge Crystallographic Data Centre (CCDC) under accession nos. CCDC 2330203 (SiW-POF1), 2330204 (BW-POF1), 2330202 (SiW-POF2(0.3)), 2385205 (PW-POF2(0.3)), 2330200 (BW-POF2(0.3)), 2519967 (SiW-POF3), 2519966 (PW-POF3), 2330201 (BW-POF3), 2520631 (SiW-POF4), 2520826 (BW-POF4), 2330199 (TTPA-S) and 2385206 (SiW-POF2(0.3)-S(60%)) and 2520622 (SiW-POF2(0.3)-S(60%)-RH85). These data can be obtained free of charge from CCDC via https://www.ccdc.cam.ac.uk/structures/. Source data are provided with this paper.
