# Supplementary material for: Cation-directed assembly and sequential functionalization enable superprotonic polyanion–organic frameworks for high-power fuel cells
Source: Nat Chem. 2026 Jun 1;18(7):1231–41. doi: 10.1038/s41557-026-02169-8 (PMC13323076; doi:10.1038/s41557-026-02169-8)
Supplement: Supplementary file 1 — Supplementary Figs. 1–128, Discussion (Notes 1–8) and Tables 1–27. [file 41557_2026_2169_MOESM1_ESM.pdf]

nature chemistry

Supplementary information

https://doi.org/10.1038/s41557-026-02169-8

Cation-directed assembly and sequential functionalization enable superprotonic polyanion–organic frameworks for high-power fuel cells

In the format provided by the authors and unedited

SUPPLEMENTARY INFORMATION

Table of contents

1. Sythetic discussion and structural characterization of POFs..2

2. Proton Conductivity Experiment of POFs 37

3. Synthesis and structural characterization of TTPA-S and SiW-POF-S.................................... 43

4. Proton Conductivity Experiment of POF-S 57

5. Gas and water vapor adsorption and dye uptake in solution 62

6. Solid State NMR 69

7. Conductivity (σ) values of the as-synthesized compounds... 70

8. Fabrication and structural characterization of SiW-POF2-S(60%)@Nafion hybrid membranes 71

9. Fuel cell details 83

10. Computational methods 85

11. References 92

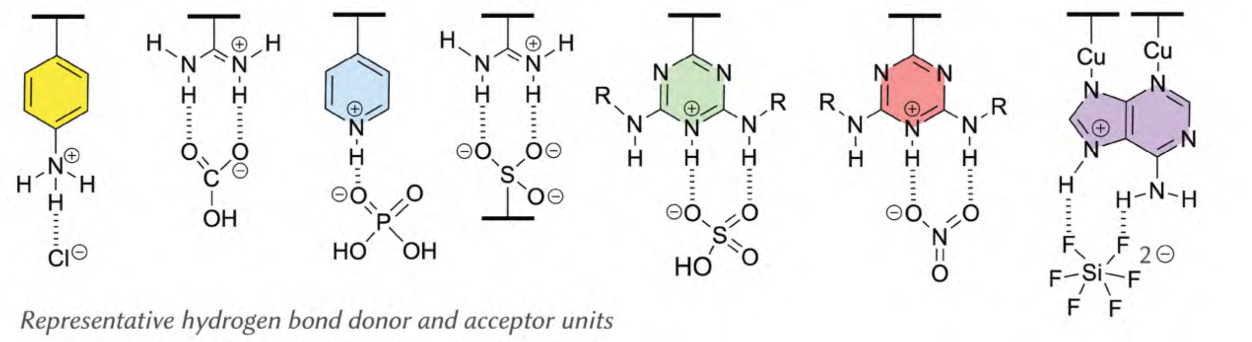

Supplementary Fig. 1: Schematic demonstration of the representative acid and base hydrogen bond donor and acceptor units employed for charged-assisted hydrogen-bonded organic frameworks.

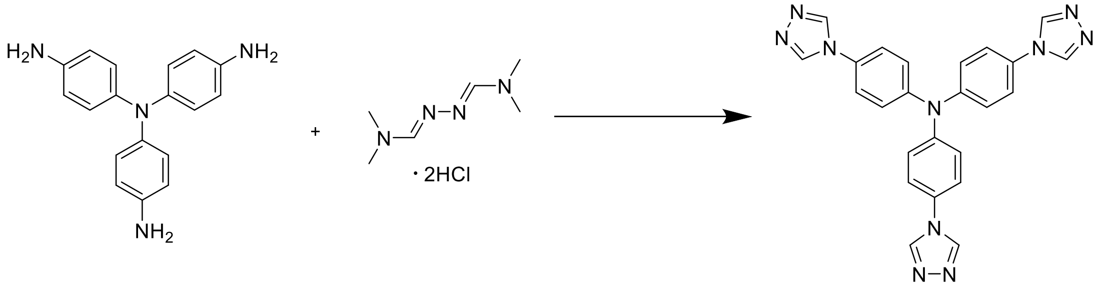

TAPA **TTPA**

Supplementary Fig. 2: Synthetic procedure for TTPA.

1. Synthetic discussion and structural characterization of POFs

Supplementary Note 1. Effect of symmetry, V-shaped pocket and arm length of organic tecton on the formation of porous POFs.

To delineate structural parameters governing the formation of porous POFs, we performed extensive synthetic efforts using various tectons to assemble with Kegin-type POM clusters ({SiW_{12}}, {PW_{12}}, {BW_{12}}) to probe the critical factors governing the formation of porous POFs. Initially, we attempted to synthesize C_3 symmetrical tectons with both longer (T1) and shorter (T2) arms (**Supplementary Fig. 3**); however, the T2 tecton proved difficult to isolate due to the synthetic challenges of grafting three triazole moieties onto the central benzene ring. Consequently, POF synthesis was pursued solely with T1, which yielded crystalline solids rather than high-quality crystals. We propose that the extended length of T1, relative to TTPA, induces rapid assembly and precipitation, which precludes the elucidation of the relation between arm length and porosity of POFs due to the lack of crystal structures. To provide further rationale, simulations were conducted with Materials Studio using the crystal structure of SiW-POF2 as a starting model

(please see below for details). As illustrated in **Supplementary Fig. 5**, the simulated packing structures indicate that the longer T1 tecton tightly encapsulates the $\{SiW_{12}\}$ cluster within its V-shaped pockets, resulting in a nearly non-porous packing arrangement along the c-axis. Conversely, the shorter arms of T2 anchor the $\{SiW_{12}\}$ cluster in a more relaxed manner, facilitating the formation of hexagonal channels with larger dimensions. This suggests that an optimal, "size-matched" arm length is crucial for generating porous architectures.

To evaluate the influence of symmetry/topology, we tested the assembly of POM clusters with T_d and pseudo-C_4 symmetric tectons (T3 and T4) also featuring V-shaped pockets (**Supplementary Fig. 3**). While T3 yielded only precipitates, T4 produced well-defined crystals of BW-POF4; however, this structure adopts a dense packing mode with no detectable porosity despite the encapsulation of $\{BW_{12}\}$ by the V-shaped pockets of T4 (**Supplementary Fig. 4**). Materials Studio simulations revealed that while the V-shaped pockets of T3 can effectively encapsulate $\{SiW_{12}\}$, they still yield non-porous supramolecular architectures (**Supplementary Fig. 5**). This demonstrates that the symmetry of tecton also dictates the assembly pathway. Finally, we employed a linear C_2 symmetric T5 which lacks V-shaped pocket to assemble with $\{SiW_{12}\}$ to afford SiW-POF4. As shown in **Supplementary Fig. 4**, the absence of V-shaped pockets prevents T5 from efficiently wrapping the $\{SiW_{12}\}$ cluster; instead, the tectons distribute around $\{SiW_{12}\}$ cluster tightly and pack on each other seamlessly via π-π stacking, leading to a supramolecular structure devoid of open channels (**Supplementary Fig. 4**). This manifests that V-shaped pockets are indispensable for the construction of porous frameworks.

In conclusion, we demonstrate that both the trigonal topology of the tecton and the V-shaped geometry of the pocket are essential for porosity formation (**Supplementary Table 1**). Furthermore, the size match between V-shaped arms and POM clusters also plays an important role (**Supplementary Table 1**). We thus propose that the synergistic effect involving the trigonal topology, V-shaped pocket geometry and size match governs the successful assembly of porous POFs described in this study.

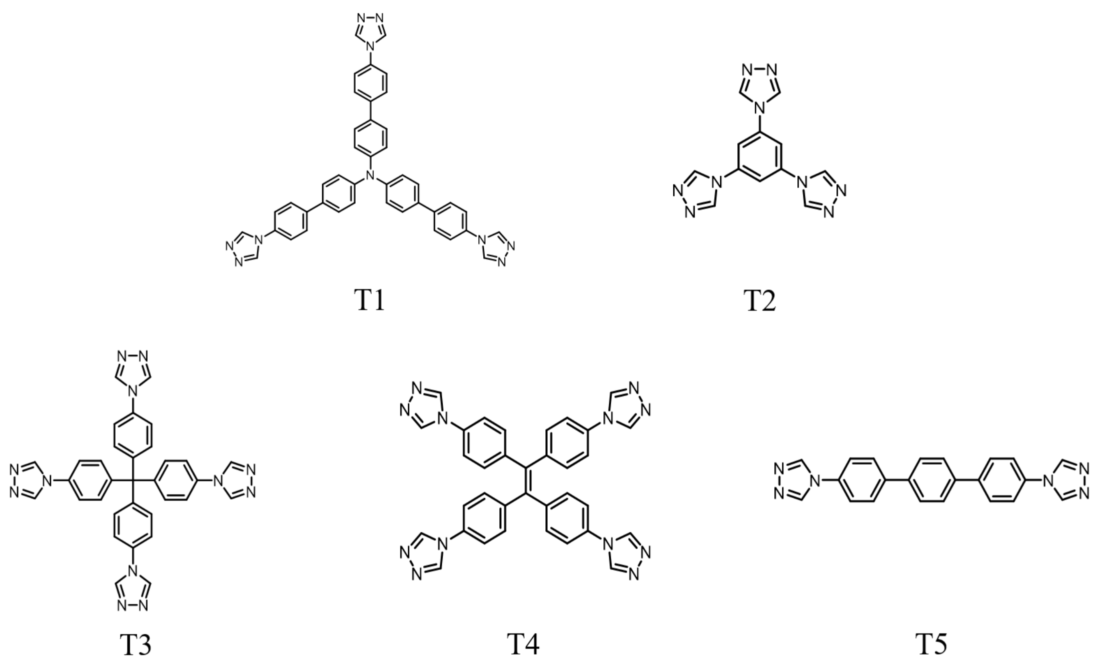

Supplementary Fig. 3: Schematic representation of tectons employed for controlled synthesis of POFs.

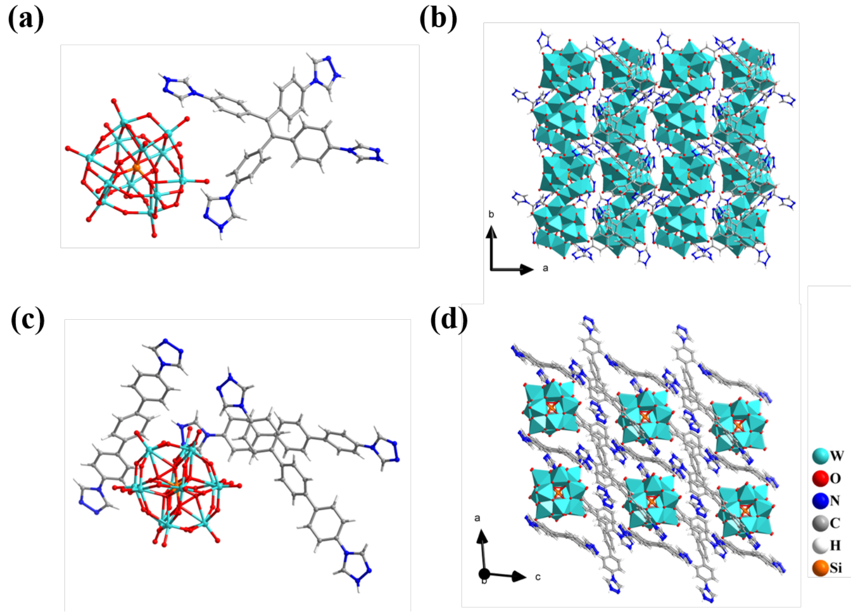

Supplementary Fig. 4: (a) The asymmetric unit of BW-POF4; (b) structural stacking of BW-POF4 along *c* axis; (c) the asymmetric unit of SiW-POF4; (d) structural stacking of SiW-POF4 along *b* axis.

Supplementary Note 2. Simulation details for the construction of POFs from T1 to T3

All structure construction and geometry optimization steps were performed using the Materials Studio software package.[1] Structural models incorporating linkers of different lengths were generated using the crystal structure of SiWPOF2 as the parent framework. In this process, the TTPA linker was replaced by the target linker, while maintaining the same center of mass and three-dimensional orientation as the original TTPA unit. The resulting crystal structures were subsequently subjected to geometry optimization using the Forcite module with the universal force field (UFF).[2] During optimization, the cell angles were held fixed and only the cell lengths were allowed to vary, based on the assumption that the new three-armed linkers preserve the original network topology. For the structure incorporating a four-armed linker, all unit-cell parameters were allowed to relax. The iRASPA software package was employed to calculate and compare the helium void fractions (porosity) of the generated structural models.[3]

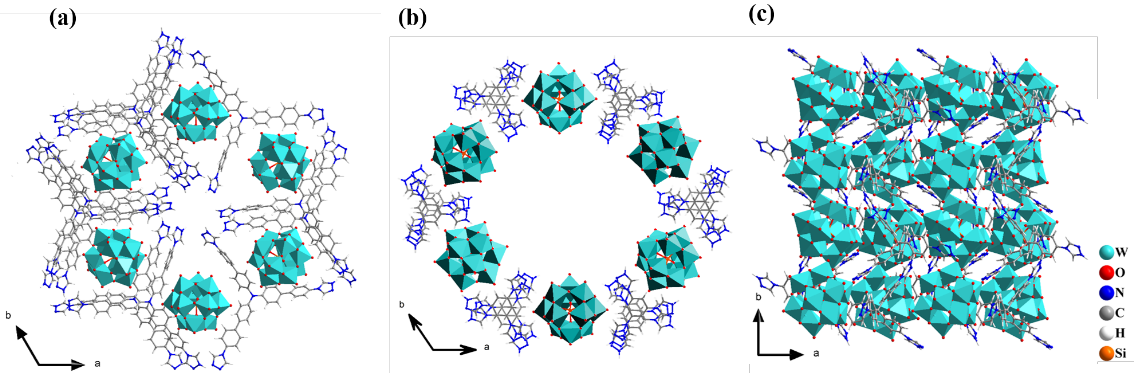

Supplementary Fig. 5: View of the packing supramolecular structures of simulated POFs constructed from T1 (a), T2 (b) and T3 (c), respectively.

Supplementary Table 1: Summary of the influence of symmetry, V-shaped pocket and arm length of tecton on the formation of POF structures

Tecton	Symmetry/topology	V-shaped pocket	Arm length	POFs
TTPA	trigonal	Yes	proper*	porous
T1	trigonal	Yes	longer	nonporous#
T2	trigonal	Yes	shorter	porous#
T3	T_d	Yes	proper	nonporous#
T4	pseudo-C_4	Yes	proper	nonporous
T5	C_2	No	N/A	nonporous

* The standard of “proper” is made by the length of one phenyl ring plus one triazole; # Based on simulation using Materials Studio.

Supplementary Note 3. The template effect of imidazole and derivatives

During our investigation, we systematically explored various imidazole derivatives and other N-heterocyclic compounds to determine their efficacy as templates during the self-assembly process (**Supplementary Fig. 6**). So far, only 2-methylimidazole has demonstrated a similar templating effect (**Supplementary Fig. 7**), while other imidazole derivatives failed to induce the formation of the POF architecture. Specifically, even slight modifications to the substituents—such as the introduction of amino, nitro, or cyano groups at the 2- or 3-positions—consistently resulted in precipitation. Furthermore, larger molecules like benzimidazole yielded only unidentified solids. This indicates the substituents and size of imidazole are critical for the generation of POFs. In view of other heterocycles, we specifically selected pyrazole, triazole, and tetrazole due to their comparable molecular sizes to imidazole. However, these attempts yielded only amorphous solids rather than crystalline products. We proposed that the different pKb and arrangement of N sites of these heterocycles have great influence on the assembly as the protonation rate and hydrogen bond pattern are critical for directing the construction of POFs. Overall, we think imidazole is the optimal template regarding the proper ability to accept proton, well-matched size and N sites to establish hydrogen bonds.

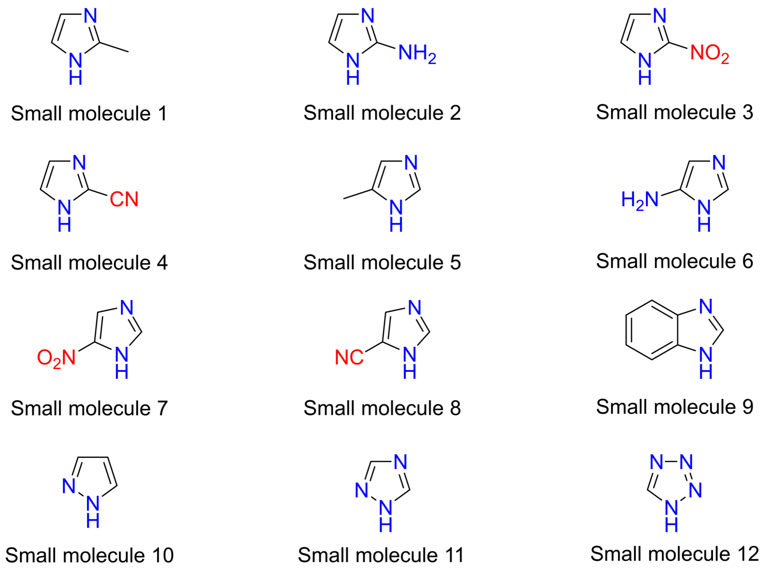

Supplementary Fig. 6: Schematic representation of N-heterocycles employed for synthesis of SiW-POF2 series.

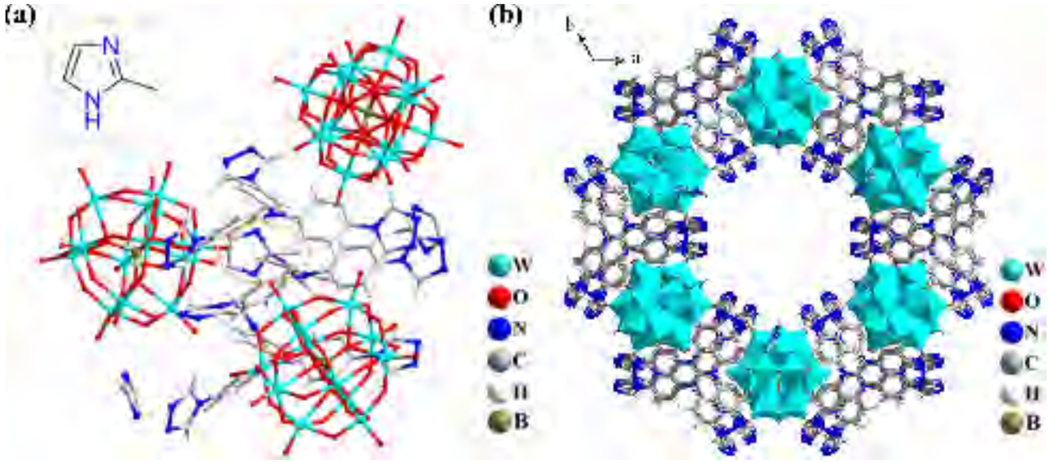

Supplementary Fig. 7: (a) The asymmetric unit (a) and structural stacking (b) of 2-methyl imidazole derivative of SiW-POF2(0.3).

X-ray crystallography. Single-crystal X-ray diffraction data of the SiW-POF1, BW-POF1, SiW-POF2(0.3), BW-POF2(0.3), BW-POF3 were obtained on a Bruker D8 VENTURE Apex IV single-crystal x-ray diffractometer with Mo/Cu Kα radiation at 150 K or Ga Kα radiation at 150 K. The empirical absorption correction was based on equivalent reflections. Data collection and reduction were performed using the Apex 4 software package and structure solution, and refinement was carried out by SHELXS-2014[4] and SHELXL-2018[5] using Olex2.[6] Solvent water molecules with reasonable Ueq are located from residual electron density map, while the relatively weak Q peaks are squeezed by applying 'Solvent mask' embedded in Olex-2.[7] Crystallographic formulas typically contain many more water molecules in the crystal lattice than found in the sample after drying. Overall, the final refinement statistics are good, and in all cases the structural analysis allows us to unambiguously determine the structures of the compounds (**Supplementary Table 1** and **2**). Non-hydrogen atoms were refined anisotropically during the final cycles. All hydrogen atoms of the organic molecule were placed based on geometrical considerations and were included in the structure factor calculation. Crystallographic data have been deposited at the Cambridge Crystallographic Data Centre with CCDC numbers: 2330203 (SiW-POF1), 2330204 (BW-POF1), 2330202 (SiW-POF2(0.3)), 2385205 (PW-POF2(0.3)), 2330200 (BW-POF2(0.3)), 2519967 (SiW-POF3), 2519966 (PW-POF3), 2330201 (BW-POF3), 2520631 (SiW-POF4), 2520826 (BW-POF4), 2330199 (TTPA-S) and 2385206 (SiW-POF2(0.3)-S(60%)), 2520622 (SiW-POF2(0.3)-S(60%)-RH85).

1.1.Crystallographic data and crystal structures of POFs

Supplementary Table 2: Crystallographic data and structure refinement

Compound	SiW-POF1	BW-POF1	SiW-POF2(0.3)
formula	$C_{108}H_{96}N_{45}Si_{1.5}W_{18}O_{64.5}$	$C_{48}H_{53}N_{20}BW_{12}O_{46}$	$C_{51.6}H_{44.8}N_{24}SiW_{12}O_{40}$
Formula weight	6407.73	3863.11	3852.99
Crystal system	Monoclinic	Trigonal	Trigonal
Space group	*C2*/m	*R-3m*	*R-3m*
a /Å	29.804(2)	46.953(9)	46.957(9)
b /Å	45.592(3)	46.953(9)	46.957(9)
c /Å	32.448(2)	13.158(3)	13.210(3)
α/°	90	90.00(3)	90.00
β/°	119.515(2)	90.00(3)	90.00
γ/°	90	120.00(3)	120.00
V /Å^3	38370(5)	25122(11)	25225(11)
Z	8	9	9
ρ calc /g·cm^{-3}	2.218	2.298	2.283
R(int)	0.1011	0.0748	0.0603
μ/mm^{-1}	10.832	15.657	15.648
F(000)	23424	15678	15599
R_1/wR_2 (I>2σ(I))[a]	0.0601/0.1438	0.0562/0.1550	0.0780/0.2163
R_1/wR_2 (all data)	0.1138/0.1438	0.0566/0.1554	0.0855/0.2249
GooF (all data)[b]	1.024	1.060	1.080
Data completeness	99.5 %	99.9 %	100.0 %

[a] $R_1 = \sum||Fo|-|Fc||/\sum|Fo|$; $wR_2 = \{\sum w[(Fo)^2-(Fc)^2]^2/\sum w[(Fo)^2]^2\}^{1/2}$

[b] $GooF = \{\sum w[(Fo)^2-(Fc)^2]^2/(n-p)\}^{1/2}$

Supplementary Table 3: Crystallographic data and structure refinement

Compound	PW-POF2(0.3)	BW-POF2(0.3)	BW-POF3
formula	$C_{51.6}H_{43.8}N_{22.4}PW_{12}O_{40}$	$C_{51.6}H_{45.8}N_{22.4}BW_{12}O_{40}$	$C_{72}H_{61}N_{30}BW_{12}O_{41}$
Formula weight	3854.86	3836.71	4219.51
Crystal system	Trigonal	Trigonal	Triclinic
Space group	*R-3m*	*R-3m*	*P-1*
a /Å	47.426(9)	47.202(9)	15.1004(5)
b /Å	47.426 (9)	47.202(9)	15.8573(4)
c /Å	13.065(3)	12.985(3)	23.8967(8)
α/°	90(3)	90(3)	85.3530(10)
β/°	90(3)	90(3)	71.4340(10)
γ/°	120(3)	120(3)	63.4930(10)
V /$Å^3$	25450(11)	25056(11)	4841.5(3)
Z	9	9	2
ρ calc /$g\cdot cm^{-3}$	2.264	2.288	2.894
R(int)	0.0654	0.0745	0.0976
μ(MoKα) /mm^{-1}	12.235	15.673	14.293
F(000)	15599	15527	3848
R_1/wR_2 (I>2σ(I))[a]	0.0636/0.1870	0.0740/0.2277	0.0957/0.2143
R_1/wR_2 (all data)	0.0797/0.2066	0.0771/0.2300	0.1096/0.2204
GooF (all data)[b]	1.064	1.053	1.247
Data completeness	99.6 %	99.9 %	99.8 %

[a]$R_1 = \sum||Fo|-|Fc||/\sum|Fo|$; $wR_2 = \{\sum w[(Fo)^2-(Fc)^2]^2/\sum w[(Fo)^2]^2\}^{1/2}$

[b]$GooF = \{\sum w[(Fo)^2-(Fc)^2]^2/(n-p)\}^{1/2}$

Supplementary Table 4: Crystallographic data and structure refinement

Compound	SiW-POF3	PW-POF3
formula	$C_{48}H_{40}N_{20}SiW_{12}O_{40}$	$C_{48}H_{39}N_{20}PW_{12}O_{40}$
Formula weight	3899.17	3773.04
Crystal system	Triclinic	Triclinic
Space group	*P -1*	*P -1*
a /Å	10.605(4)	10.8292(11)
b /Å	10.605(4)	12.5455(12)
c /Å	15.483(11)	14.9511(15)
α/°	94.75(2)	71.482(4)
β/°	106.32(2)	72.724(4)
γ/°	90.405(11)	83.841(4)
V /$Å^3$	2022.8(18)	1839.0(3)
Z	1	1
ρ calc /g·cm^{-3}	3.201	3.407
R(int)	0.1144	0.1186
μ(MoKα) /mm^{-1}	17.107	18.808
F(000)	1754.0	1690.0
R_1/wR_2 (I>2σ(I))[a]	0.0645/0.1725	0.0667/0.1324
R_1/wR_2 (all data)	0.0770/0.1907	0.0938/0.1442
GooF (all data)[b]	1.062	1.030
Data completeness	99.8 %	99.6 %

[a]$R_1 = \sum||Fo|-|Fc||/\sum|Fo|$; $wR_2 = \{\sum w[(Fo)^2-(Fc)^2]^2/\sum w[(Fo)^2]^2\}^{1/2}$

[b]$GooF = \{\sum w[(Fo)^2-(Fc)^2]^2/(n-p)\}^{1/2}$

Supplementary Table 5: Hydrogen bond lengths (Å) and angles (º) for **SiW-POF1**.

D-H···A	d(D-H)	d(H···A)	d(D···A)	∠DHA
C(99)–H(99)···O(13)	0.95	2.68	3.553	153.3
C(97)–H(97)···O(13)	0.95	2.41	3.345	168.2
C(85)–H(85)···O(13)	0.95	2.36	3.252	156.4
C(40)–H(40)···O(8)	0.95	2.36	3.163	142.2
C(38)–H(38)···O(20)	0.95	2.49	3.383	156.5
C(37)–H(37)···O(19)	0.95	2.42	3.295	152.9
C(23)–H(23)···O(20)	0.95	2.60	3.356	137.1
C(2)–H(2)···O(9)	0.95	2.54	3.332	141.4
C(14)–H(14)···O(16)	0.95	2.21	3.154	173.6
C(13)–H(13)···O(5)	0.95	2.64	3.308	128.1
C(13)–H(13)···O(7)	0.95	2.51	3.426	163.0
C(108)–H(108)···O(22)	0.95	2.51	3.309	141.4
C(103)–H(103)···O(16)	0.95	2.44	3.382	174.9
C(95)–H(95)···O(66)	0.95	2.01	2.878	151.0
C(95)–H(95)···O(68)	0.95	2.51	3.225	132.6
C(82)–H(82)···O(60)	0.95	2.37	3.002	123.6
C(72)–H(72)···O(68)	0.95	2.47	3.078	122.0
C(63)–H(63)···O(68)	0.95	2.38	3.315	168.2
C(58)–H(58)··· O(60)	0.95	2.34	2.978	124.1
C(50)–H(50)···O(61)	0.95	2.64	3.449	143.5
C(49)–H(49)···O(64)	0.95	2.53	3.289	136.7
C(35)–H(35)···O(65)	0.95	2.69	3.448	136.7
C(26)–H(26A)···O(59)	0.95	2.22	3.146	164.5
C(26)–H(26A)···O(67)	0.95	2.48	3.179	130.5
C(10)–H(10)···O(57)	0.95	2.68	3.311	124.8
C(109)–H(109)···O(44)	0.95	2.51	3.319	143.8
C(25)–H(25)···O(29)	0.95	2.62	3.423	142.0
C(45)–H(45)···O(37)	0.95	2.40	3.303	158.3
C(45)–H(45)···O(38)	0.95	2.64	3.317	129.0
C(7)–H(7)···O(42)	0.95	2.38	3.322	173.2

N(16)–H(16)···O(71)	0.88	2.19	2.936	141.9
N(18)–H(18)···O(72)	0.88	1.88	2.592	136.9
N(28)–H(28)···O(73)	0.88	1.90	2.678	147.4
N(12)–H(12)···O(70)	0.88	2.05	2.899	161.8
N(26)–H(26)···O(70)	0.88	2.15	3.006	162.8
N(21)–H(21)···O(69)	0.88	2.05	2.806	143.6
N(12)–H(12)···O(63)	0.88	2.67	3.208	120.4

Supplementary Table 6: Hydrogen bond lengths (Å) and angles (º) for **BW-POF1**.

D-H···A	d(D-H)	d(H···A)	d(D···A)	∠DHA
C(1)–H(1A)···O(12)	0.95	2.11	2.962	147.9
C(5)–H(5)···O(7)	0.95	2.52	3.401	155.3
C(7)–H(7)···O(10)	0.95	2.43	3.376	176.7
C(8)–H(8)···O(12)	0.95	2.56	3.296	134.2
C(13)–H(13)···O(5)	0.95	2.59	3.357	137.7
C(13)–H(13)···O(8)	0.95	2.62	3.460	147.4
N(1)–H(1)···O(15)	0.88	1.92	2.778	164.4
O(16)–H(16A)···O(9)	0.932	2.66	3.204	117.7
O(16)–H(16A)···O(14)	0.932	1.99	2.875	159.1
O(17)–H(17B)···O(4)	0.919	1.99	2.869	160.8

Supplementary Table 7: Hydrogen bond lengths (Å) and angles (º) for **SiW-POF2(0.3)**.

D-H···A	d(D-H)	d(H···A)	d(D···A)	∠DHA
C(2)–H(2)···O(10)	0.95	2.10	3.046	172.4
C(4)–H(4)···O(10)	0.95	2.54	3.287	135.6
C(5)–H(5)···O(9)	0.95	2.44	3.394	178.5
C(7)–H(7)···O(5)	0.95	2.44	3.330	155.8
C(13)–H(13)···O(6)	0.95	2.62	3.387	137.9
C(13)–H(13)···O(7)	0.95	2.58	3.422	148.6
N(8)–H(8A)···O(11)	0.88	2.54	3.311	146.3
N(8)–H(8A)···O(12)	0.88	2.66	3.231	123.7

Supplementary Table 8: Hydrogen bond lengths (Å) and angles (º) for **PW-POF2(0.3)**.

D-H···A	d(D-H)	d(H···A)	d(D···A)	∠DHA
C(2)–H(2)···O(7)	0.95	2.12	3.070	174.3
C(8)–H(8)···O(7)	0.95	2.62	3.376	136.8
C(5)–H(5)···O(13)	0.95	2.46	3.343	155.2
C(7)–H(7)···O(10)	0.95	2.41	3.361	178.9
C(13)–H(13)···O(2)	0.95	2.55	3.348	142.3
C(13)–H(13)···O(11)	0.95	2.54	3.396	150.9
N(7)–H(7A)···O(6)	0.88	2.51	3.092	124.3
N(7)–H(7A)···O(12)	0.88	2.40	3.218	155.1
N(9)–H(9)···O(2)	0.88	2.69	3.540	162.1

Supplementary Table 9: Hydrogen bond lengths (Å) and angles (º) for **BW-POF2(0.3)**.

D-H···A	d(D-H)	d(H···A)	d(D···A)	∠DHA
C(2)–H(2A)···O(8)	0.95	2.12	3.057	169.2
C(4)–H(4)···O(8)	0.95	2.63	3.378	136.5
C(5)–H(5)···O(4)	0.95	2.40	3.351	178.0
C(7)–H(7)···O(12)	0.95	2.42	3.301	153.4
C(13)–H(13)···O(5)	0.95	2.56	3.420	151.3
C(13)–H(13)···O(13)	0.95	2.64	3.424	140.6
N(7)–H(7A)···O(9)	0.875	2.40	3.032	129.4
N(7)–H(7A)···O(10)	0.875	2.51	3.180	134.2
N(7)–H(7A)···O(11)	0.875	2.58	3.275	136.4
N(8)–H(8A)···O(13)	0.88	2.61	3.455	160.4

Supplementary Table 10: Hydrogen bond lengths (Å) and angles (º) for **BW-POF3**.

D-H···A	d(D-H)	d(H···A)	d(D···A)	∠DHA
C(13)–H(13)···O(17)	0.95	2.55	3.244	130.3
C(14)–H(14)···O(22)	0.95	2.45	3.185	134.1
C(18)–H(18)···O(14)	0.95	2.66	3.534	154.2
C(25)–H(25)···O(45)	0.95	2.44	3.332	155.6
C(32)–H(32)···O(5)	0.95	2.53	3.340	143.6

C(47)–H(47)···O(10)	0.95	2.35	3.207	150.7
C(48)–H(48)···O(11)	0.95	2.64	3.380	135.3
C(48)–H(48)···O(18)	0.95	2.45	3.254	141.9
C(61)–H(61)···O(21)	0.95	2.67	3.593	165.1
C(62)–H(62)···O(45)	0.95	2.55	3.423	152.4
C(64)–H(64)···O(14)	0.95	2.30	3.236	168.1
C(72)–H(72)···O(22)	0.95	2.16	2.916	135.4
C(10)–H(10)···O(32)	0.95	2.67	3.391	132.7
C(11)–H(11A)···O(37)	0.95	2.53	3.312	139.8
C(21)–H(21)···O(27)	0.95	2.42	3.352	167.1
C(35)–H(35)···O(37)	0.95	2.69	3.480	141.4
C(49)–H(49)···O(41)	0.95	2.39	3.280	156.3
C(50)–H(50)···O(31)	0.95	2.24	2.983	134.6
C(52)–H(52)···O(41)	0.95	2.26	3.072	143.0
C(55)–H(55)···O(28)	0.95	2.58	3.244	126.9
C(69)–H(69)···O(38)	0.95	2.69	3.555	152.3
C(40)–H(40)···N(6)	0.95	2.09	2.818	132.9
C(15)–H(15)···N(26)	0.95	2.41	3.308	158.2
N(22)–H(22)···O(14)	0.88	1.85	2.717	169.9
N(29)–H(29)···N(27)	0.88	1.83	2.607	146.2
N(17)–H(17)···N(12)	0.88	1.89	2.698	152.1
N(11)–H(17)···N(20)	0.88	1.69	2.446	143.0
O(45)–H(45A)···O(6)	0.87	2.57	3.313	144.0
O(45)–H(45A)···O(13)	0.87	2.59	3.344	146.3

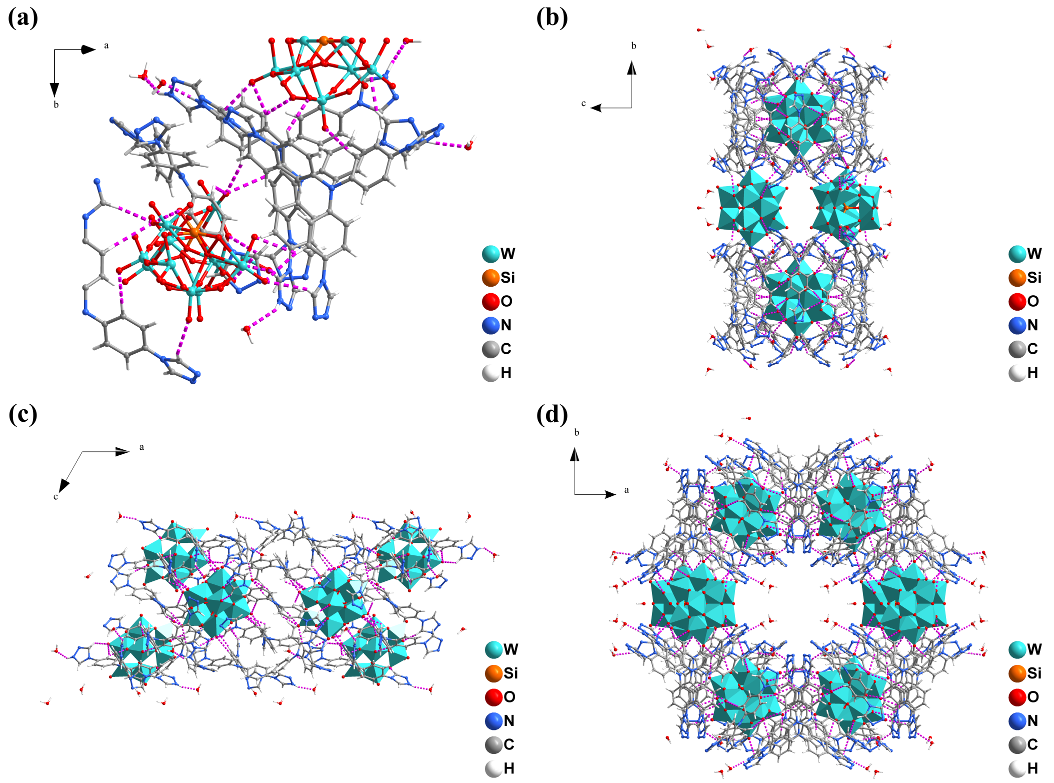

Supplementary Fig. 8: (a) The asymmetric unit of SiW-POF1; (b) structural stacking of SiW-POF1 viewed in the direction of *a* axis; (c) *b* axis; (d) *c* axis.

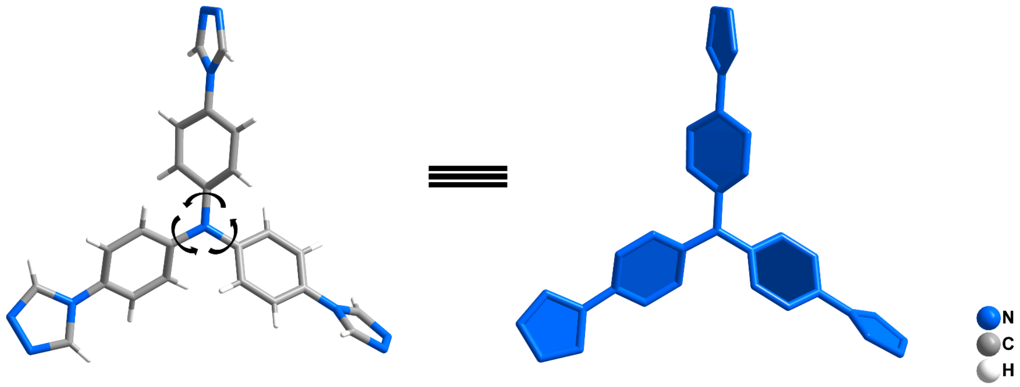

Supplementary Fig. 9: Propeller-like TTPA structure.

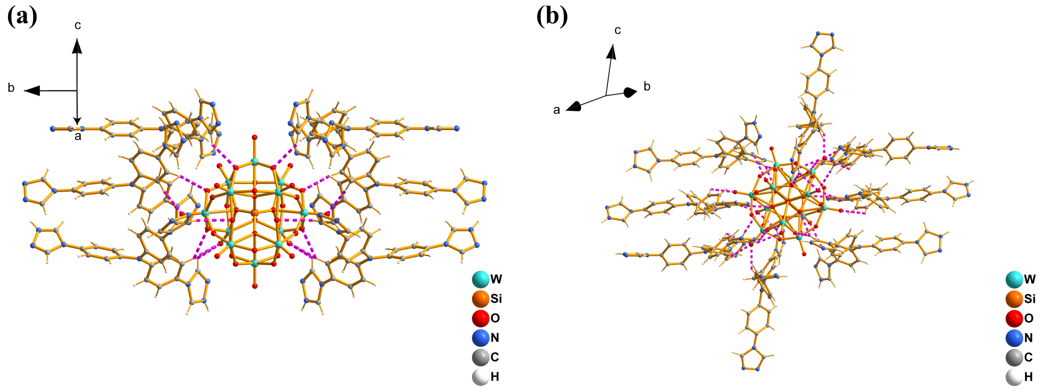

Supplementary Fig. 10: The hydrogen bond connection between $\{SiW_{12}\}$ and TTPA.

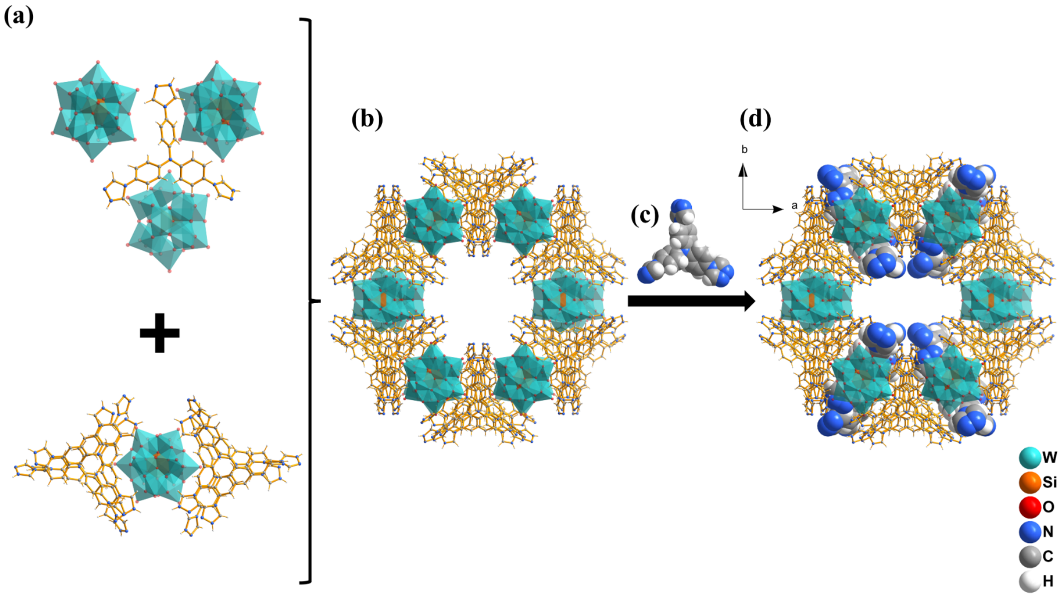

Supplementary Fig. 11: The construction of two types of hydrogen bonds in SiW-POF1 (a) the combination of the first type of TTPA and $\{SiW_{12}\}$; (b) porous framework composed of the first type of TTPA and $\{SiW_{12}\}$; (c) the space-filling model of the second type of TTPA; (d) irregular hexagonal 1D channel in SiW-POF1.

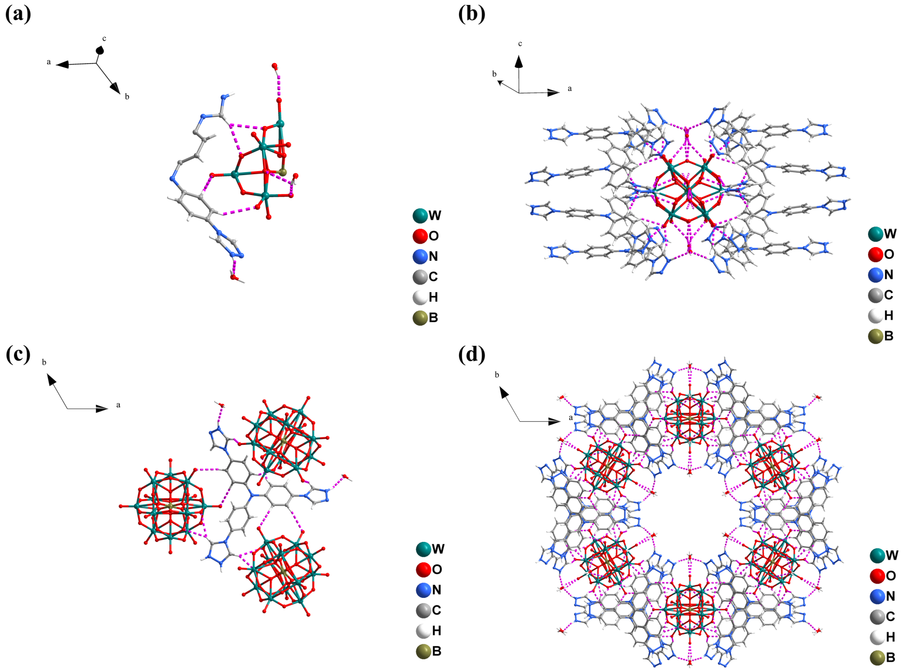

Supplementary Fig. 12: (a)The asymmetric unit of BW-POF1; (b) the hydrogen bond connection between one {BW_{12}} and the surrounding eight TTPA ligands; (c) the hydrogen bond connection between one TTPA ligand and three surrounding BW_{12}; (d) hexagonal 1D channel in BW-POF1.

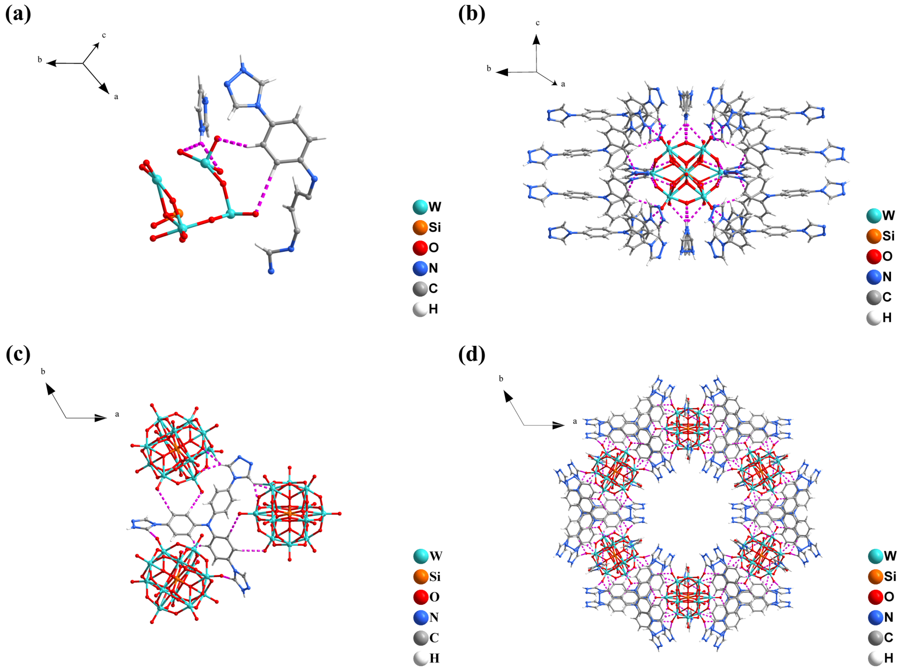

Supplementary Fig. 13: (a) The asymmetric unit of SiW-POF2(0.3); (b) the hydrogen bonding between one {SiW_{12}} and the surrounding eight TTPA ligands and two imidazoles; (c) the hydrogen bond connection between one TTPA ligand and three surrounding {SiW_{12}}; (d) hexagonal 1D channel in SiW-POF2(0.3).

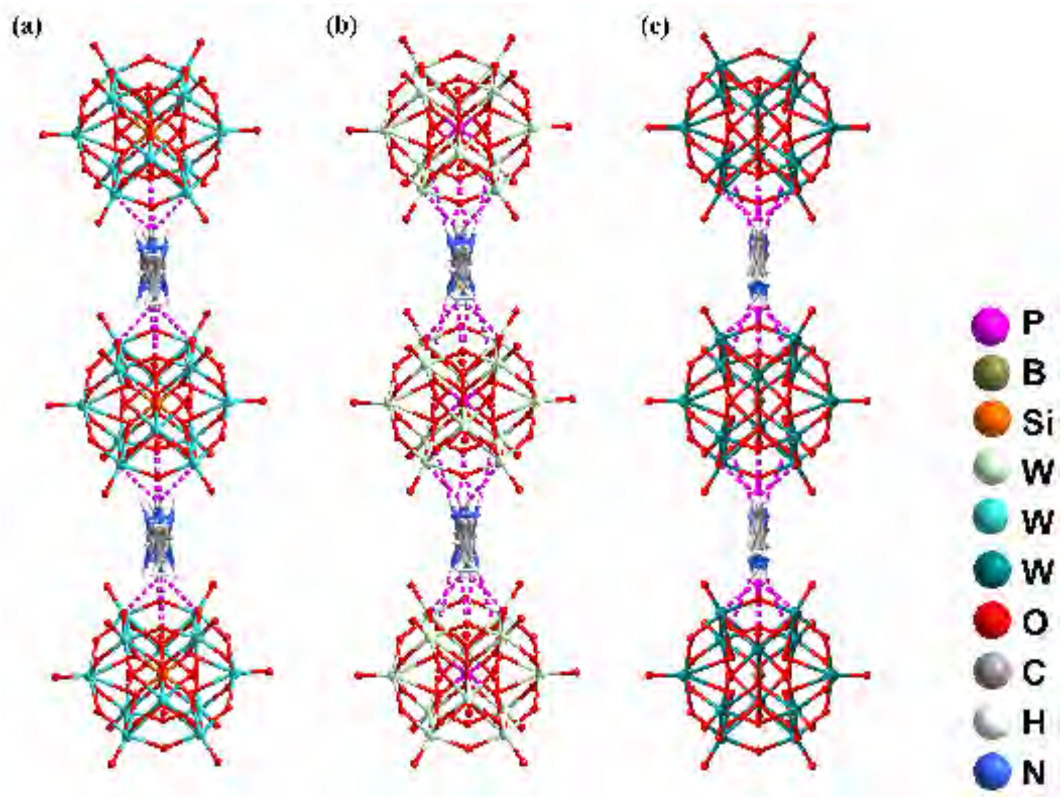

Supplementary Fig. 14: (a) Hydrogen bonding between IM and $\{SiW_{12}\}$ in SiW-POF2(0.3); (b) hydrogen bonding between IM and PW_{12} in PW-POF2; (c) hydrogen bonding between IM and BW_{12} in BW-POF2(0.3).

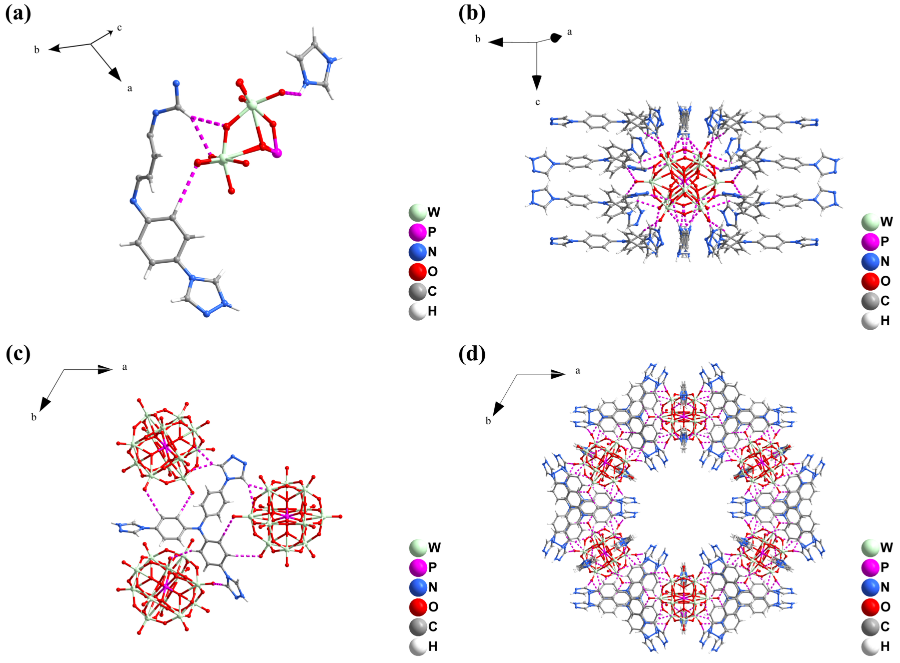

Supplementary Fig. 15: (a) Hydrogen bonding between IM and $\{PW_{12}\}$ in PW-POF2(0.3); (b) hydrogen bonding between IM and PW_{12} in PW-POF2(0.3).

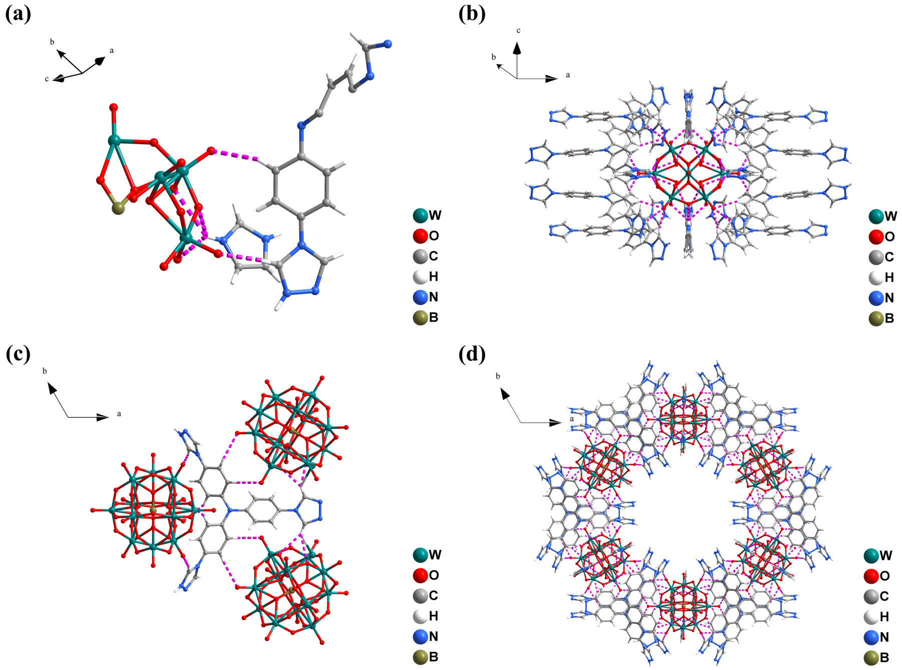

Supplementary Fig. 16: (a)The asymmetric unit of BW-POF2(0.3); (b) the hydrogen bonding between one {BW_{12}} and the surrounding eight TTPA ligands and two imidazoles; (c) the hydrogen bond connection between one TTPA ligand and three surrounding {BW_{12}}; (d) hexagonal 1D channel in BW-POF2(0.3).

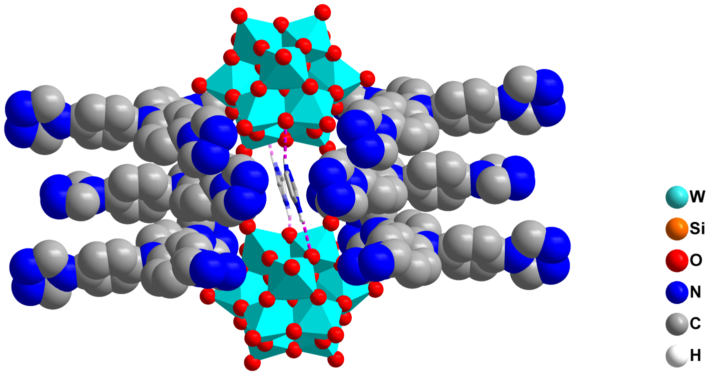

Supplementary Fig. 17: View of the confinement of protonated imidazolium within a cage-like apartment constructed from two {SiW_{12}} clusters and six TTPA in the crystal structure of SiW-POF2(0.3). The hydrogen bonds between protonated imidazolium and {SiW_{12}} cluster are highlighted by dotted green lines.

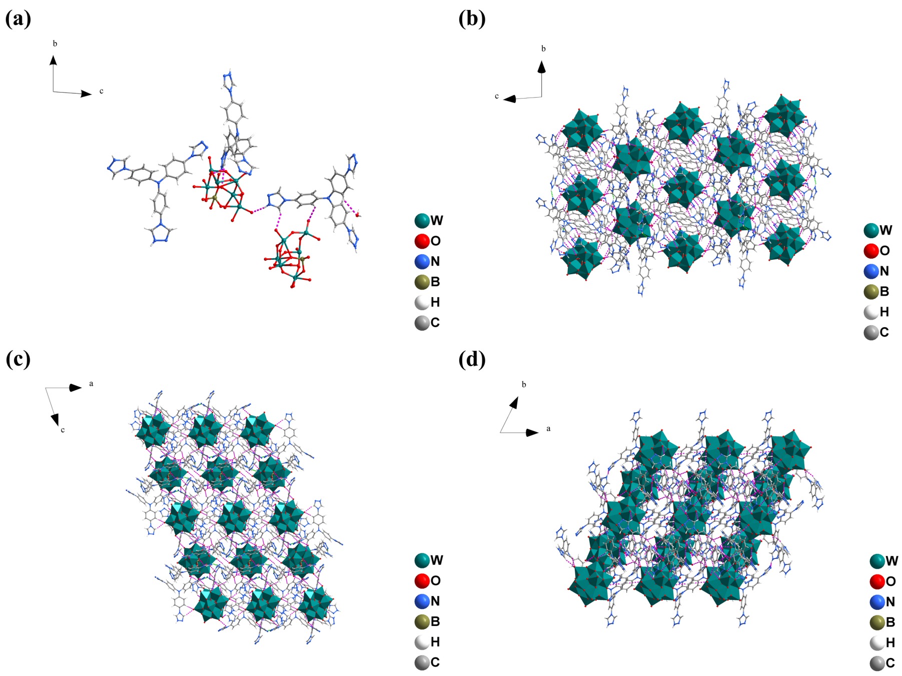

Supplementary Fig. 18: (a)The asymmetric unit of BW-POF3; (b) structural stacking of BW-POF3 viewed in the direction of a axis; (c)b axis; (d)c axis.

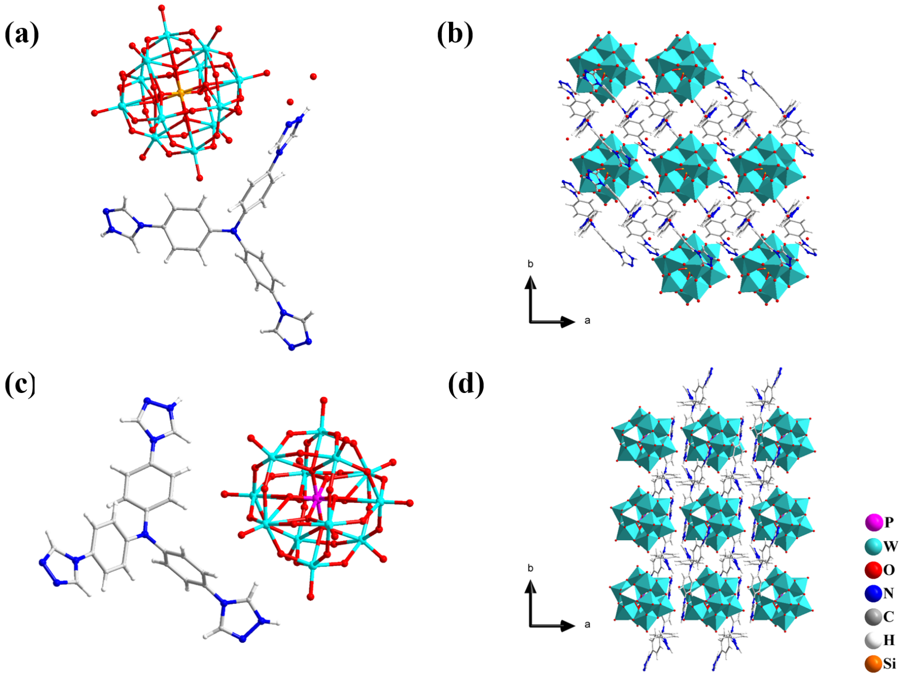

Supplementary Fig. 19: (a) The asymmetric unit of SiW-POF3; (b) Structural stacking of SiW-POF3 along *c* axis; (c) The asymmetric unit of PW-POF3; (d) Structural stacking of PW-POF3 along *c* axis.

1.2. Characterization of POFs

Here we provide the complete PXRD, FTIR and TGA characterisation of the POF series that we synthesized.

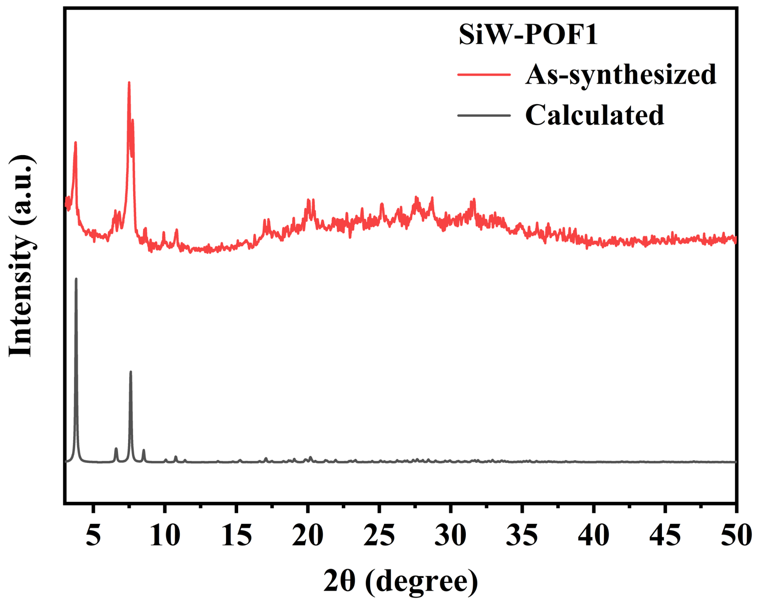

Supplementary Fig. 20: The PXRD pattern of SiW-POF1.

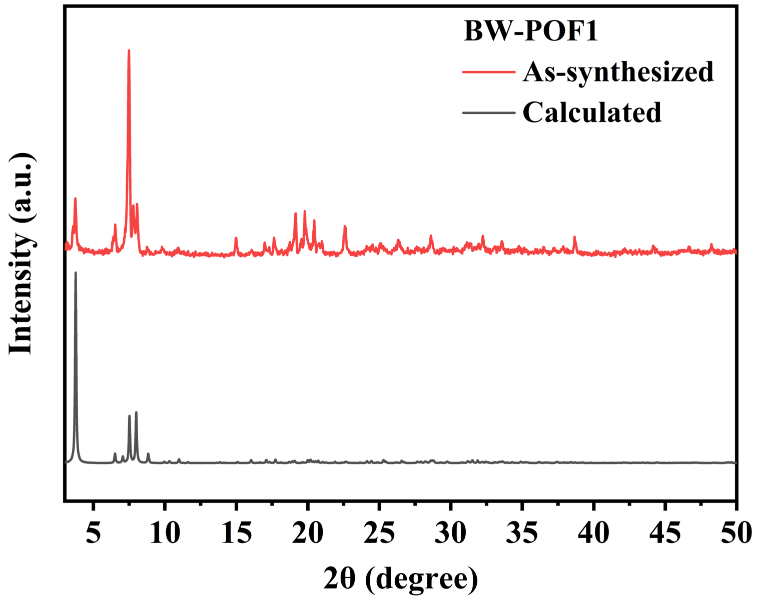

Supplementary Fig. 21: The PXRD pattern of BW-POF1.

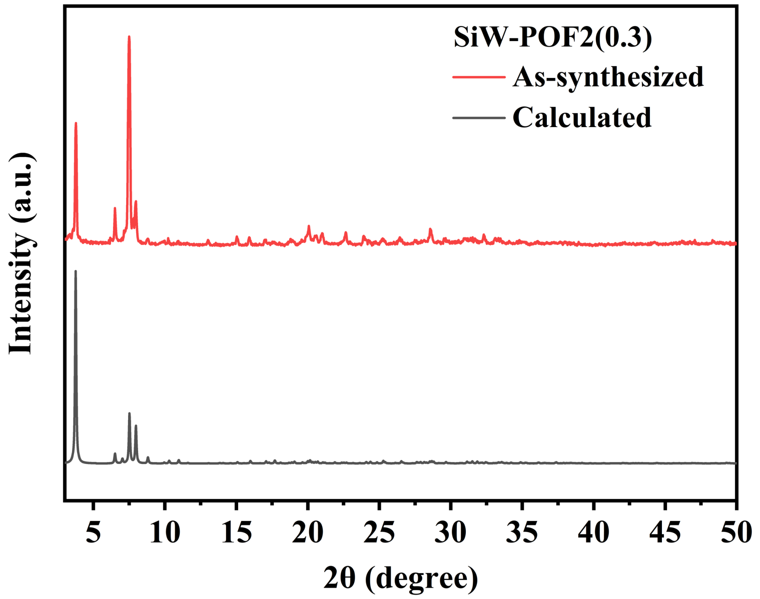

Supplementary Fig. 22: The PXRD pattern of SiW-POF2(0.3).

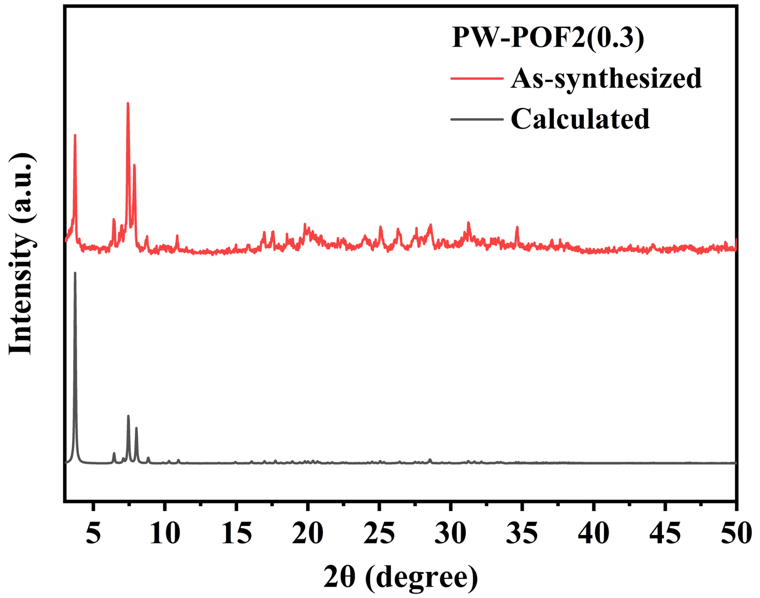

Supplementary Fig. 23: The PXRD pattern of PW-POF2(0.3).

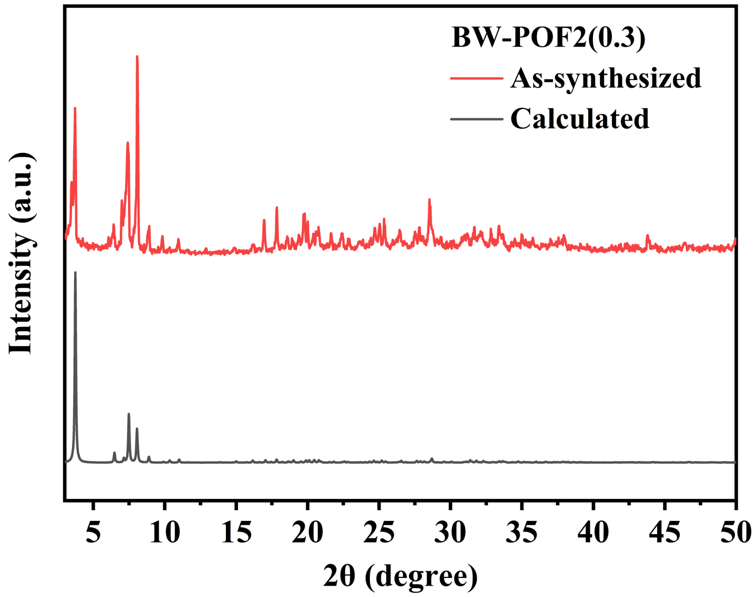

Supplementary Fig. 24: The PXRD pattern of BW-POF2(0.3).

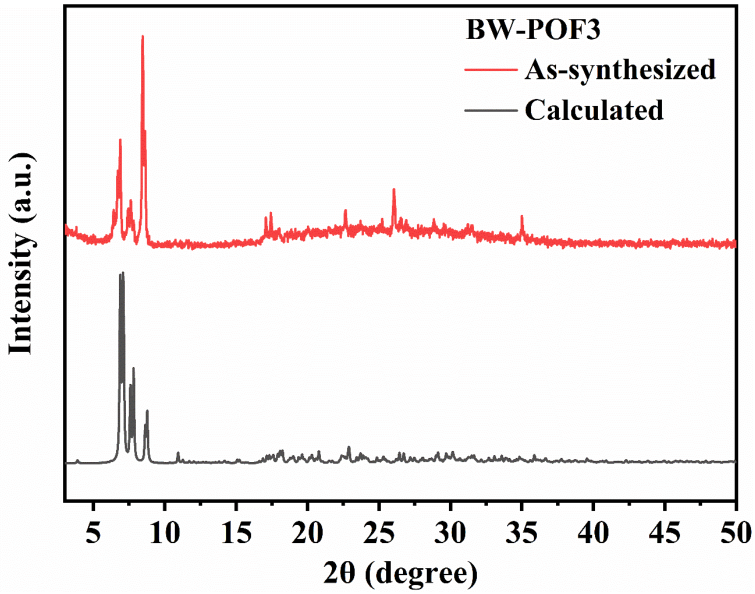

Supplementary Fig. 25: The PXRD pattern of BW-POF3.

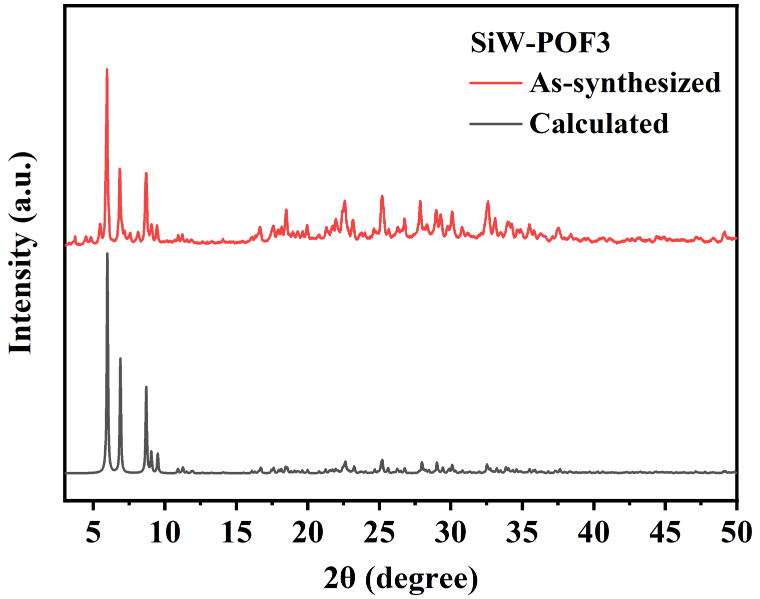

Supplementary Fig. 26: The PXRD pattern of SiW-POF3.

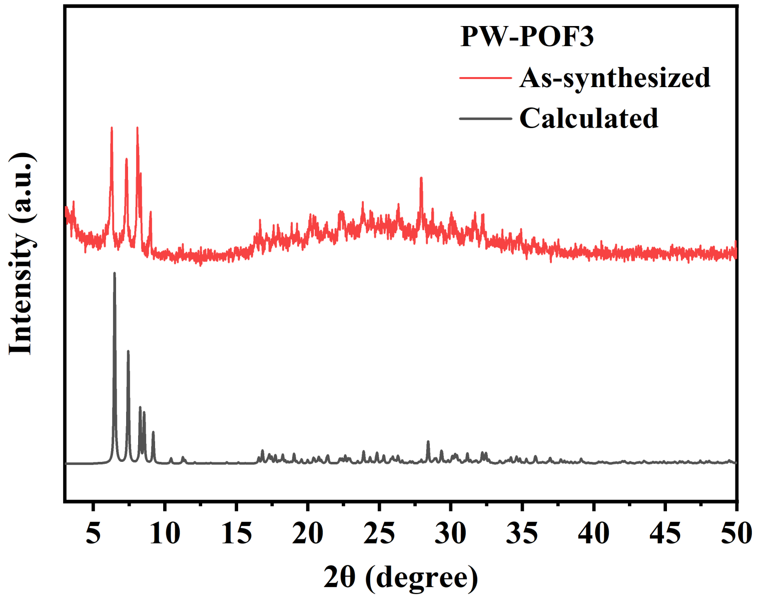

Supplementary Fig. 27: The PXRD pattern of PW-POF3.

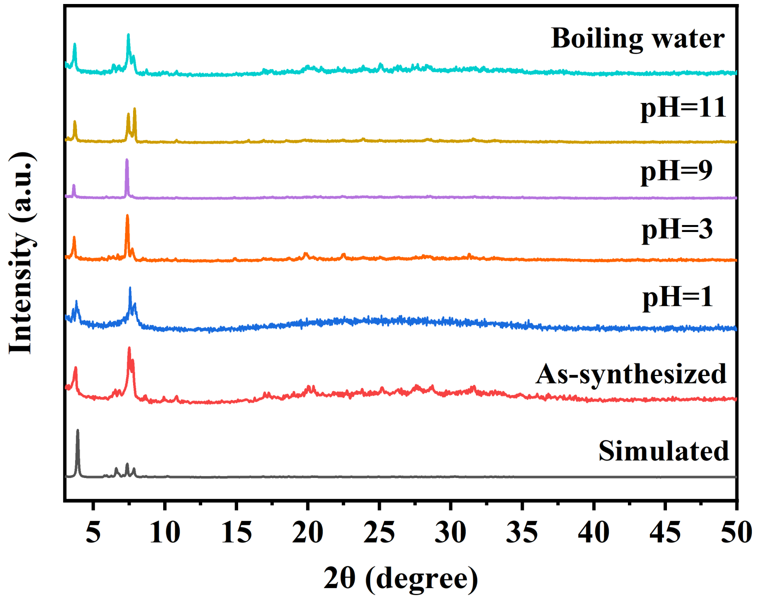

Supplementary Fig. 28: The PXRD patterns of SiW-POF1 under various treatments for 24 h.

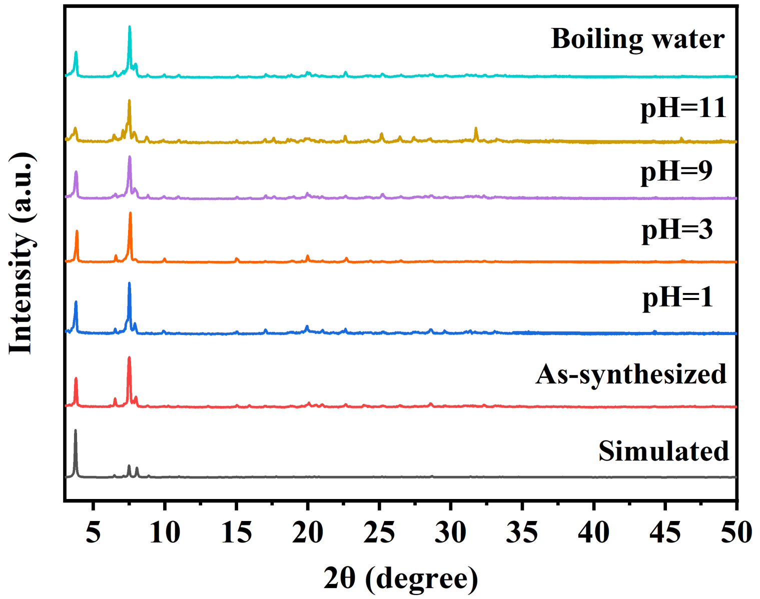

Supplementary Fig. 29: The PXRD patterns of SiW-POF2 under various treatments for 24 h.

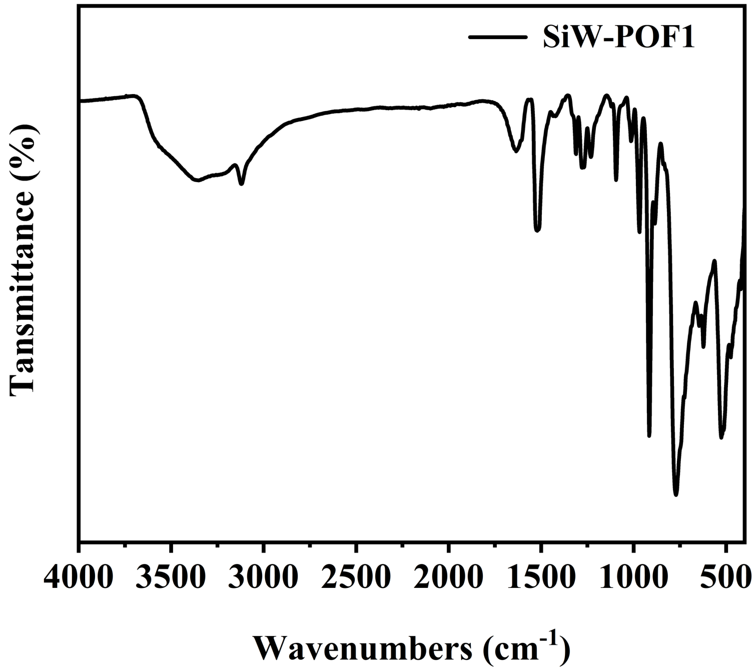

Supplementary Fig. 30: The FTIR spectrum of SiW-POF1.

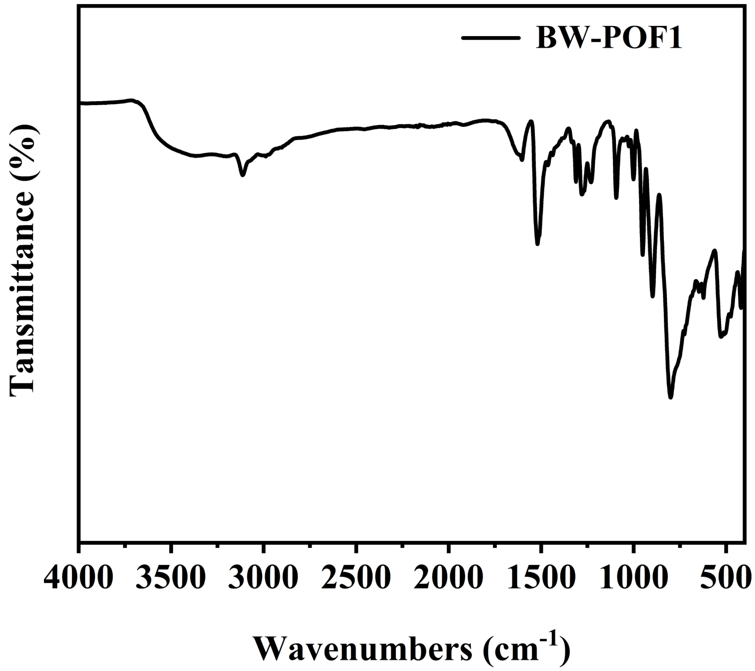

Supplementary Fig. 31: The FTIR spectrum of BW-POF1.

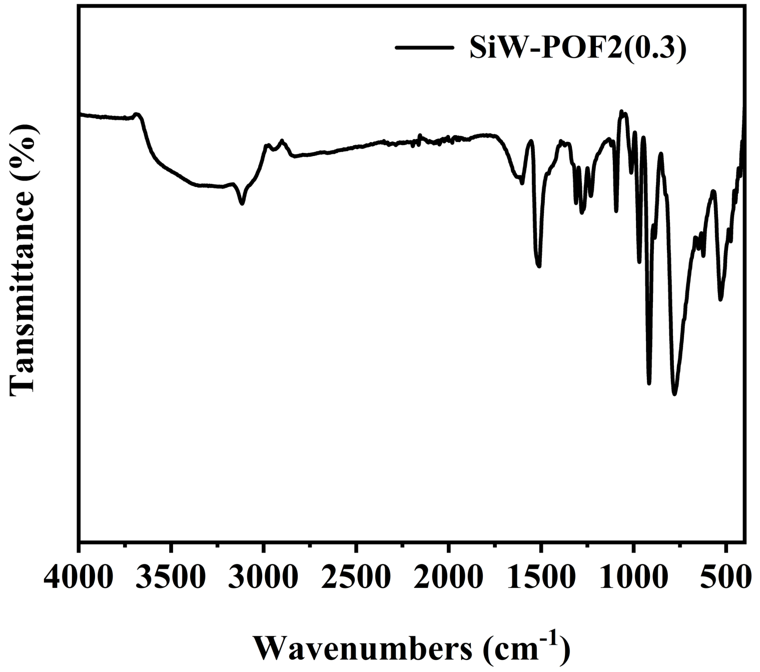

Supplementary Fig. 32: The FTIR spectrum of SiW-POF2(0.3).

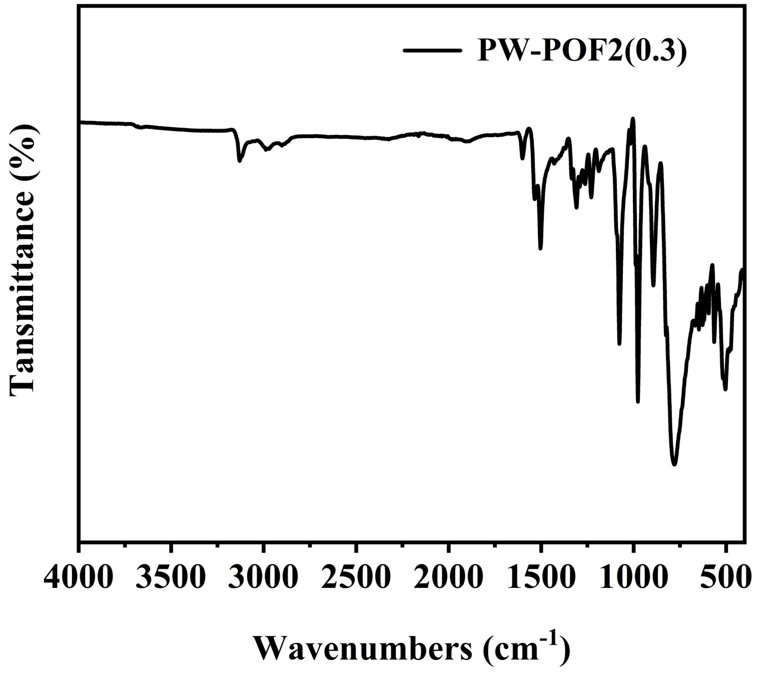

Supplementary Fig. 33: The FTIR spectrum of PW-POF2(0.3).

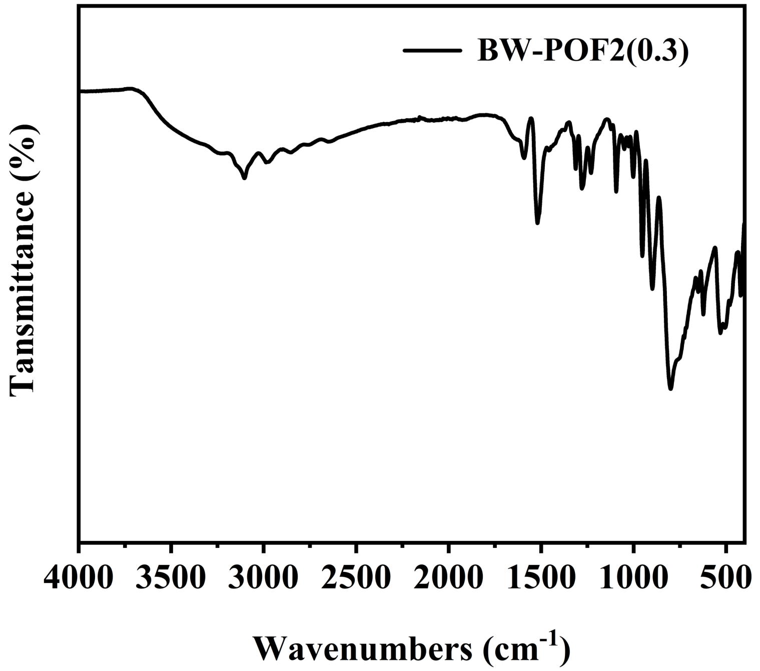

Supplementary Fig. 34: The FTIR spectrum of BW-POF2(0.3).

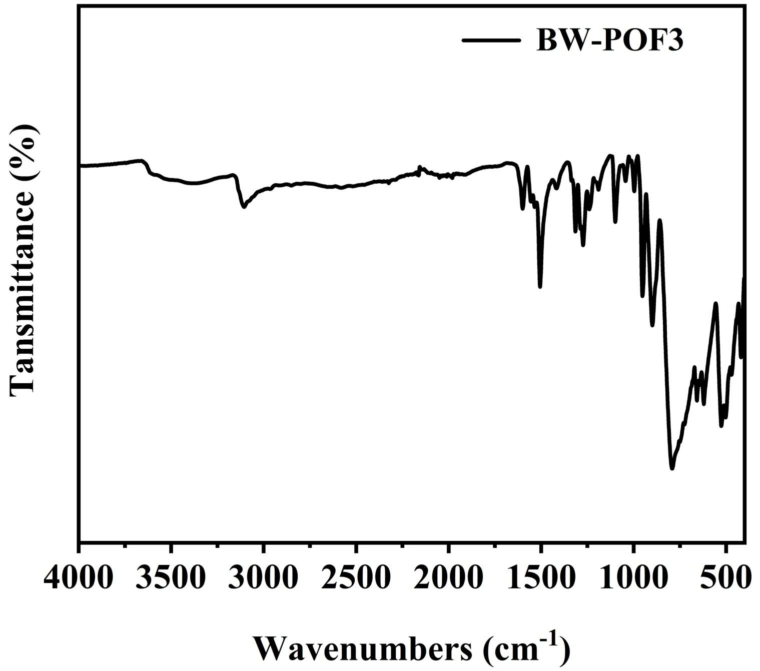

Supplementary Fig. 35: The FTIR spectrum of BW-POF3.

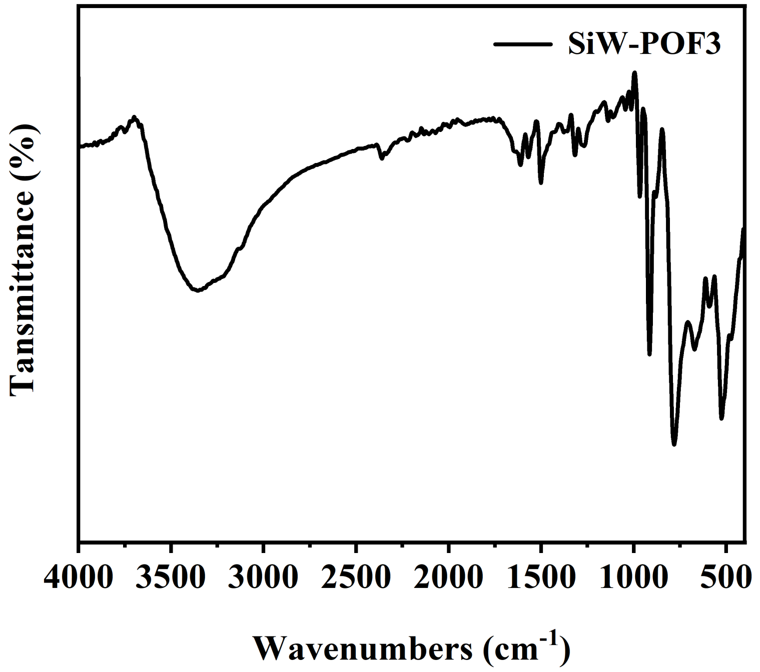

Supplementary Fig. 36: The FTIR spectrum of SiW-POF3.

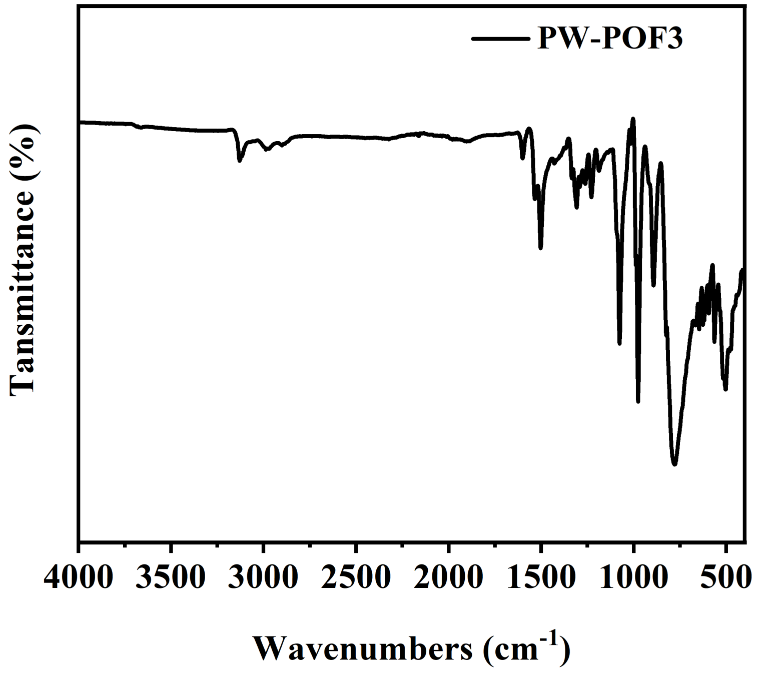

Supplementary Fig. 37: The PXRD pattern of PW-POF3.

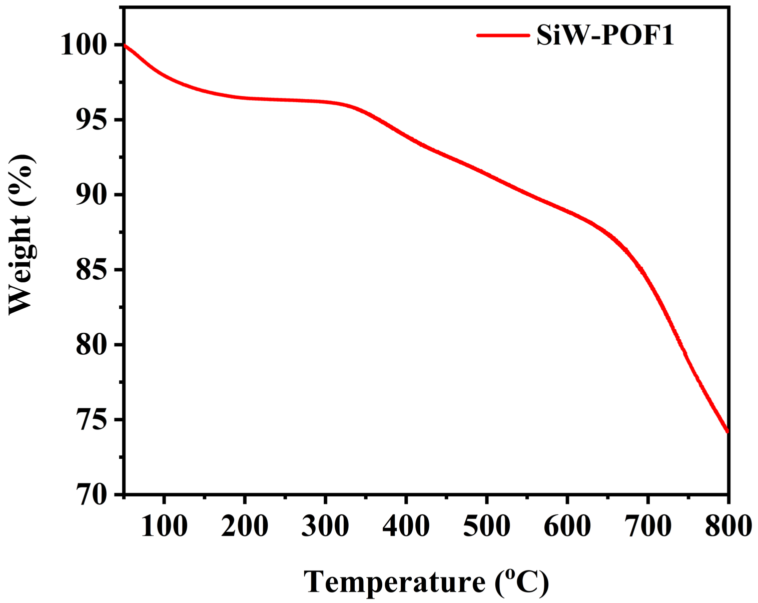

Supplementary Fig. 38: The thermogravimetric curve of SiW-POF1. The weight loss of 3.2% between r.t. and 150 °C corresponds to ~ 12 guest H_2O.

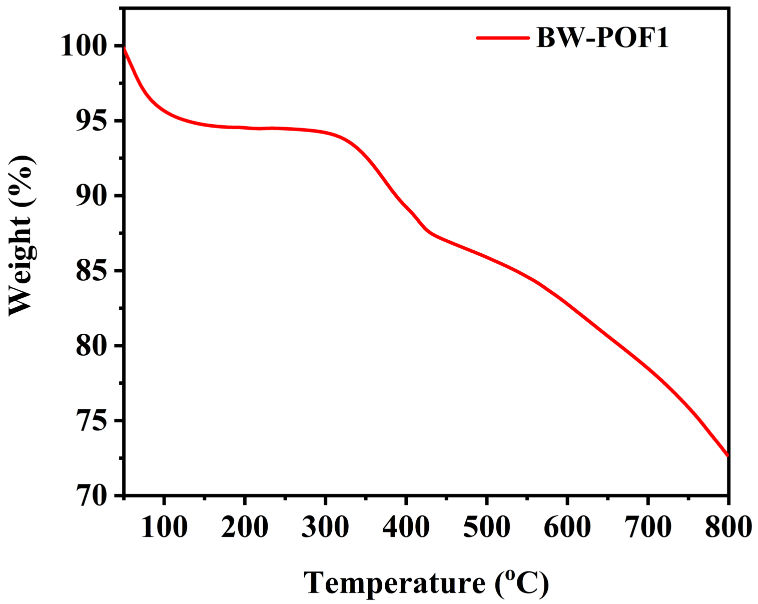

Supplementary Fig. 39: The thermogravimetric curve of BW-POF1. The weight loss of 5.3% between r.t. and 150 °C corresponds to ~ 12 guest H_2O.

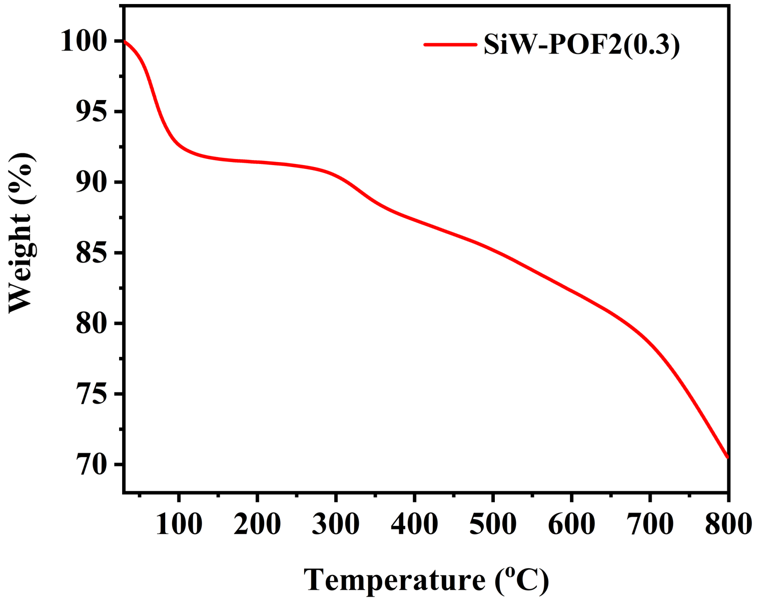

Supplementary Fig. 40: The thermogravimetric curve of SiW-POF2(0.3). The weight loss of 8.4% between r.t. and 150 °C corresponds to ~ 19 guest H_2O.

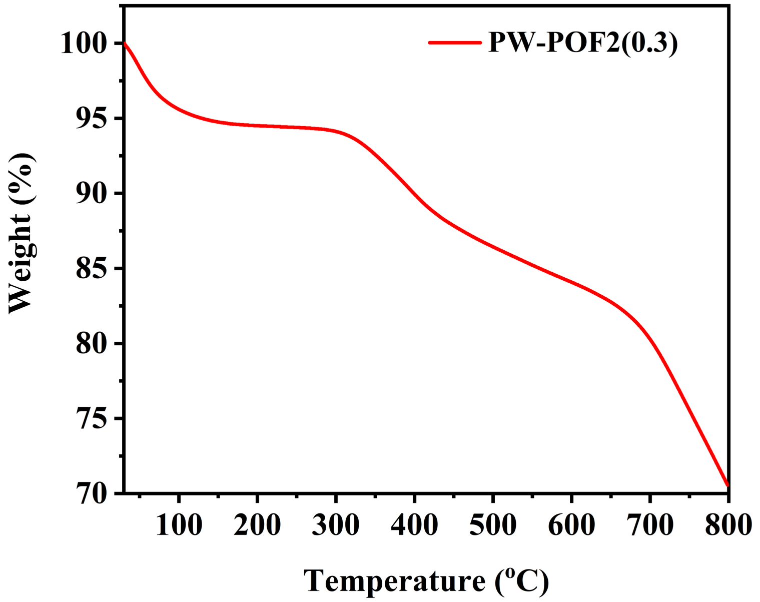

Supplementary Fig. 41: The thermogravimetric curve of PW-POF2(0.3). The weight loss of 5.3% between r.t. and 150 °C corresponds to ~ 12 guest H_2O.

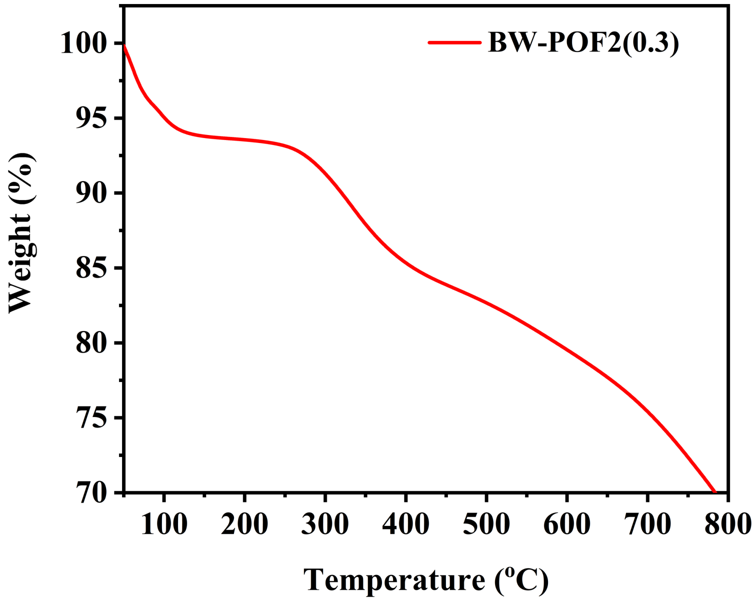

Supplementary Fig. 42: The thermogravimetric curve of BW-POF2(0.3). The weight loss of 6.3% between r.t. and 150 °C corresponds to ~ 15 guest H_2O.

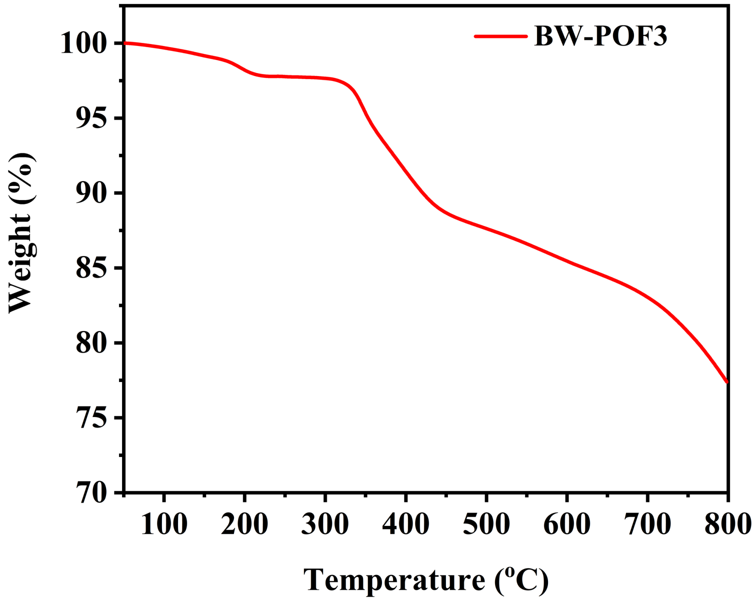

Supplementary Fig. 43: The thermogravimetric curve of BW-POF3. The weight loss of 1.0% between r.t. and 150 °C corresponds to ~ 2 guest H_2O.

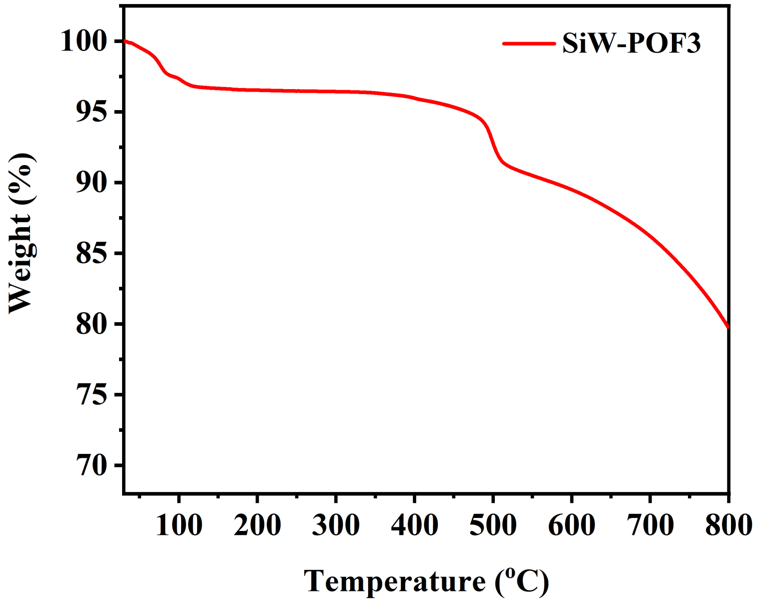

Supplementary Fig. 44: The thermogravimetric curve of SiW-POF3. The weight loss of 4.1% between r.t. and 150 °C corresponds to ~ 9 guest H_2O.

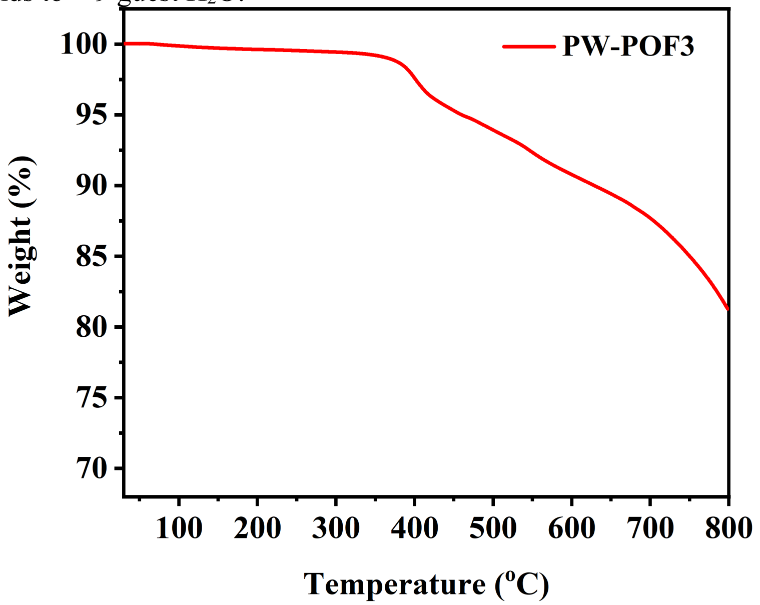

Supplementary Fig. 45: The thermogravimetric curve of PW-POF3. The weight loss of 0.5% between r.t. and 150 °C corresponds to ~ 1 guest H_2O.

1.3. NMR analysis of POFs

For NMR analysis, we dissolved a series of solid samples (10 mg) of SiW-POF2, PW-POF2 and BW-POF2 in DCl and DMSO-d_6. The ratio of imidazole to TTPA ligand was determined by ^1H NMR data.

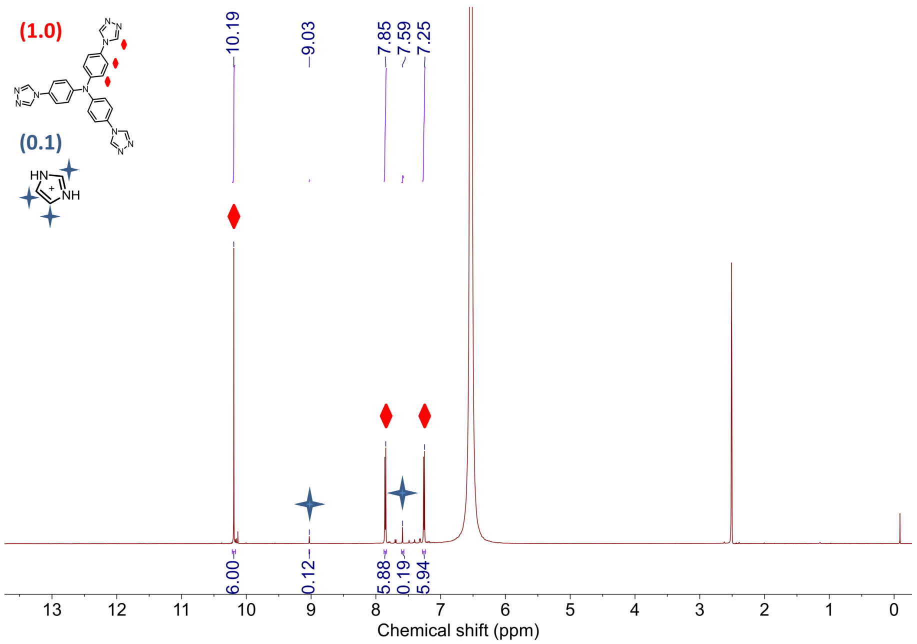

Supplementary Fig. 46: The NMR spectra of SiW-POF2(0.1).

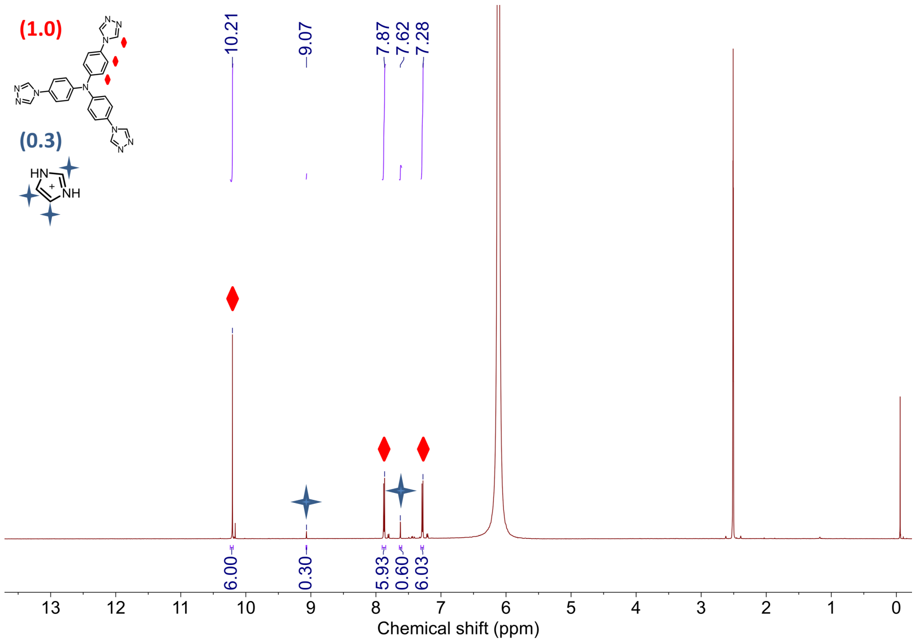

Supplementary Fig. 47: The NMR spectra of SiW-POF2(0.3).

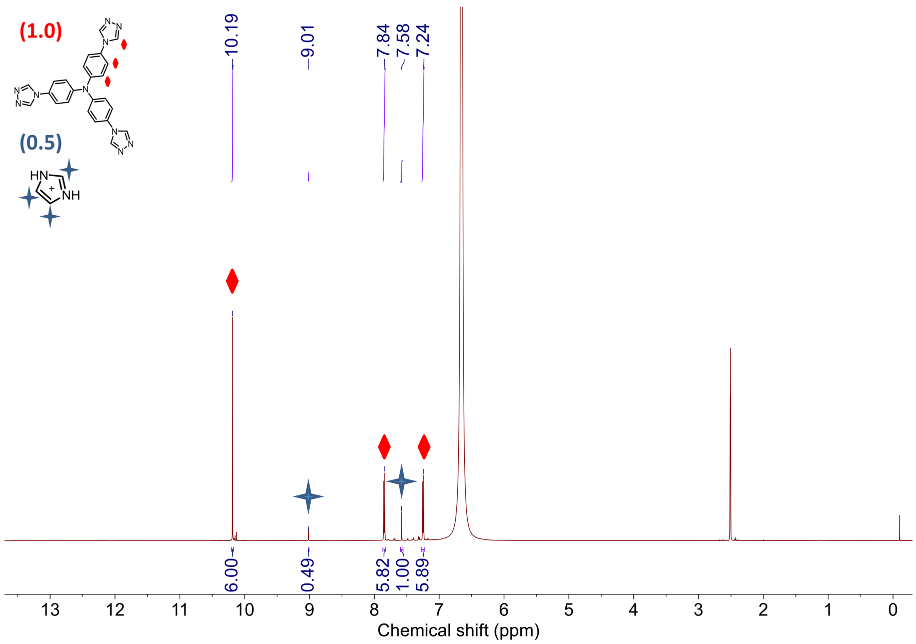

Supplementary Fig. 48: The NMR spectra of SiW-POF2(0.5).

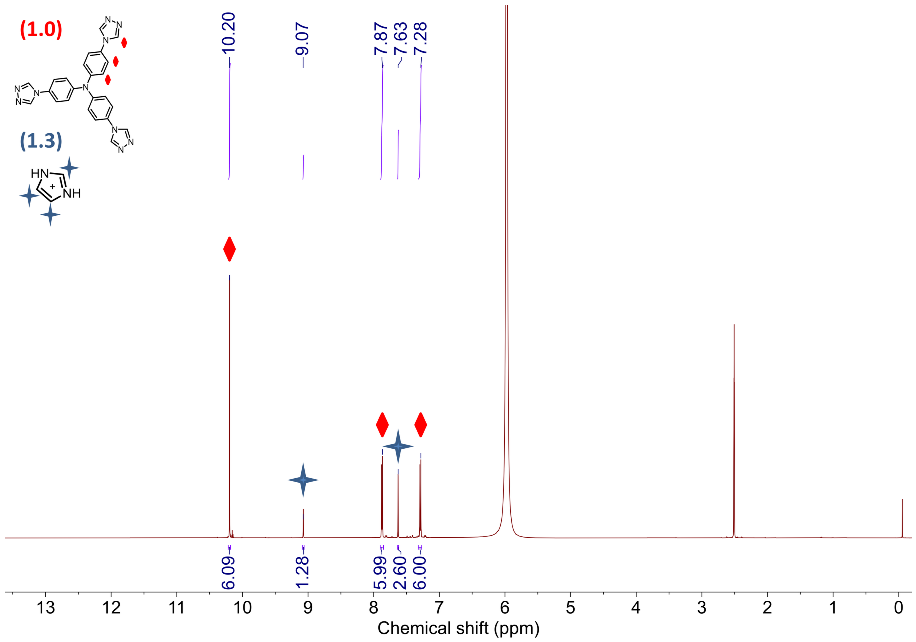

Supplementary Fig. 49: The NMR spectra of SiW-POF2(1.3).

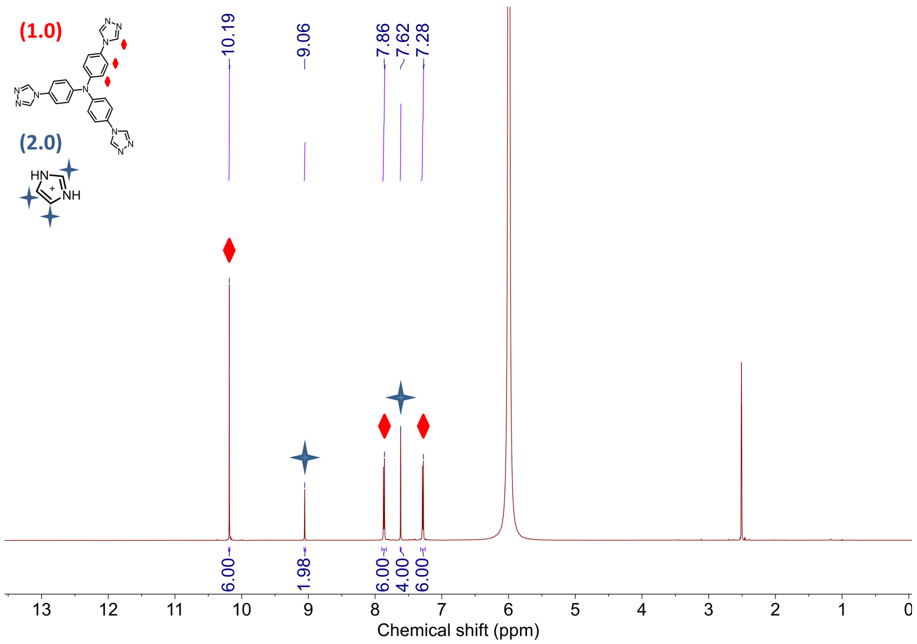

Supplementary Fig. 50: The NMR spectra of SiW-POF2(2.0).

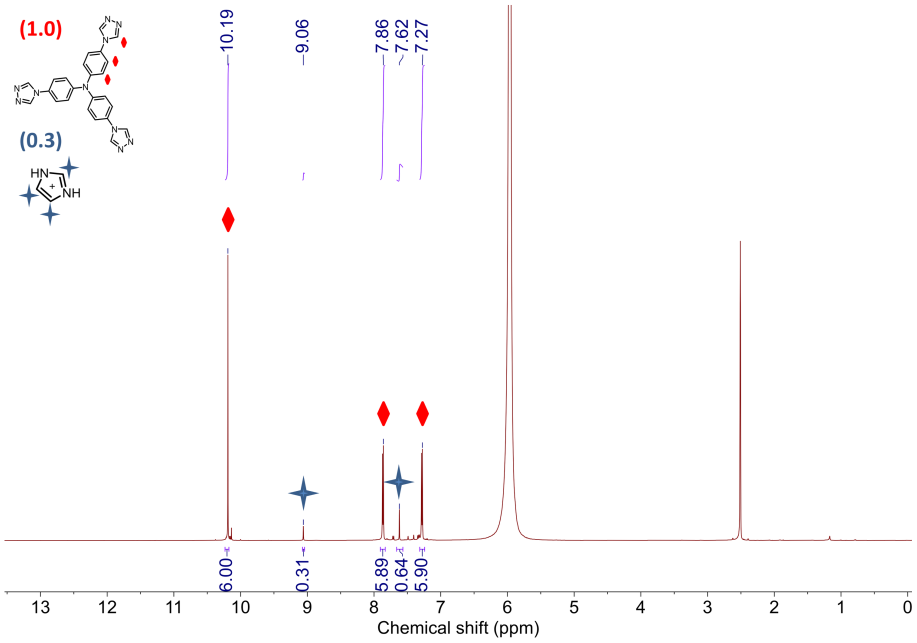

Supplementary Fig. 51: The NMR spectra of PW-POF2(0.3).

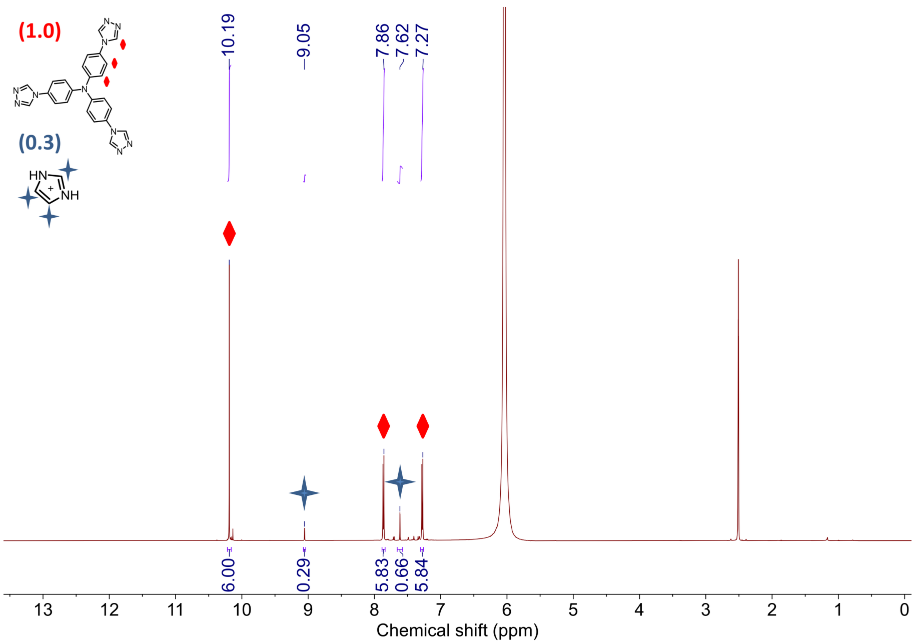

Supplementary Fig. 52: The NMR spectra of BW-POF2(0.3).

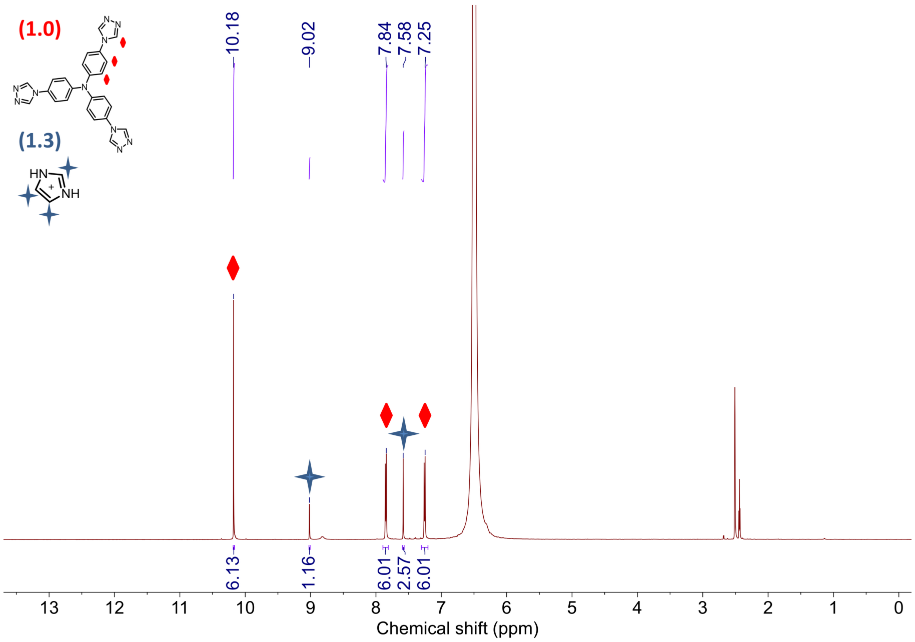

Supplementary Fig. 53: The NMR spectra of BW-POF2(1.3).

2. Proton Conductivity Experiment of POFs

First, the crystalline particles were grounded into powder separately. The powder was then put into a mold with an inner diameter of 10 mm and pressed into pellets under a pressure of 2 MPa and

measured the thickness with a digital micrometer. Second, pellets were sandwiched between two gold-plated electrodes with a thickness of approximately 1.0 mm, and Stanwood SC-80-CC-3 constant temperature and humidity chamber was used to control temperature and relative humidity. Third, the proton conductivity was measured using the alternating current (AC) impedance method of a Solartron SI 1260 Impedance/Gain Phase Analyzer, with a test frequency range of 1 Hz to 1 MHz and an applied voltage of 100 mV. For variable temperature and variable humidity electrochemical impedance (EIS) testing, the test is performed after equilibrating each test point for 30 minutes and 12 hours, respectively. Zview software was used to fit the impedance data. Extrapolation of the arc of the impedance spectrum to the X-axis gives resistance, the proton conductivity (σ) was calculated from the resistance value (R) and the value was calculated as follows:

$$\sigma = \frac{d}{RA}$$

where d is the pellet thickness (cm), R is the resistance (Ω), A is the cross-sectional area (cm^2), respectively.

The activation energy (Ea) was calculated from the linear Arrhenius curve, and the equation was as follows:

$$\sigma T = \sigma_0 \exp(\frac{-Ea}{k_B T})$$

where σ_0 is the pre-exponential factor, T is the absolute temperature and k_B is the Boltzmann constant.

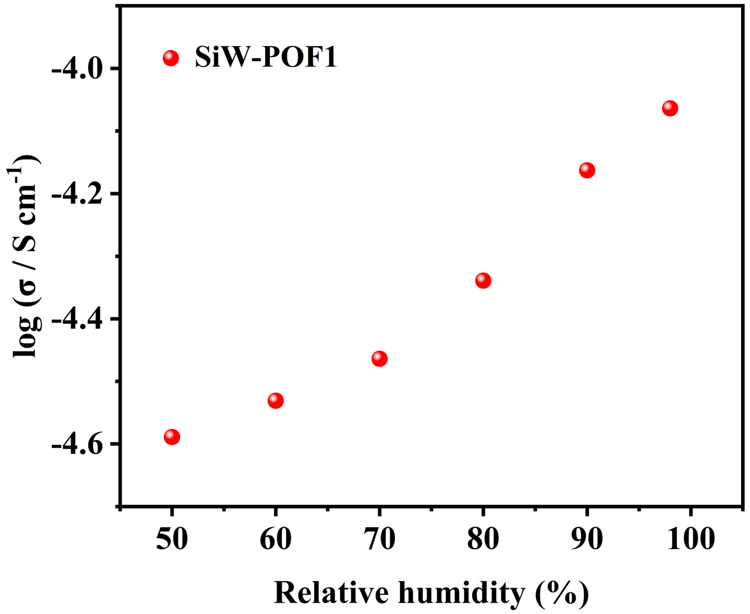

Supplementary Fig. 54: Nyquist plot of SiW-POF1 at various RH.

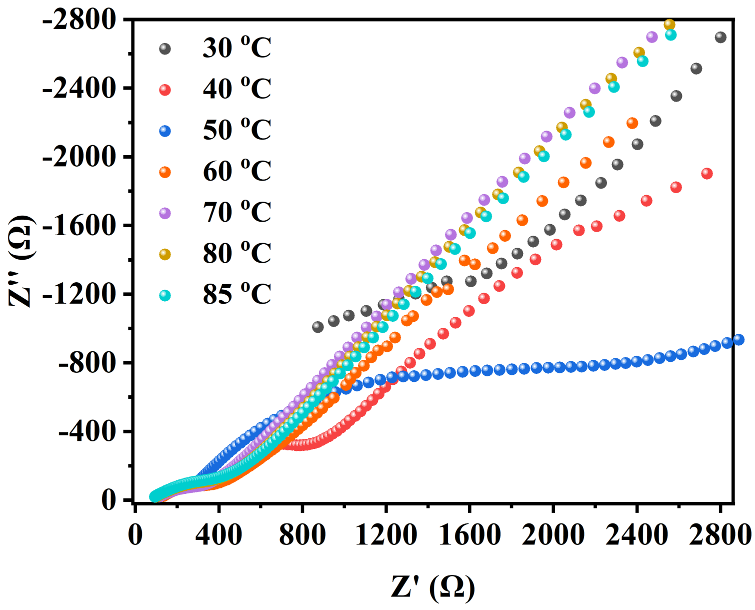

Supplementary Fig. 55: Impedance spectra of SiW-POF1 under 98% RH at various temperature.

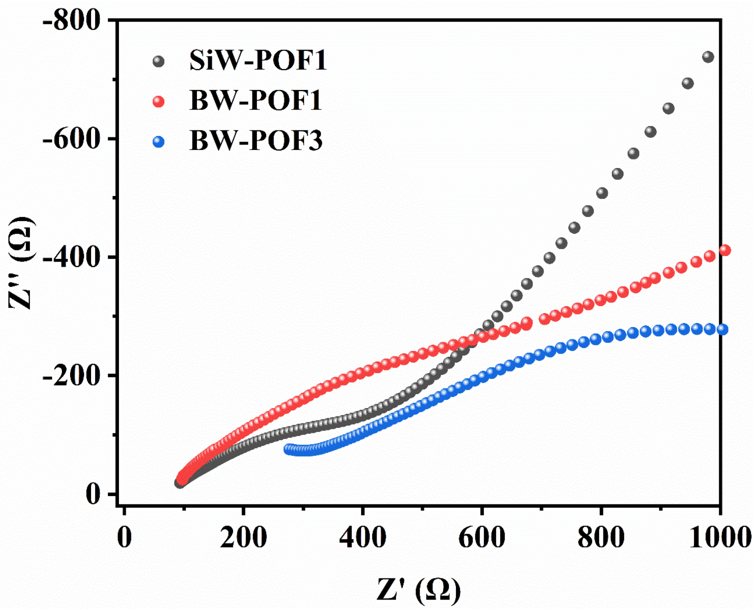

Supplementary Fig. 56: Comparison of impedance spectra for SiW-POF1 (black), BW-POF1 (red) and BW-POF3 (blue) under 85 °C with 98% RH.

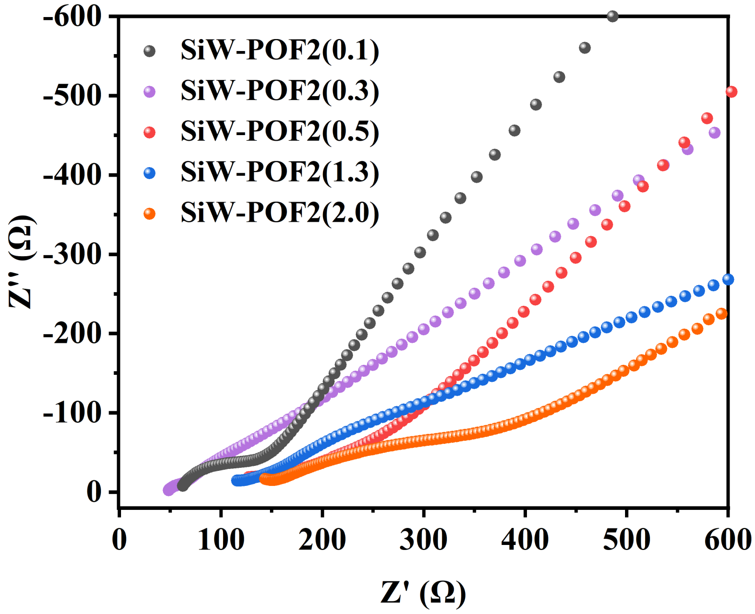

Supplementary Fig. 57: Comparison of impedance spectra for SiW-POF2 under 85 °C with 98% RH.

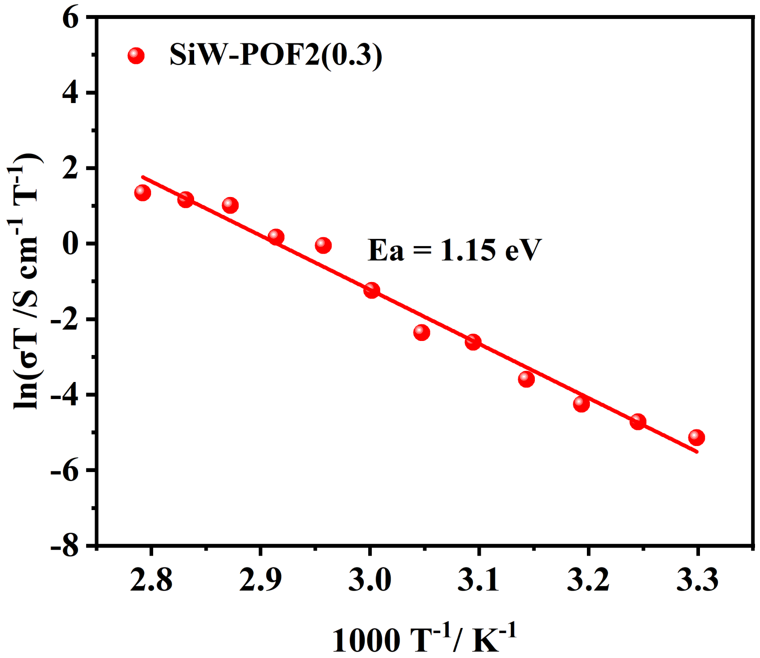

Supplementary Fig. 58: Arrhenius plots of proton conductivity for SiW-POF2(0.3) under 98% RH conditions.

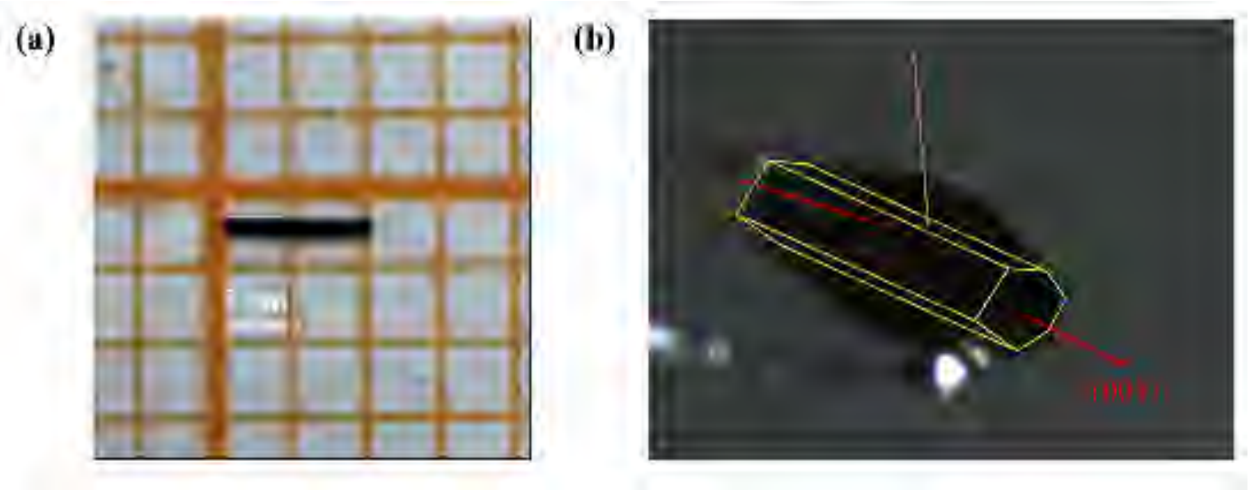

Supplementary Fig. 59: (a) The optical image of a SiW-POF2 crystal placed on a grid-patterned substrate with a scale bar of 1 cm. (b) Determination of the crystal face of a SiW-POF2 crystal.

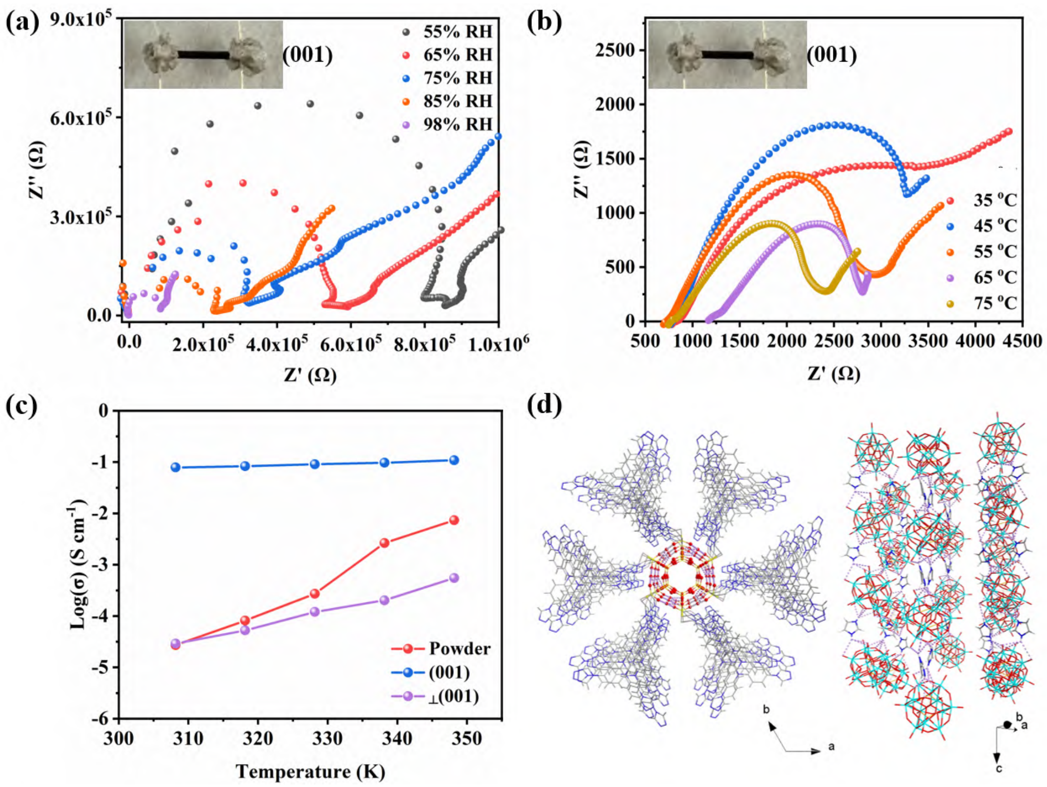

Supplementary Fig. 60: Nyquist plots of a SiW-POF2(0.3) single crystal measured along the (001) direction at 25 ℃ under 55 – 98% RH (a) and at 98% RH under different temperatures (b); (c) comparison of the temperature-dependent proton conductivities for SiW-POF2(0.3) single crystal along (001) direction and the direction perpendicular to (001) and pelletized SiW-POF2(0.3) sample; (d) top view of the proton conducting channel 1 and side view of the proton conducting channel 2 along *c* axis.

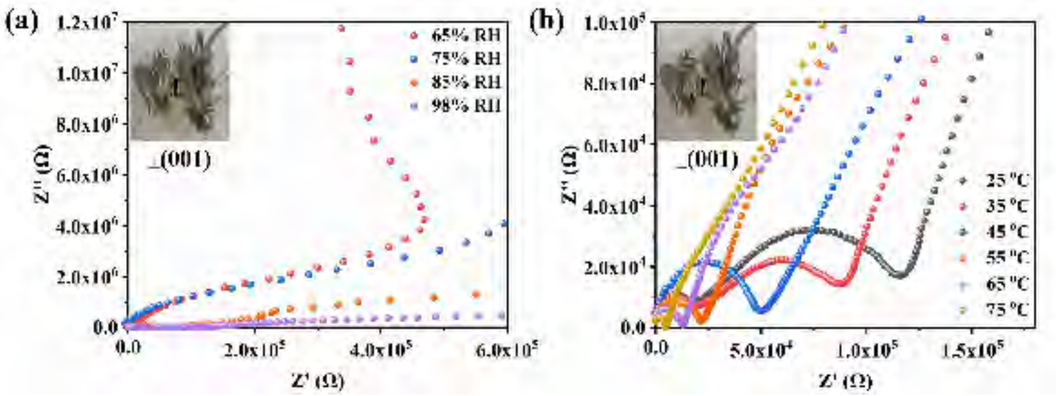

Supplementary Fig. 61: Nyquist plots of a SiW-POF2(0.3) single crystal measured along the direction perpendicular to (001) direction at 25 ℃ under 55 – 98% RH (a) and at 98% RH under different temperatures (b).

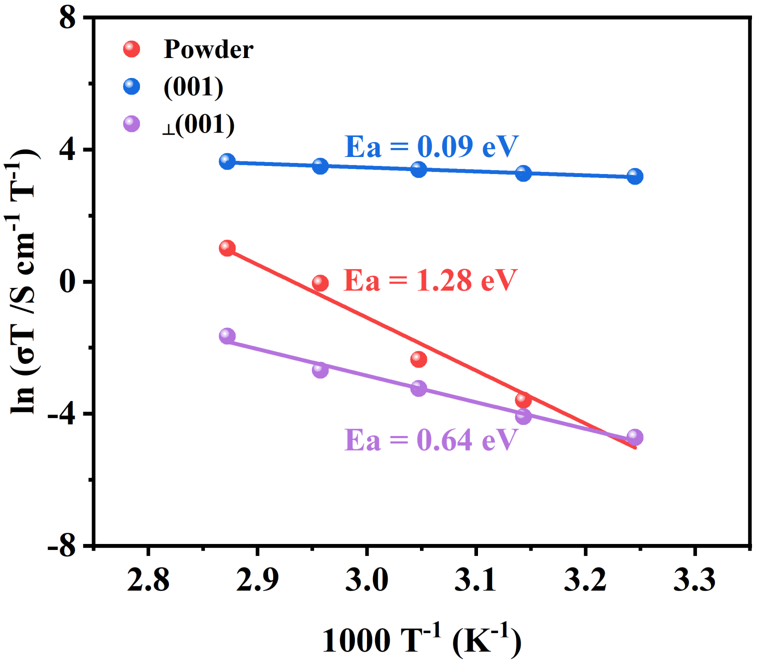

Supplementary Fig. 62: Arrhenius plots of pelletized SiW-POF2(0.3) sample and SiW-POF2(0.3) single crystal measured along (001) and the direction perpendicular to (001) at 98% RH under different temperatures.

3. Synthesis and structural characterization of TTPA-S and SiW-POF-S

Synthesis of TTPA-S

In order to better study the sulfonation rate, we specially synthesized TTPA-S as a reference for the verification of sulfonation rate by NMR. The specific synthesis conditions are as follows.

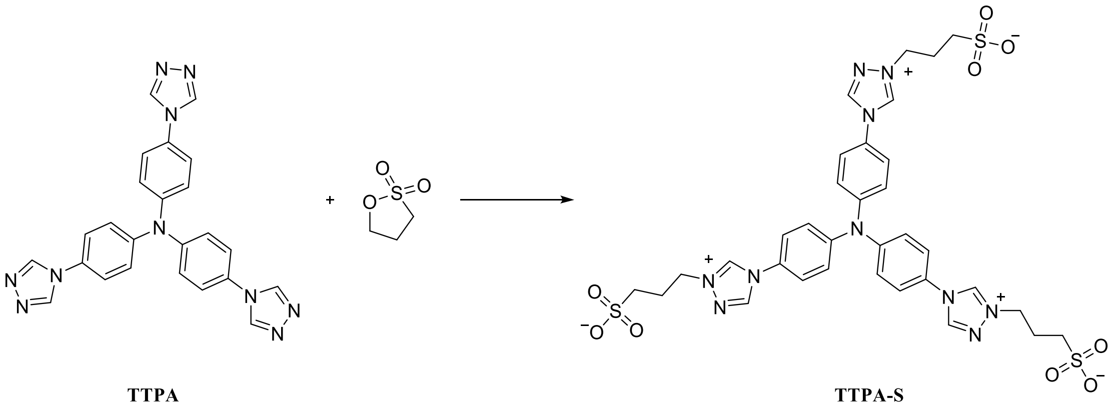

Supplementary Fig. 63: Synthetic procedure for TTPA-S

TTPA (46 mg, 0.1 mmol) and 1,3-propane sultone (0.3 mmol) were weighed and put into an glass bottle. After adding 6 mL of ethanol and stirring for 24 h at 25 ºC. After the reaction, the solution continues to heat and volatilize. When the solid product is precipitated, 0.5 mL H_2O and 0.5 mL DMA are added and heated at 80 °C for 12 h, and then slowly cooled to obtain colorless crystals. (20 mg, yield: 25 %).[8]

^{1}H NMR (600 MHz, DMSO-d_6): 10.91 (s, 3 H), 9.79 (s, 3 H), 7.82 (d, 6 H), 7.27 (d, 6 H), 4.53 (t, 6 H), 2.67 (t, 6 H), 2.23 (t, 6 H).

Crystallographic data and crystal structures of TTPA-S

Supplementary Table 11: Crystallographic data and structure refinement

Compound		TTPA-S	
formula	$C_{66}H_{76}N_{20}S_6O_{20}$	V /Å^3	4841.5(3)
Formula weight	1661.82	Z	2
Crystal system	Monoclinic	ρ calc /g·cm^{-3}	1.378
Space group	*P2₁*	R(int)	0.0800
a /Å	12.5050(7)	μ(MoKα) /mm^{-1}	2.265
b /Å	24.3339(14)	F(000)	1736
c /Å	13.1662(8)	R_1/wR_2 (I>2σ(I))[a]	0.1135, 0.3278
α/°	90	R_1/wR_2 (all data)	0.1224, 0.3413
β/°	91.988(3)	GooF (all data)[b]	1.180
γ/°	90	Data completeness	99.8 %

[a]$R_1 = \Sigma||Fo|-|Fc||/\Sigma|Fo|$; $wR_2 = \{\Sigma w[(Fo)^2-(Fc)^2]^2/\Sigma w[(Fo)^2]^2\}^{1/2}$

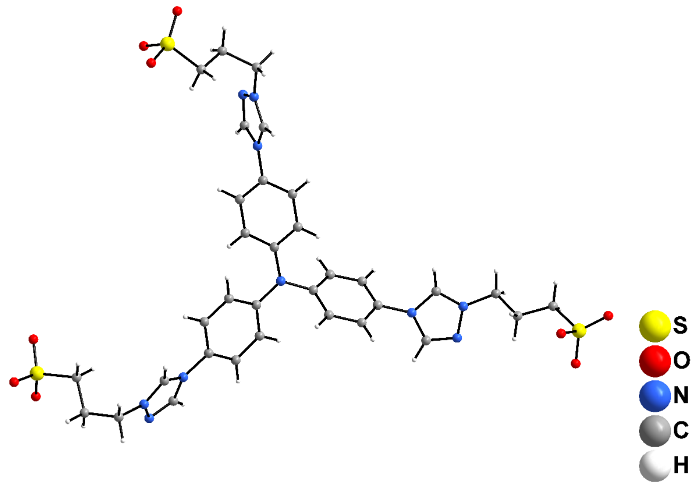

Supplementary Fig. 64: X-ray crystal structure of TTPA-S.

Synthesis of SiW-POF-S(60%) single crystal

SiW-POF2(0.3) (10 mg, 0.0025 mmol) was weighed in a glass bottle. Then 1,3-propane sultone (60 μL), 8 mL ethanol and a small amount of sodium bicarbonate aqueous solution were added and allowed to stand at room temperature for 2 weeks. The formula of SiW-POF2 (0.3)-S(60%) is determined as $H_4\{(C_{24}H_{18}N_{10})_{0.8}(C_{33}H_{36}N_{10}S_3O_9)_{1.2}(C_3N_2H_4)_{0.6}(SiW_{12}O_{40})\}\cdot 3H_2O$

Spectral characterization of POFs and POFs-S

Single crystal diffraction images

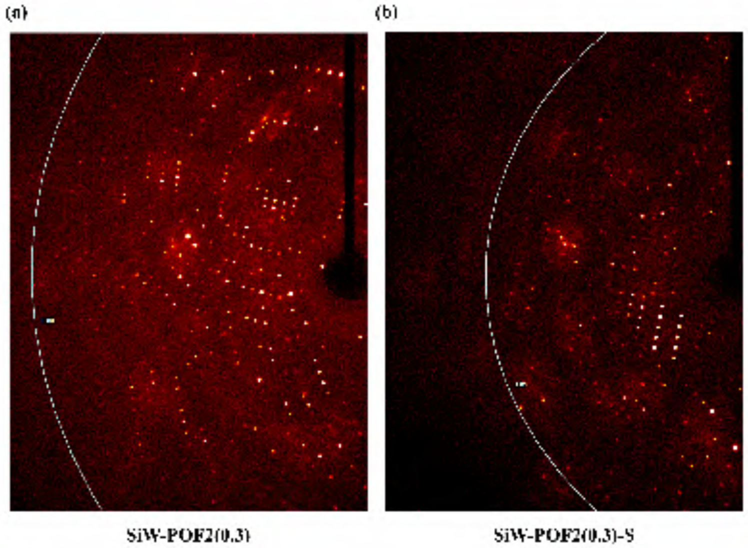

Supplementary Fig. 65: Single crystal diffraction images before and after post-modification

Supplementary Note 4. Refinement details regarding the disorder of alkyl sulfonic acid on SiW-POF2-S(60%)

At the final stage of refinement, after the main framework of the structure (the Keggin cluster and the TTPA tectons) had been established, residual electron density peaks were observed near the triazole ring of TTPA. These were modelled as propanesulfonate groups with occupancies of approximately 5% each. The geometries of the propanesulfonate groups were stabilized by restraining the C–C, C–S, and S–O bond distances and angles, and the thermal parameters of atoms within each group were restrained using SIMU. Because of the severe disorder and the proximity of these groups to symmetry elements of the space group, refinement in this region was unstable. To achieve convergence, a strong DAMP command was applied. Subsequent refinements of samples subjected to humidity treatments used the same structural model but required even more stringent restraints to maintain stability.

Supplementary Table 12: Crystallographic data and structure refinement of SiW-POF2-S(60%)–RH40

Compound		SiW-POF2-S(60%)-RH40	
formula	$C_{52.2}H_{36}N_{22}SiW_{12}O_{41.48}S_{0.4}$	V /Å^3	24704(2)
Formula weight	3882.22	Z	9
Crystal system	Trigonal	ρ calc /g·cm^{-3}	2.349
Space group	*R-3m*	R(int)	0.0532
a /Å	46.2683(13)	μ(MoKα) /mm^{-1}	12.610
b /Å	46.2683(13)	F(000)	15691
c /Å	13.3249(9)	R_1/wR_2 (I>2σ(I))[a]	0.0793, 0.2234
α/°	90	R_1/wR_2 (all data)	0.0980, 0.2493
β/°	90	GooF (all data)[b]	1.095
γ/°	120	Data completeness	99.9 %

[a]$R_1 = \sum||Fo|-|Fc||/\sum|Fo|$; $wR_2 = \{\sum w[(Fo)^2-(Fc)^2]^2/\sum w[(Fo)^2]^2\}^{1/2}$

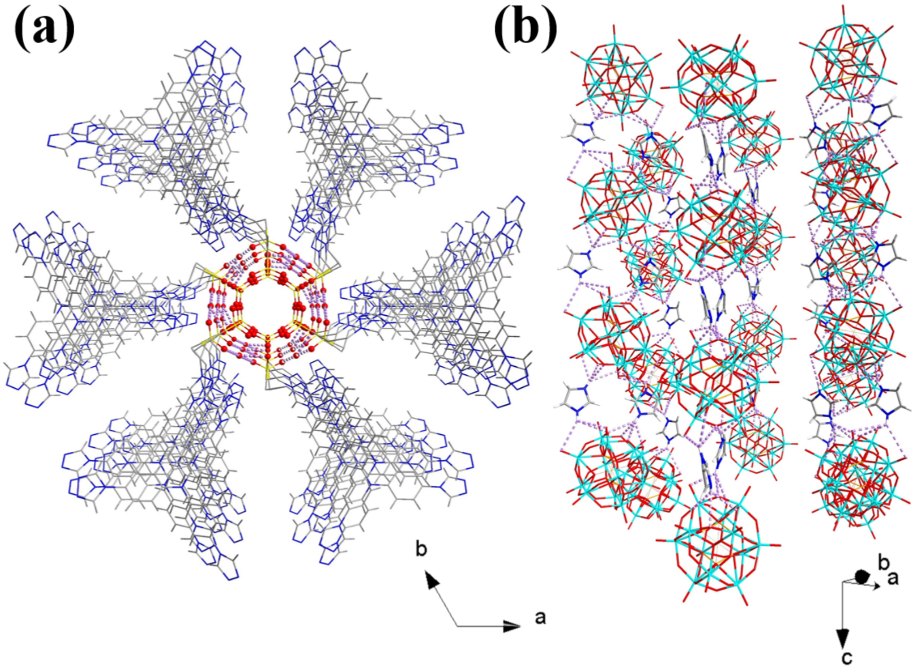

Supplementary Fig. 66: (a) Top view of the proton conducting channel 1 in SiW-POF2(0.3)-S(60%) along *c* axis; (b) side view of the proton conducting channel 2 in SiW-POF2(0.3)-S(60%), which also aligns along *c* axis.

Supplementary Note 5. Determination of the expansion of Unit cells of SiW-POF-S(60%) single crystals under different RH

We placed the single crystals of SiW-POF2-S(60%) in a sealed glass bottle with specific RH (40%-98%) at room temperature for 24 h. Then the fresh crystals are picked up and immediately subjected to Unit cell determination by single-crystal X-ray diffraction. The data of unit cells under different RH is summarized in **Supplementary Table 13**. In addition to the crystal data collected under 40% RH (SiW-POF2(0.3)-S(60%) in the manuscript), SiW-POF2-S(60%)-RH85 has also been recorded with crystal data, which revealed that more guest water molecule is accommodated in the structure (**Supplementary Fig. 67**).

Supplementary Table 13: Unit cell parameters collected for SiW-POF-S(60%) at different humidity

Compounds	Condition (RH)	Unit cell parameters
SiW-POF2-S(60%)-RH40	40%	a=46.27(15), b=46.27(15), c=13.32(5)<br>α=90, β=90, γ=120<br>V=24704(160)
SiW-POF2-S(60%)-RH50	53%	a=46.33(5), b=46.33(5), c=13.29(2)<br>α=90, β=90, γ=120<br>V=24710(60)
SiW-POF2-S(60%)-RH60	68%	a=46.73(2), b=46.73(2), c=13.18(1)<br>α=90, β=90, γ=120<br>V=24925(20)
SiW-POF2-S(60%)-RH75	75%	a=46.60(1), b=46.60(1), c=13.42(1)<br>α=90, β=90, γ=120<br>V=25251(1)
SiW-POF2-S(60%)-RH85	85%	a=46.94(2), b=46.94(2), c=13.35(1)<br>α=90, β=90, γ=120<br>V=25499(2)
SiW-POF2-S(60%)-RH98	98%	a=47.17(2), b=47.17(2), c=13.39(1)<br>α=90, β=90, γ=120<br>V=25800(20)

Supplementary Table 14: Crystallographic data and structure refinement of SiW-POF2-S(60%)–RH85

Compound		SiW-POF2-S(60%)–RH85	
formula	$C_{52.2}H_{33}N_{22}SiW_{12}O_{41.44}S_{0.4}$	V /Å^3	25497(2)
Formula weight	3878.56	Z	9
Crystal system	Trigonal	ρ calc /g·cm^{-3}	2.273
Space group	*R-3m*	R(int)	0.0411
a /Å	46.9483(13)	μ(MoKα) /mm^{-1}	11.191
b /Å	46.9483(13)	F(000)	15661
c /Å	13.3575(9)	R_1/wR_2 (I>2σ(I))[a]	0.0900, 0.2363
α/°	90	R_1/wR_2 (all data)	0.0905, 0.2368
β/°	90	GooF (all data)[b]	1.021
γ/°	120	Data completeness	99.6 %

[a]$R_1 = \sum||Fo|-|Fc||/\sum|Fo|$; $wR_2 = \{\sum w[(Fo)^2-(Fc)^2]^2/\sum w[(Fo)^2]^2\}^{1/2}$

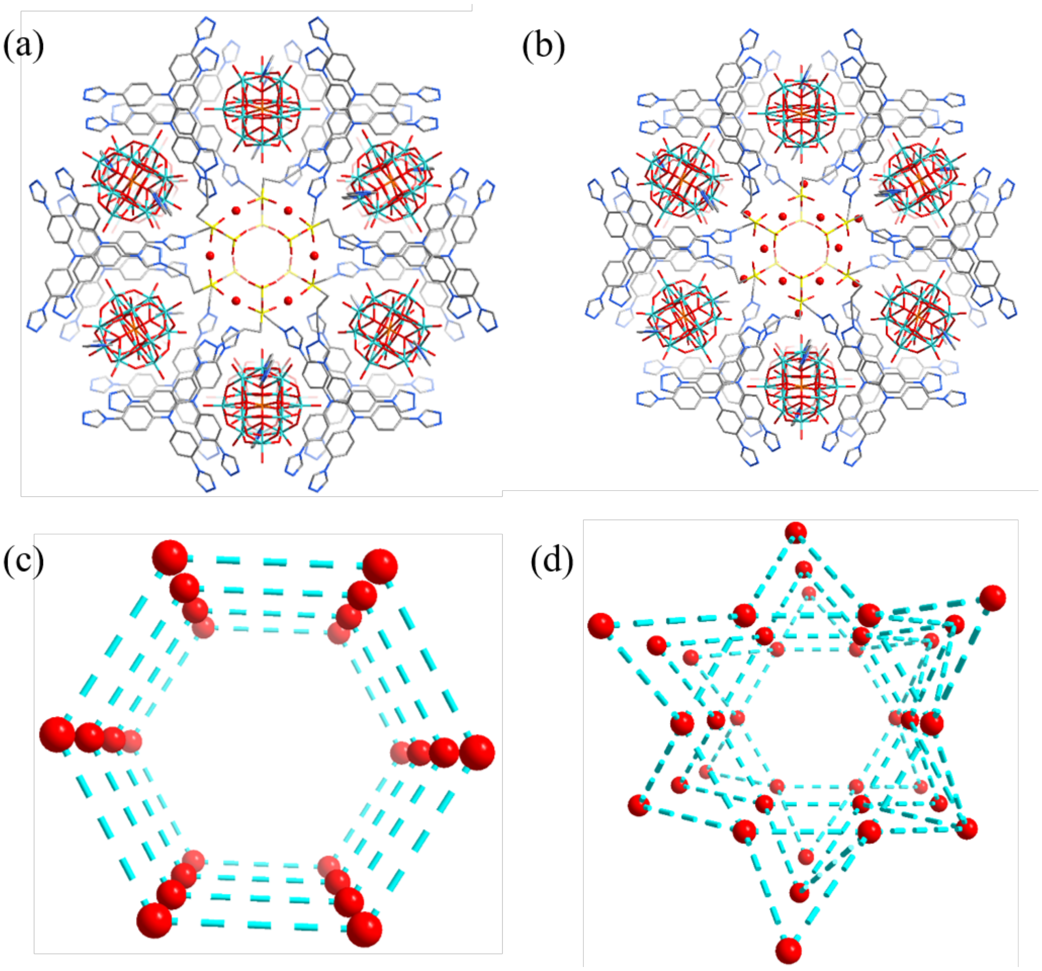

Supplementary Fig. 67: View of the guest water in the open channels of (a) SiW-POF2-S(60%)-RH40 and (b) SiW-POF2-S(60%)-RH85 along *c* axis; Visualization of the water hydrogen bond networks in (c) SiW-POF2-S(60%)-RH40 and (d) SiW-POF2-S(60%)-RH85 along *c* axis. The guest water molecules are highlighted as red balls, while the networks are constructed by connecting water molecules with dotted cyan lines. The data of SiW-POF2-S(60%)-RH40 and SiW-POF2-S(60%)-RH85 were collected at 150 K, 40% RH and 150 K, 85% RH, respectively.

NMR and other spectral analysis of POF-S

For NMR analysis, we dissolved a series of solid samples (10 mg) of SiW-POF2-S and SiW-POF1-S in DCl and DMSO-d_6. Here S denotes sulfonate and the values in the bracket represent the percentage of TTPA that is modified by sulfonation. These values are deduced from ^{1}H NMR study by adopting the same procedure of determining the ratio of imidazole/TTPA/TTPA-S.

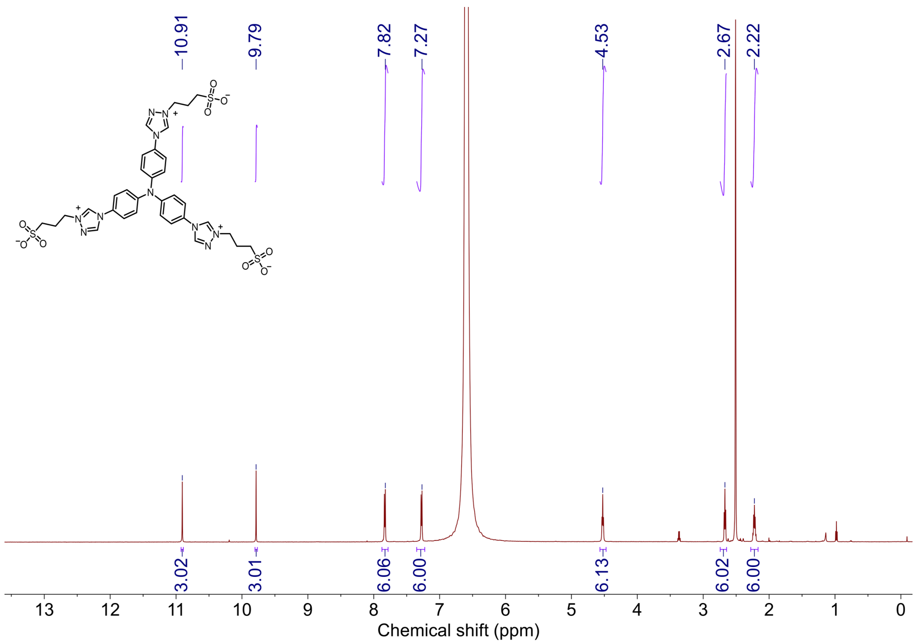

Supplementary Fig. 68: The NMR spectra of TTPA-S.

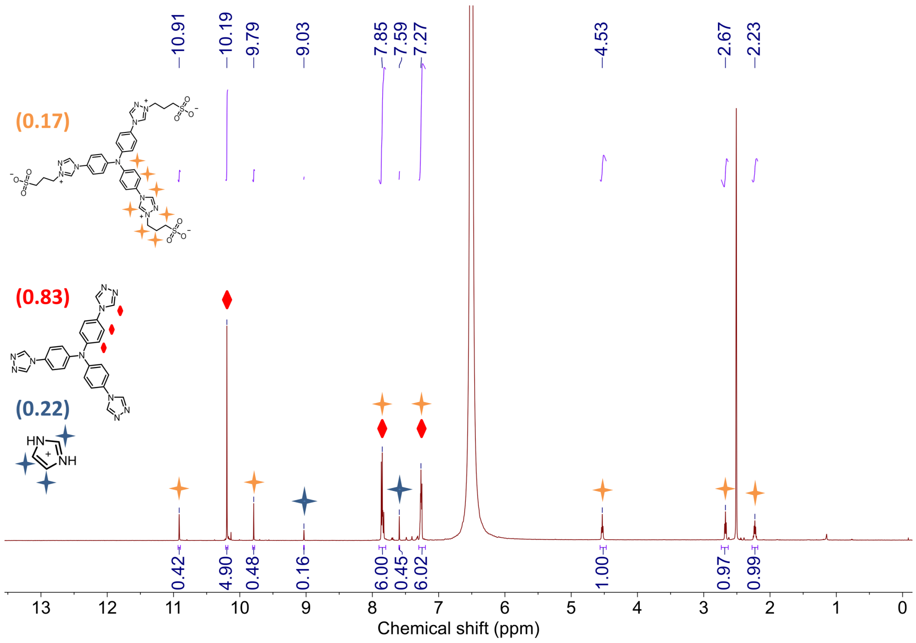

Supplementary Fig. 69: The NMR spectra of SiW-POF2(0.3)-S(17%).

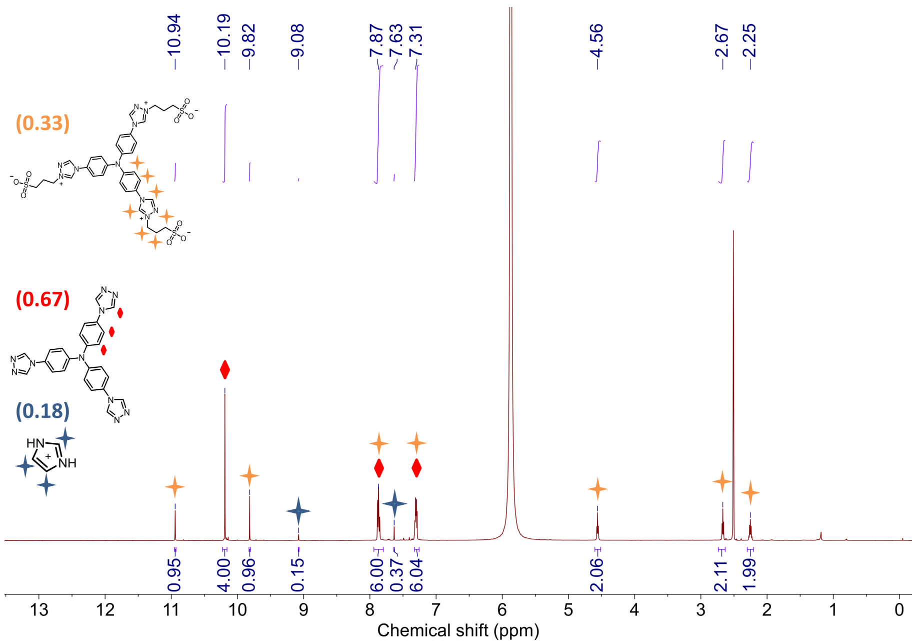

Supplementary Fig. 70: The NMR spectra of SiW-POF2(0.3)-S(33%).

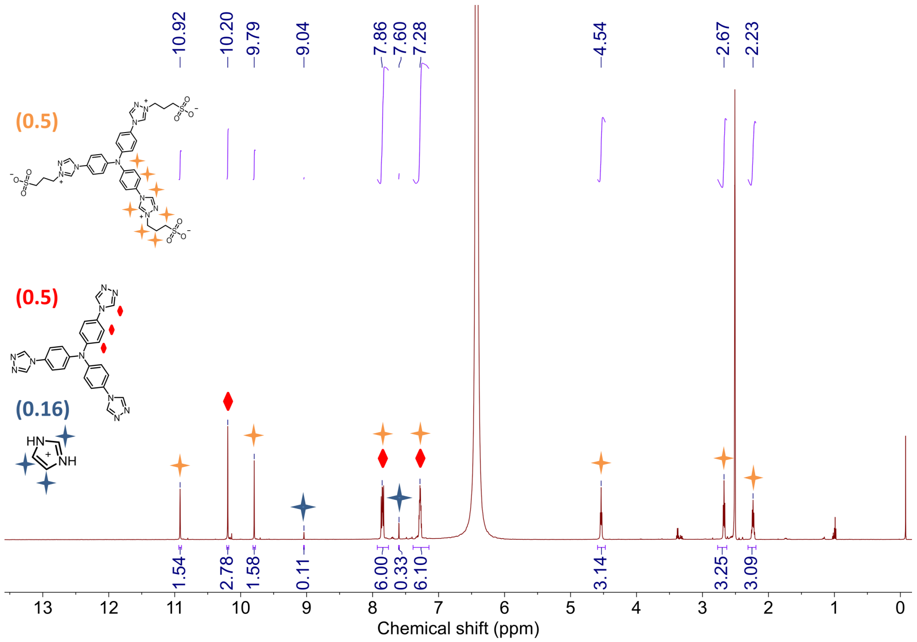

Supplementary Fig. 71: The NMR spectra of SiW-POF2(0.3)-S(50%).

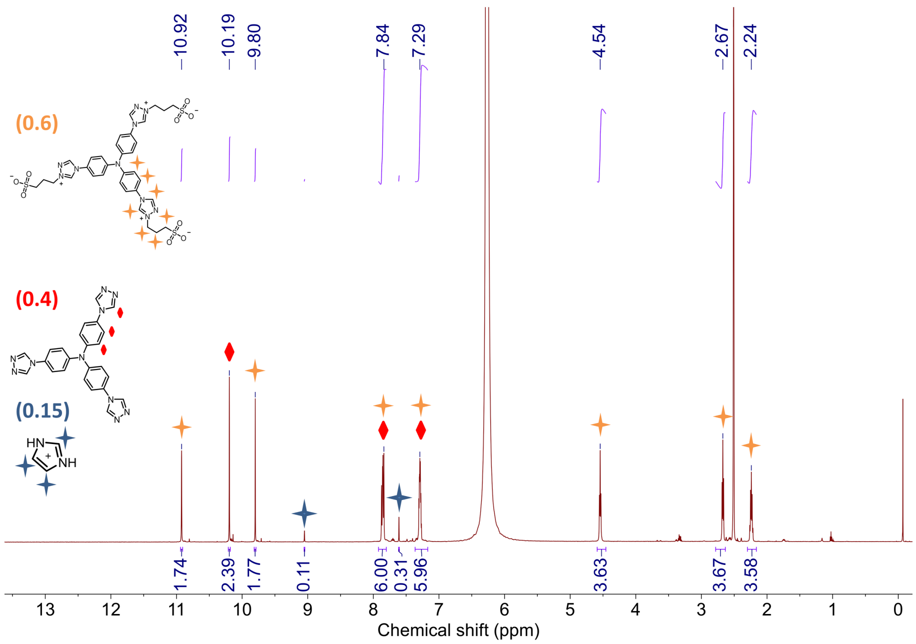

Supplementary Fig. 72: The NMR spectra of SiW-POF2(0.3)-S(60%).

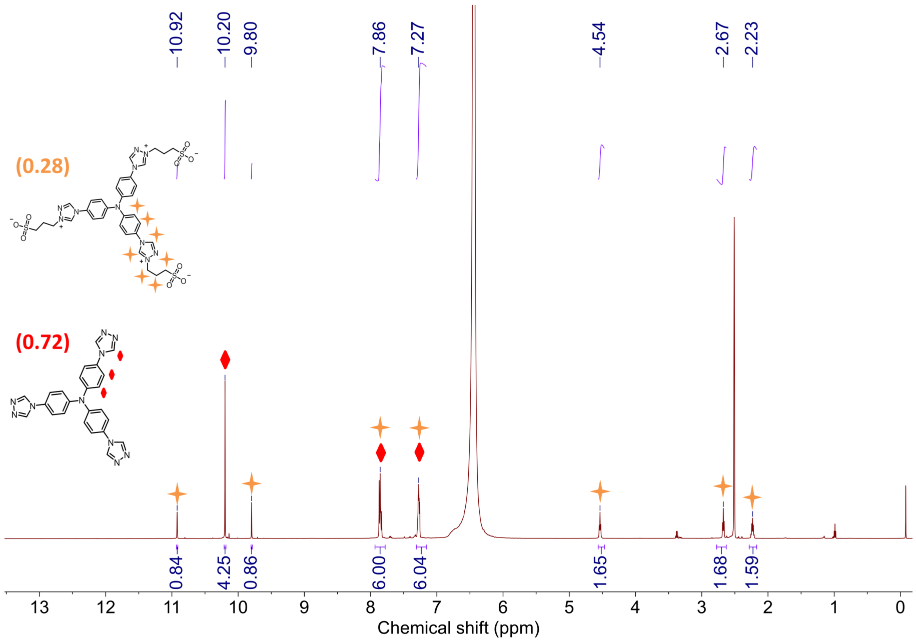

Supplementary Fig. 73: The NMR spectra of SiW-POF1-S(28%).

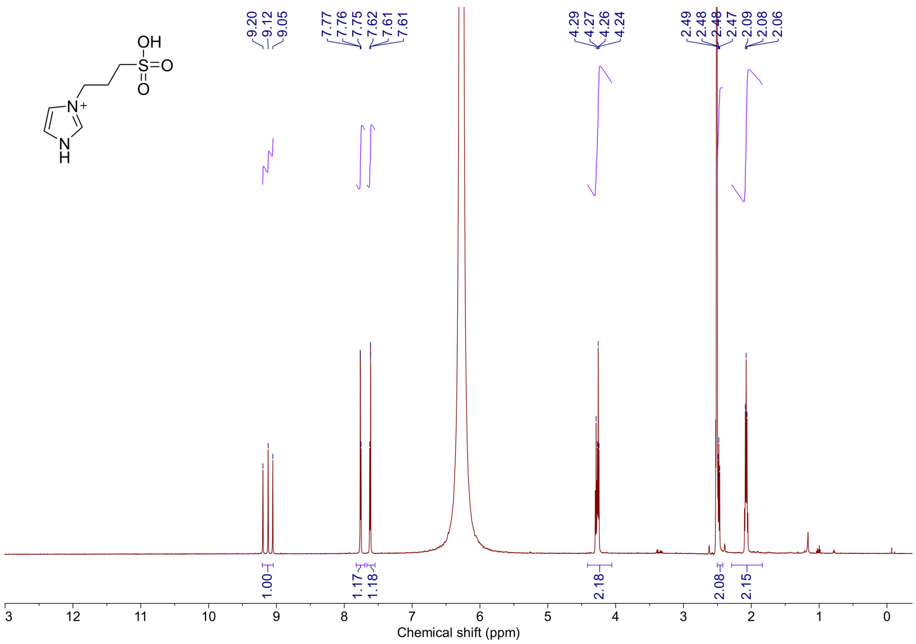

Supplementary Fig. 74: The ^{1}H NMR spectrum of sulfonated imidazolium

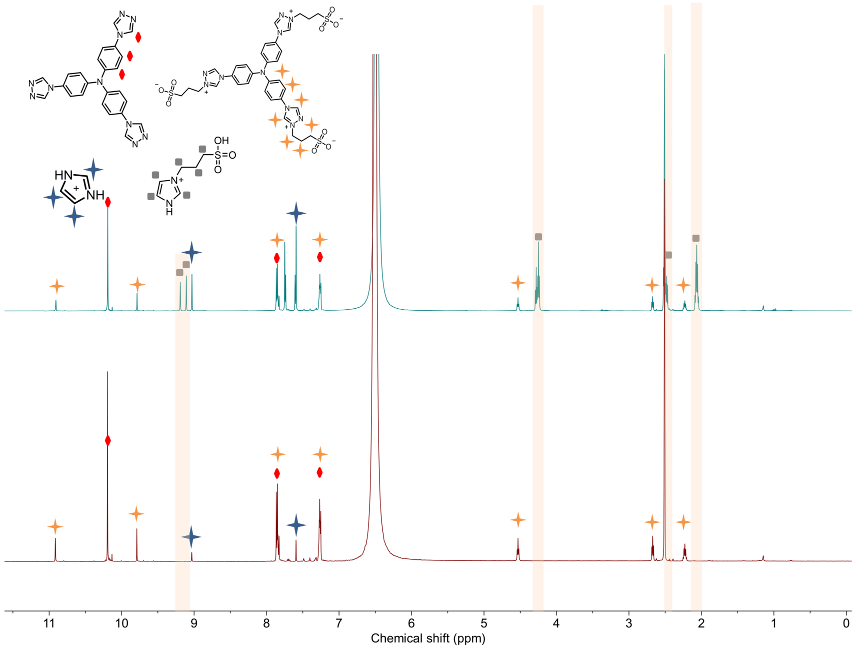

Supplementary Fig. 75: Comparison of the ^{1}H NMR spectra of a control mixture (protonated imidazole, sulfonated imidazolium, TTPA, and TTPA-S) with that of digested SiW-POF2(0.3)-S(60%) in DCl.

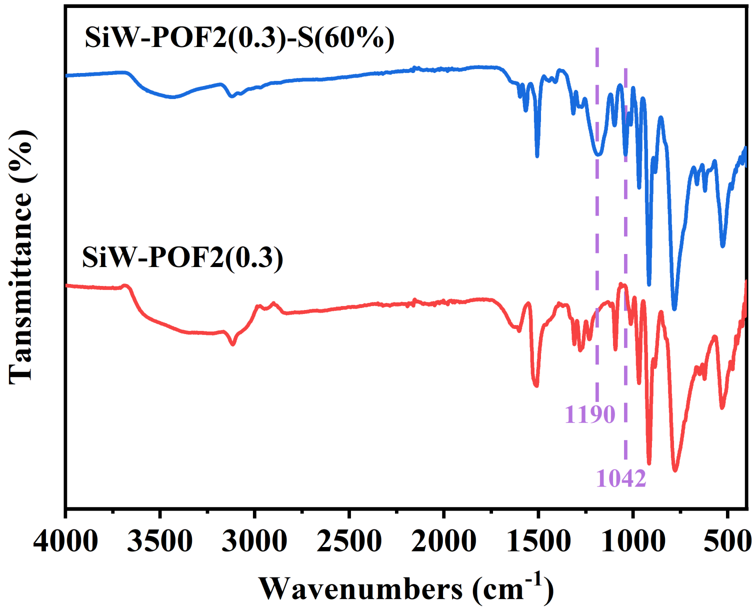

Supplementary Fig. 76: The FTIR spectrum of SiW-POF2(0.3) and SiW-POF2(0.3)-S(60%).

Thermogravimetric analysis (**Supplementary Fig. 77**) of SiW-POF2(0.3)-S(17%), SiW-POF2(0.3)-S(33%), SiW-POF2(0.3)-S(50%) and SiW-POF2(0.3)-S(60%) were carried under N_2 without pre-activation of any of the compounds. All four compounds followed a similar trend and lost ~1.5% of their initial weight within 150 °C. Absorbed volatile molecules were evaporated in this region. The process of 150-310 °C is mainly the evaporation of imidazole molecules. Starting from 310 °C, the slope of the sample is steeper with the increase of sulfonation ratio. This may be due to the simultaneous oxidation of the sample ligands TTPA and TTPA-S. Complete disintegration of SiW-POF2-S framework takes place around 800 °C. At higher temperatures, the residual compounds are partially converted into oxides. Therefore, about 70 % of the residual mass of the three compounds is composed of $\{SiW_{12}\}$ and tungsten oxide.

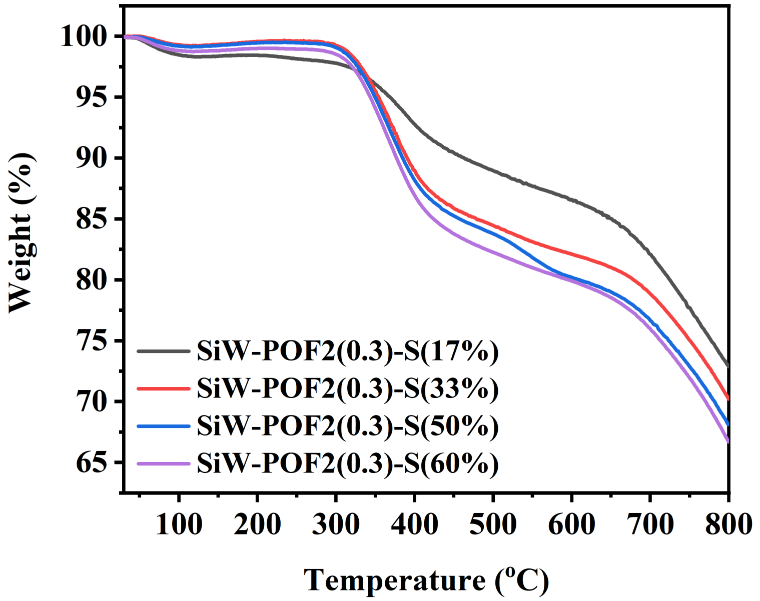

Supplementary Fig. 77: The TGA curve of SiW-POF2(0.3)-S(17/33/50/60%).

Supplementary Table 15: Mass percents of materials determined by elemental analysis measurements

Material	C (wt %)	H (wt %)	N (wt %)	S (wt %)
SiW-POF2(0.3)-S(17%)	17.30	2.10	7.20	0.7
SiW-POF2(0.3)-S(33%)	19.76	2.20	7.13	1.45
SiW-POF2(0.3)-S(50%)	22.30	2.34	7.15	2.18
SiW-POF2(0.3)-S(60%)	23.42	2.45	7.11	2.60

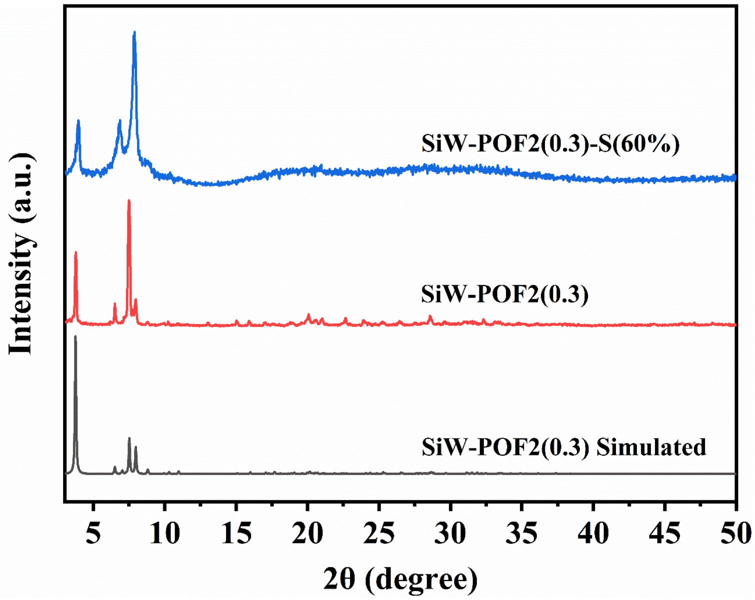

Supplementary Fig. 78: The PXRD patterns of SiW-POF2(0.3) and SiW-POF2(0.3)-S(60%).

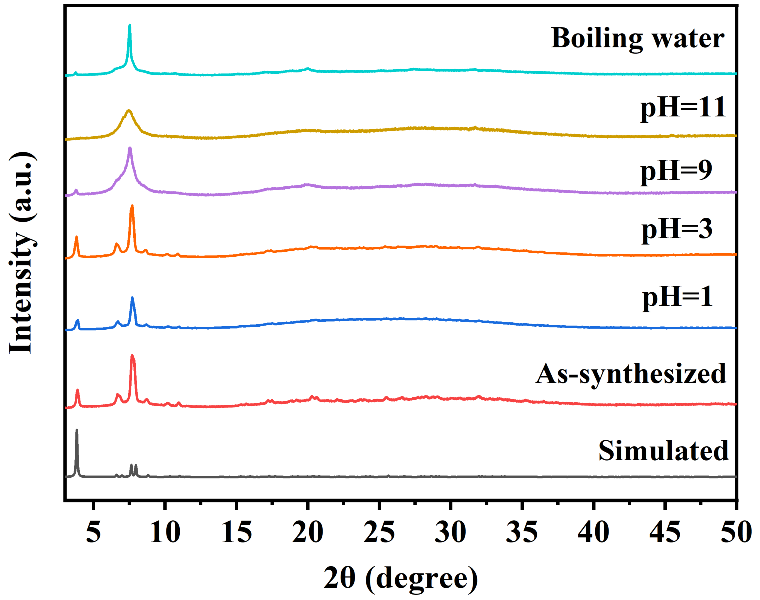

Supplementary Fig. 79: The PXRD patterns of SiW-POF2(0.3)-S(60%) in aqueous solution with pH ranging from 1 to11 for 24 h.

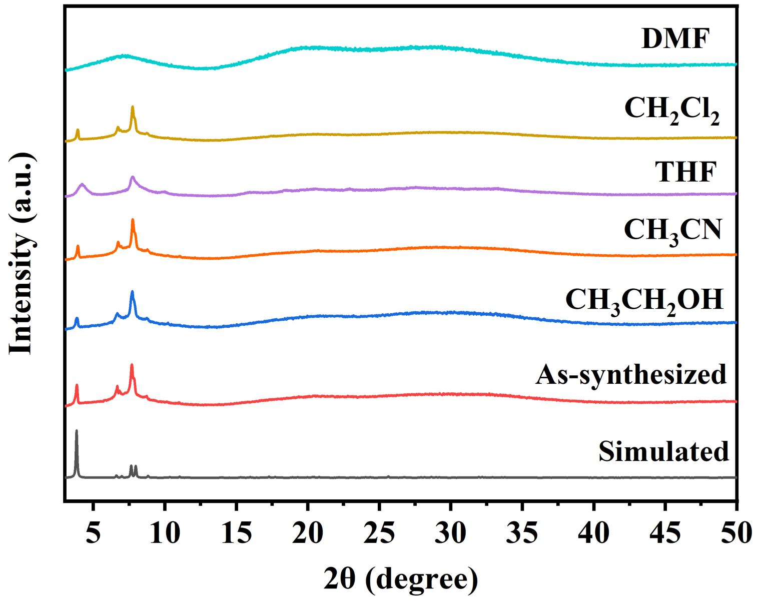

Supplementary Fig. 80: The PXRD patterns of SiW-POF2(0.3)-S(60%) in various organic solvents for 24 h.

4. Proton Conductivity Experiment of POF-S

The experimental conditions and test instruments are the same as **Supplementary Information Section 4**.

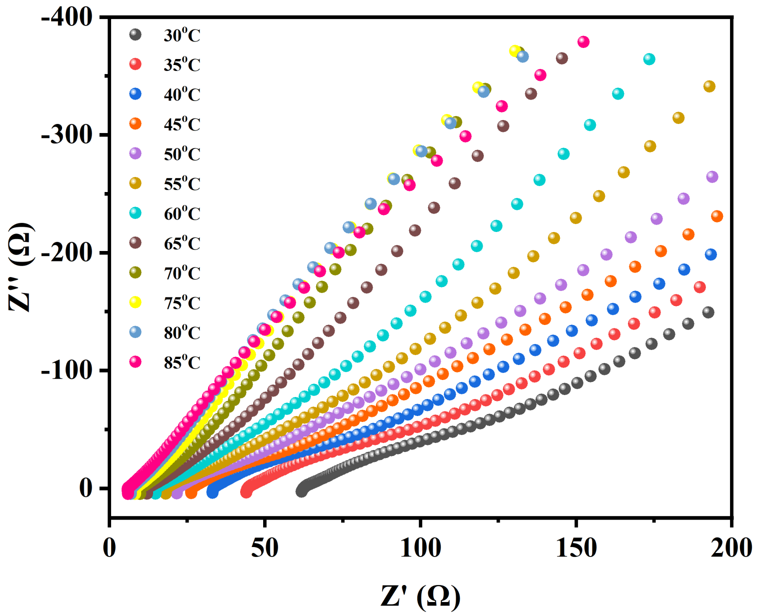

Supplementary Fig. 81: Impedance spectra of SiW-POF2(0.3)-S(60%) under 98% RH with different temperatures.

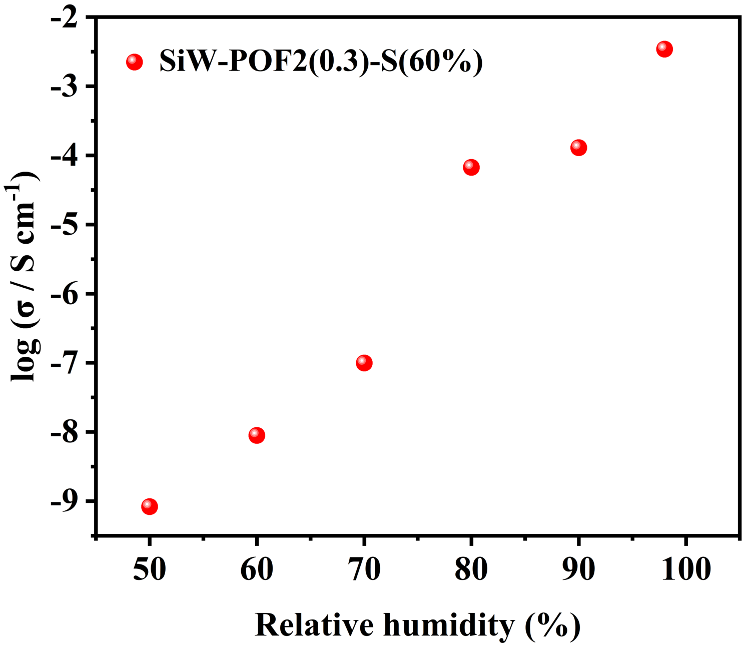

Supplementary Fig. 82: Nyquist plots of SiW-POF2(0.3)-S(60%) at various RH.

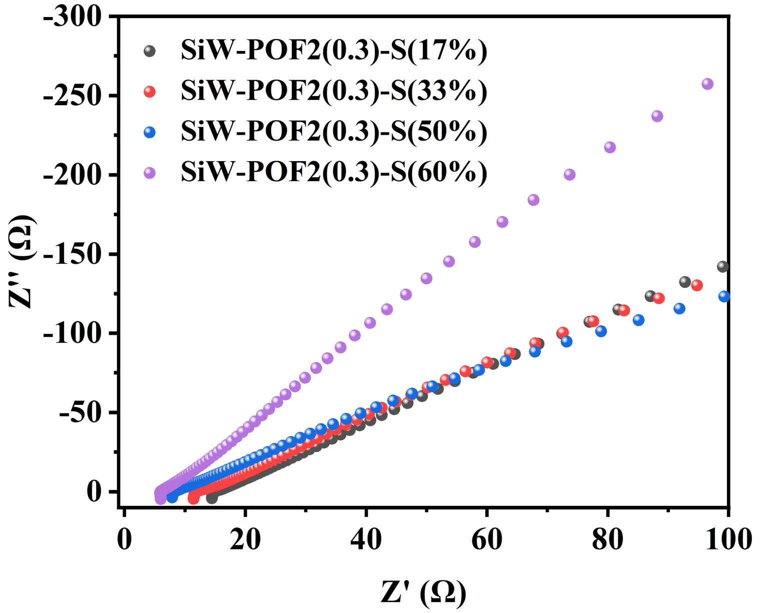

Supplementary Fig. 83: Comparison of impedance spectra for SiW-POF2(0.3)-S(17%), SiW-POF2(0.3)-S(33%), SiW-POF2(0.3)-S(50%) and SiW-POF2(0.3)-S(60%) under 85 °C with 98% RH.

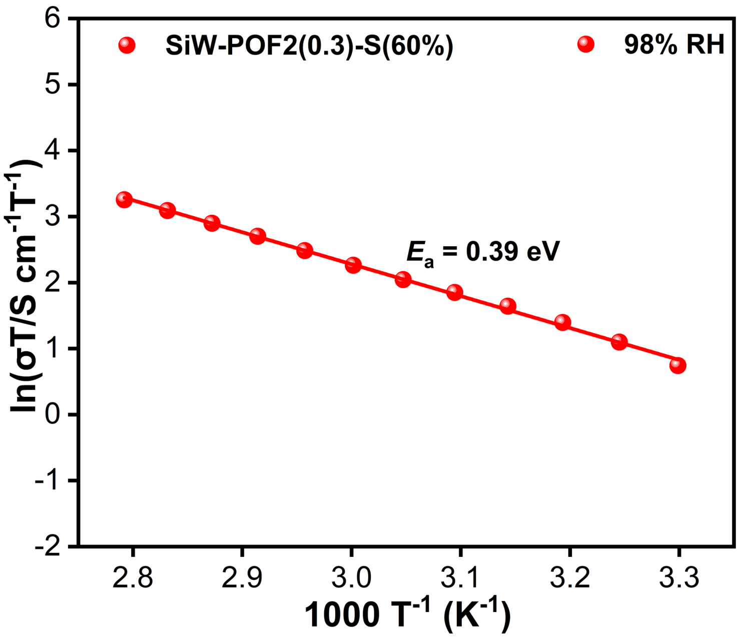

Supplementary Fig. 84: Arrhenius plot of proton conductivity for SiW-POF2(0.3)-S(60%) under 98% RH.

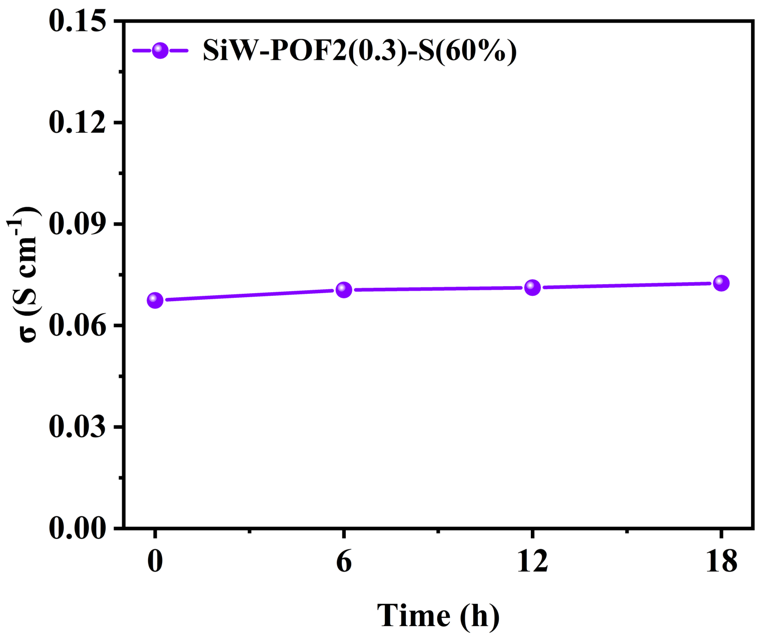

Supplementary Fig. 85: Time-dependent proton conductivity.

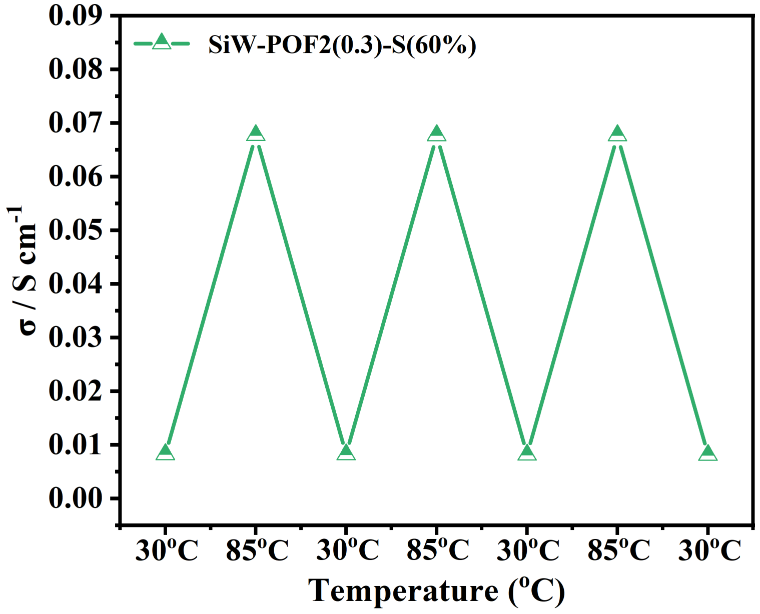

Supplementary Fig. 86: The stability test of proton conductivity of SiW-POF2(0.3)-S(60%) after three cycles of heating and cooling.

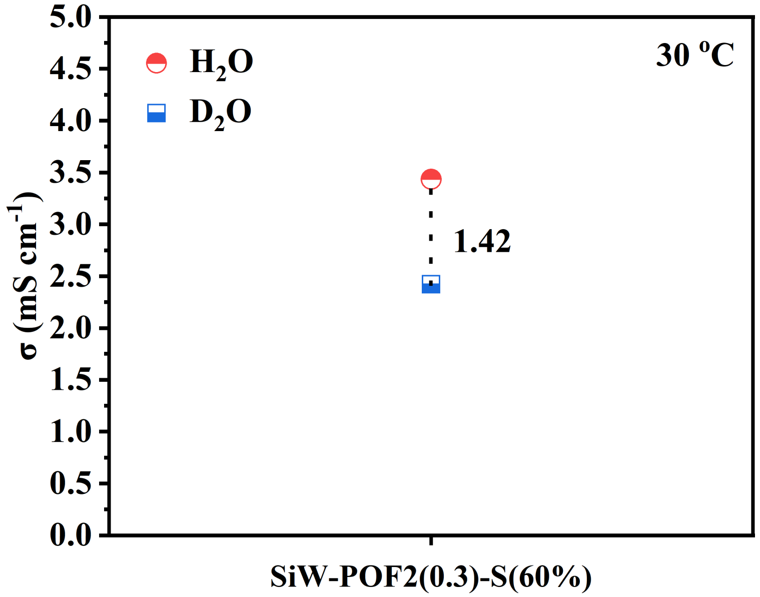

Supplementary Fig. 87: Proton conductivities of SiW-POF2(0.3)-S(60%) under H_2O or D_2O vapor atmosphere at 30 °C and 98% RH.

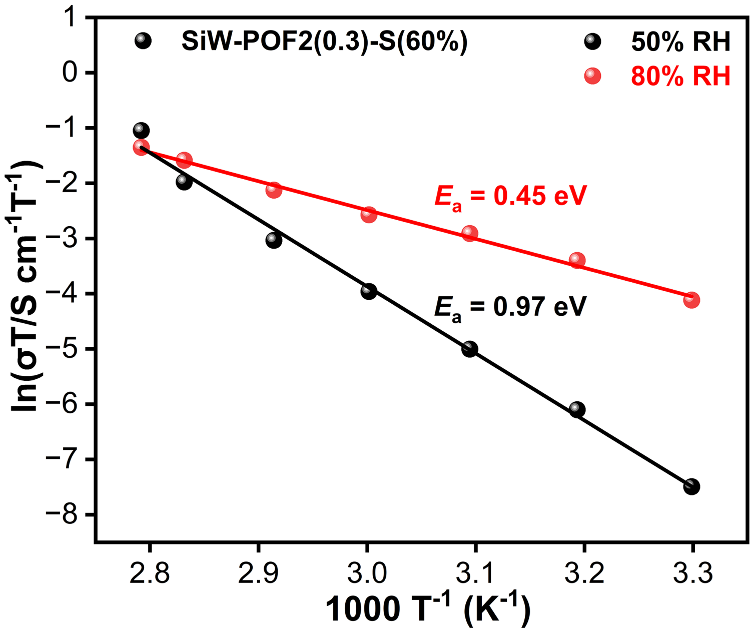

Supplementary Fig. 88: Arrhenius plots of proton conductivity for SiW-POF2(0.3)-S(60%) under 80% and 50% RH conditions.

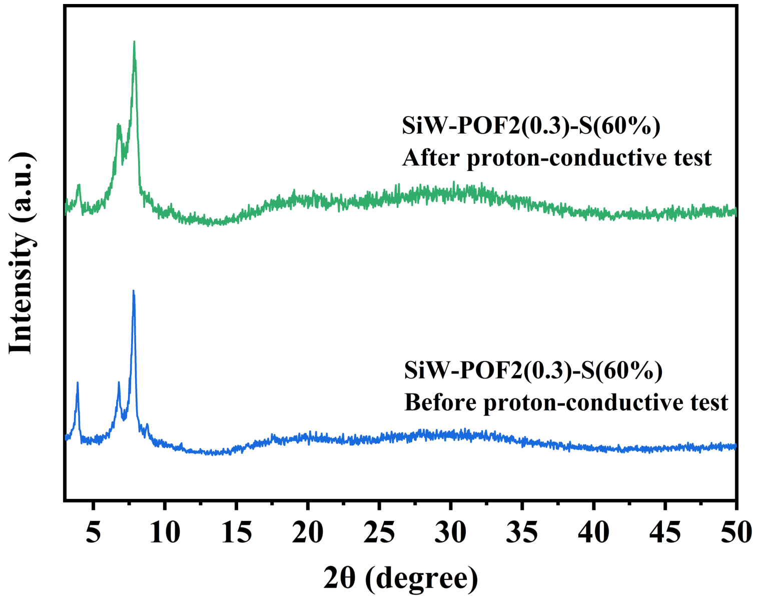

Supplementary Fig. 89: The PXRD patterns of SiW-POF2(0.3)-S(60%), before the proton-conductive test (blue) and after the proton-conductive test (green).

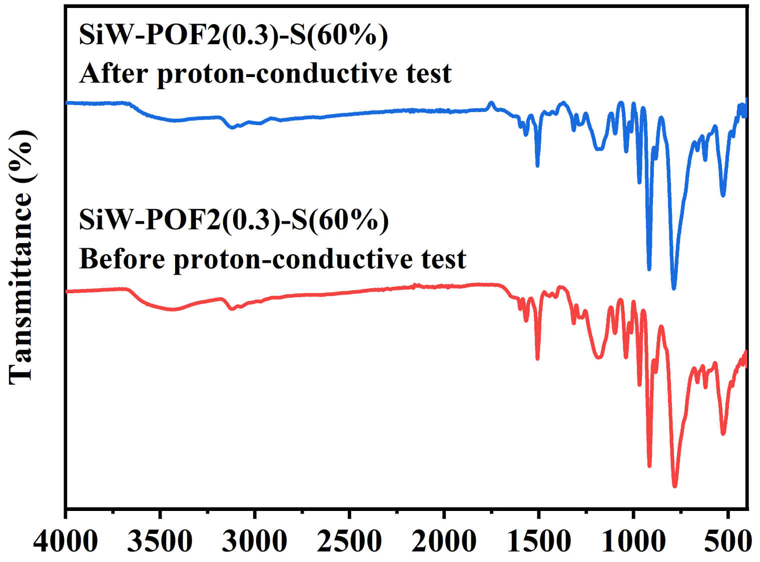

Supplementary Fig. 90: FTIR spectra of **SiW-POF2(0.3)-S(60%)** before and after the proton-conductivity test.

Supplementary Table. 16: Comparison of the proton conductivities of representative organic-inorganic hybrid POM-based crystalline materials with SiW-POF(0.3)-S(60%)

Compounds	$\sigma_{Proton\ conductivity}$ (S cm^{-1})	Relative humidity	Temperature (°C)	Reference
$NaH_{15}\{[P_2W_{15}Nb_3O_{62}]_2(4PBA)_2((4PBA)_2O)\}\cdot 53H_2O$	7.78×10^{-2}	98%	90	55
DETA-HPW@MIL-101-SO_3H	6.4×10^{-2}	95%	65	56
TETA-HPW@MIL-101-SO_3H	2.9×10^{-2}	95%	65	
$(HIm)_{24}(NH_4)_{20}[Mo_{72}{}^{VI}Mo_{60}{}^{V}O_{372}(CH_3COO)_{30}(H_2O)_{72}]\cdot$ ca190H_2O	4.98×10^{-2}	98%	60	57
$Co_3(Bip)_2(H_2O)_{10}(TeMo_6O_{24})$	2.96×10^{-2}	98%	90	58
SO_3H-IL-PMo_{12}@MIL-101	1.33×10^{-2}	70%	30	59
EB-COF:PW12	3.32×10^{-3}	97%	25	60
$\{[Cu(H_2bpdc)(H_2O)_{2.5}]_2[SiW_{12}O_{40}]\}\cdot 10H_2O$	1.77×10^{-3}	98%	100	61
$\{[Cu(debpdc)(H_2O)_3][Cu(debpdc)(H_2O)Cl][PMo_{12}O_{40}]\}\cdot 2CH_3OH\cdot 1.5CH_3CN\cdot 3H_2O$	4.71×10^{-5}	98%	100	
$H_9K[Cu(en)_2(H_2O)_5][Cu(en)_2]_4$-$[Eu(H_2O)_4]_3[Nb_{24}O_{69}(H_2O)_3]_2\cdot$ 2en$\cdot$ 45H_2O	1.50×10^{-3}	98%	85	62
$Na_5[H_7\{N(CH_2PO_3)_3\}Mo_6O_{16}(OH)(H_2O)_4]_4\cdot 18H_2O$	7.58×10^{-4}	98%	30	63
$[Ni(en)_3]_3\cdot[1,3\text{-dap}]_2\cdot H_6[V_{10}B_{26}O_{60}(OH)_{10}]$	3.0×10^{-4}	100%	25	64
$[PMo_{12}O_{40}][H_2PhI]_3[HPhI]\cdot 4H_2O$	2.2×10^{-4}	98%	100	65
$\{[Zn(dmphen)(DMF)_2(H_2O)]_2[SiW_{12}O_{40}]\}\cdot 6H_2O$	1.30×10^{-4}	98%	100	66
$[Zn_{12}(trz)_{20}][SiW_{12}O_{40}]\cdot 11H_2O$	1.2×10^{-4}	95%	95	67
$\{[Cu(4\text{-abpt})_2(H_2O)][Cu(4\text{-abpt})(H_2O)][SiW_{12}O_{40}]\}\cdot 6H_2O$	1.07×10^{-4}	98%	95	68
$[Mo_5P_2O_{23}]\ [Cu(phen)(H_2O)]_3\cdot 5H_2O$	2.2×10^{-5}	98%	25	69
$(NH_4)_5[Mo_8(OH)_2O_{24}(\mu_8\text{-}PO_4)](H_2O)_2$	2.13×10^{-5}	95%	25	70
$[Cu_3(\mu_3\text{-}OH)(H_2O)_3(atz)_3]_3[P_2W_{18}O_{62}]\cdot 14H_2O$	4.4×10^{-6}	97%	25	71
$[H_2en]_4[Ni_5(OH)_3(trzS)_3(en)(H_2O)(B\text{-}\alpha\text{-}PW_9O_{34})]\cdot 6H_2O$	4.0×10^{-7}	98%	25	72
**SiW-POF(0.3)-S(60%)**	**7.04×10^{-2}**	**98%**	**85**	**This work**

5. Gas and water vapor adsorption and dye uptake in solution

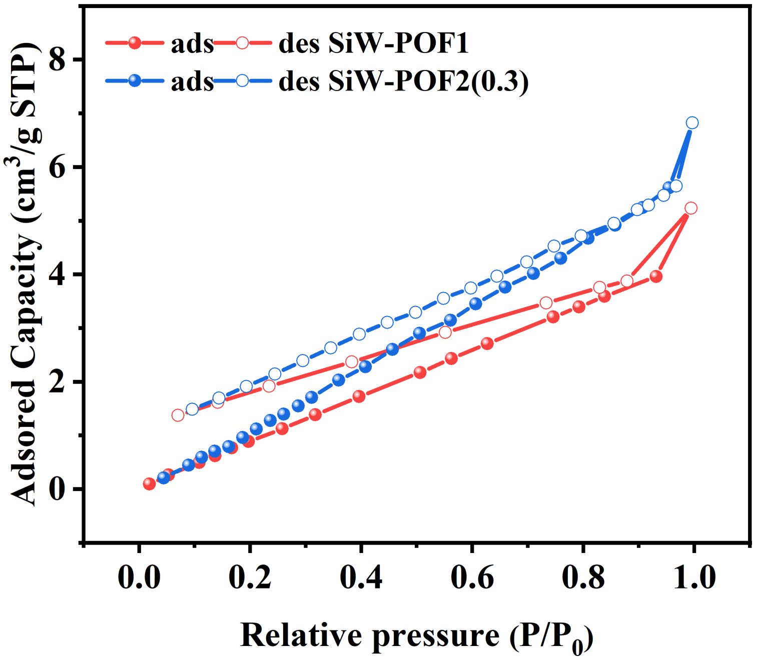

Supplementary Fig. 91: N_2 adsorption and desorption isotherms of SiW-POF1 and SiW-POF2(0.3) at 77 K.

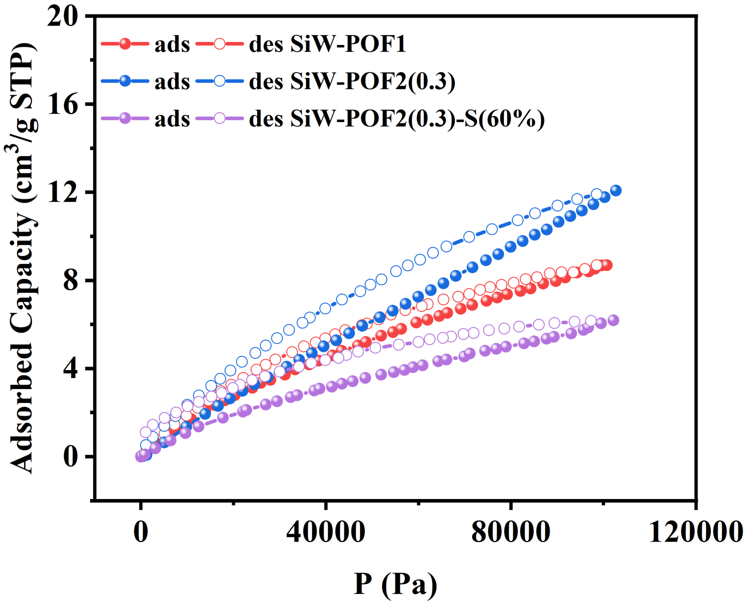

Supplementary Fig. 92: CO_2 adsorption and desorption isotherms of SiW-POF1, SiW-POF2(0.3) and SiW-POF2(0.3)-S(60%) at 298 K.

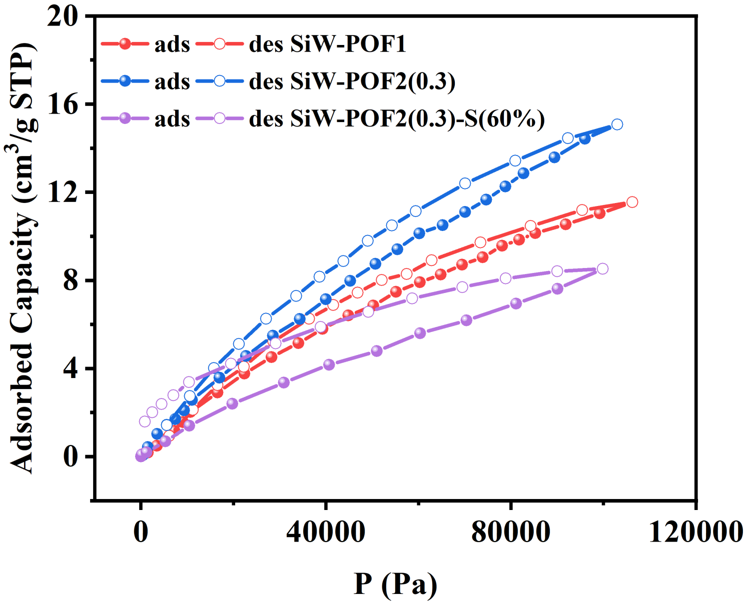

Supplementary Fig. 93: CO_2 adsorption and desorption isotherms of SiW-POF1, SiW-POF2(0.3) and SiW-POF2(0.3)-S(60%) at 273 K.

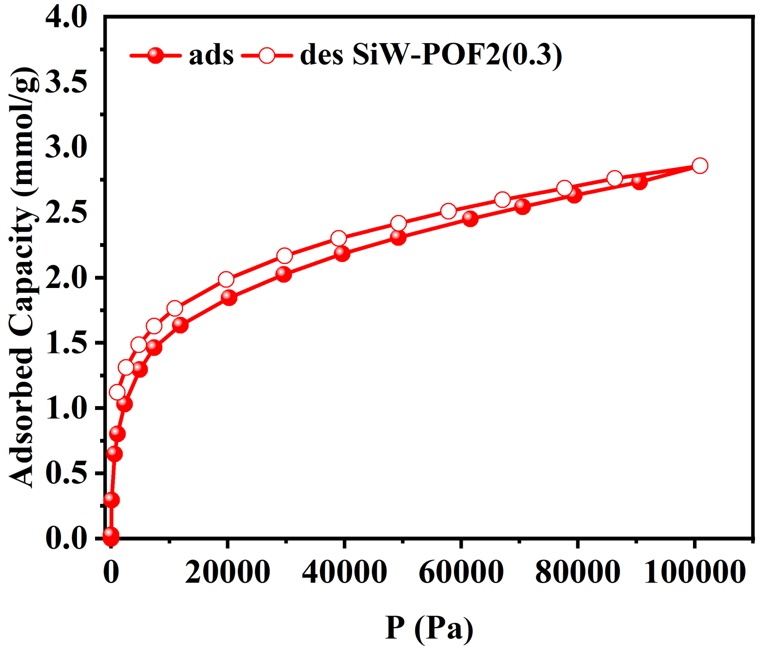

Supplementary Fig. 94: NH_3 adsorption and desorption isotherms of SiW-POF2(0.3) at 298 K.

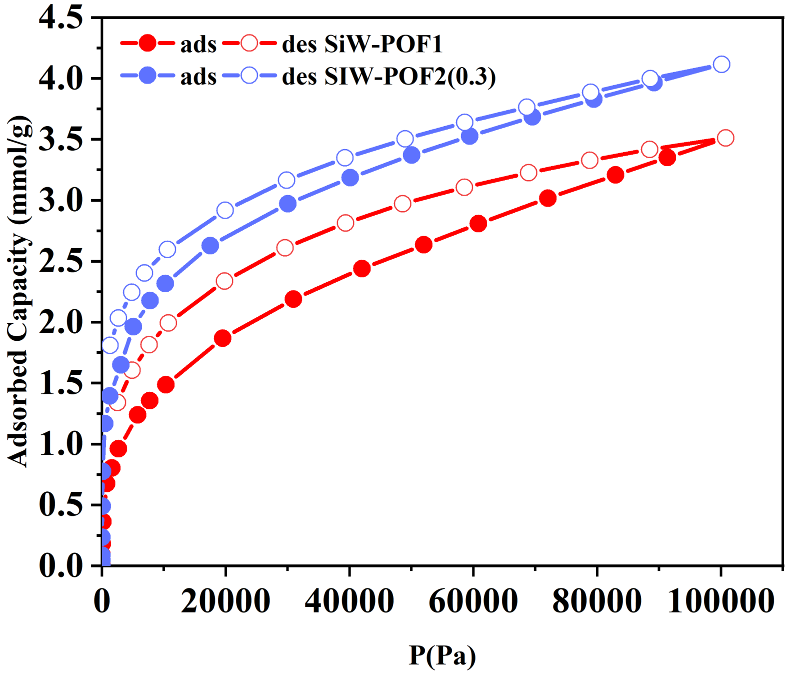

Supplementary Fig. 95: NH_3 adsorption and desorption isotherms of SiW-POF1 and SiW-POF2(0.3) at 273 K.

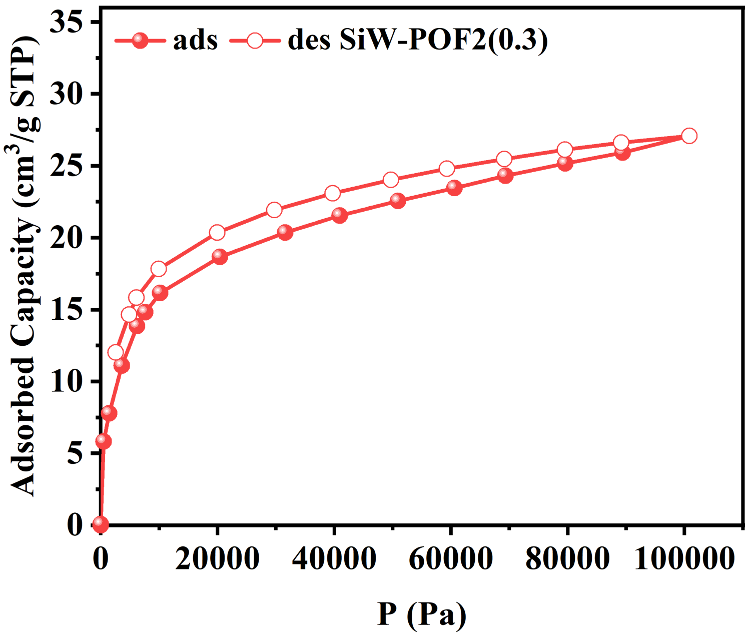

Supplementary Fig. 96: SO_2 adsorption and desorption isotherms of SiW-POF2(0.3) at 298 K.

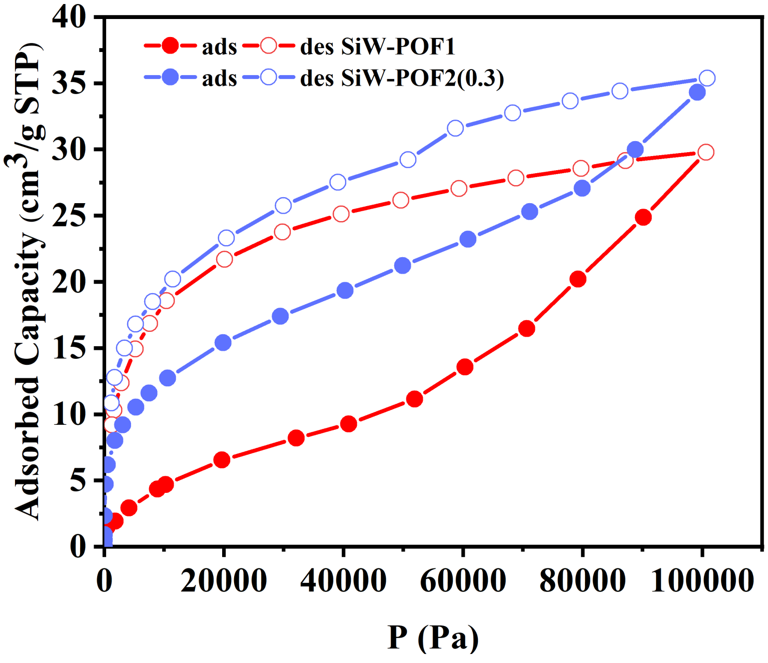

Supplementary Fig. 97: SO_2 adsorption and desorption isotherms of SiW-POF1 and SiW-POF2(0.3) at 273 K.

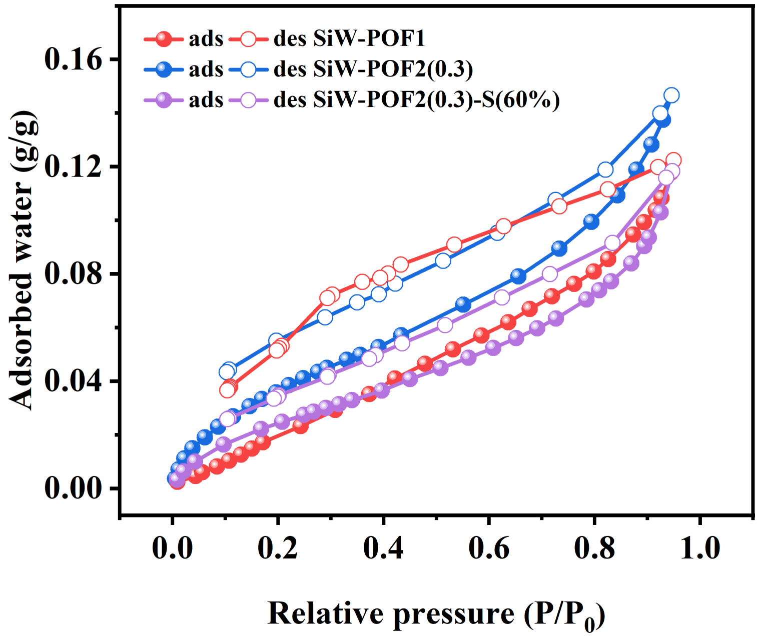

Supplementary Fig. 98: The water vapor adsorption and desorption isotherms of SiW-POF1, SiW-POF2(0.3) and SiW-POF2(0.3)-S(60%) at 25 °C.

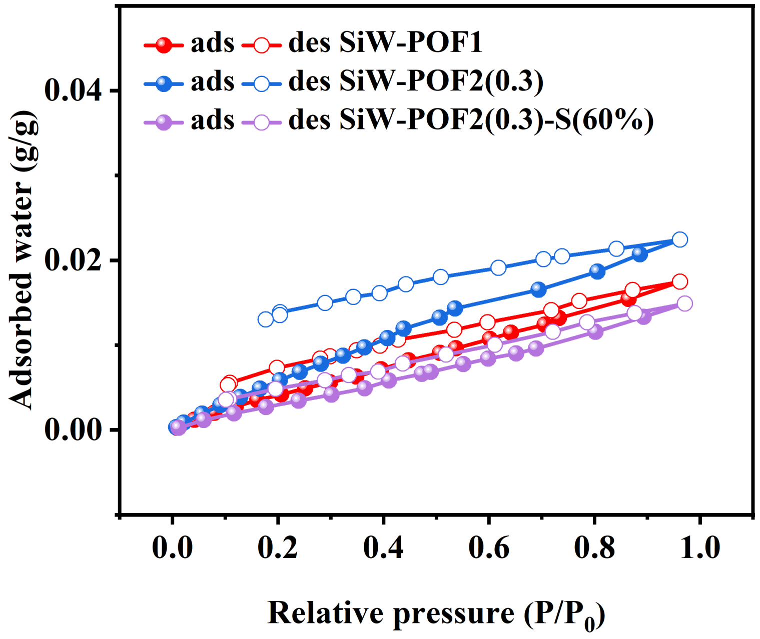

Supplementary Fig. 99: The water vapor adsorption and desorption isotherms of SiW-POF1, SiW-POF2(0.3) and SiW-POF2(0.3)-S(60%) at 80 °C.

Dye adsorption by SiW-POF1 and SiW-POF2(0.3)

Taking 3 mL of 20mg/L standard solution (DASP, trans-4-[4-(Dimethylamino)styryl]-1-methylpyridinium iodide) into a 50 mL glass bottle, 5 mg SiW-POF1/SiW-POF2(0.3) was subsequently added and stirred for 24 hours. The solution was then filtered and washed by water until no characteristic adsorption of DASP detected from the filtrate. The visualization of dye adsorption was directly observed by both optical imaging and confocal laser scanning microscopy (CMLS). The amount of absorbed dye per formula was evaluated by ^{1}H NMR via digesting SiW-POF1/SiW-POF2(0.3) sample by DCl.

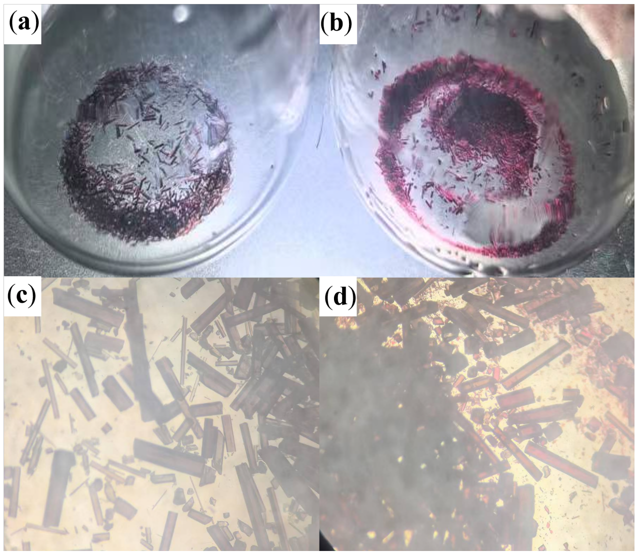

Supplementary Fig. 100: Optical images of SiW-POF2(0.3) crystals before (a) and after (b) DASP dye adsorption; magnified images of SiW-POF2(0.3) before (c) and after (d) DASP dye adsorption.

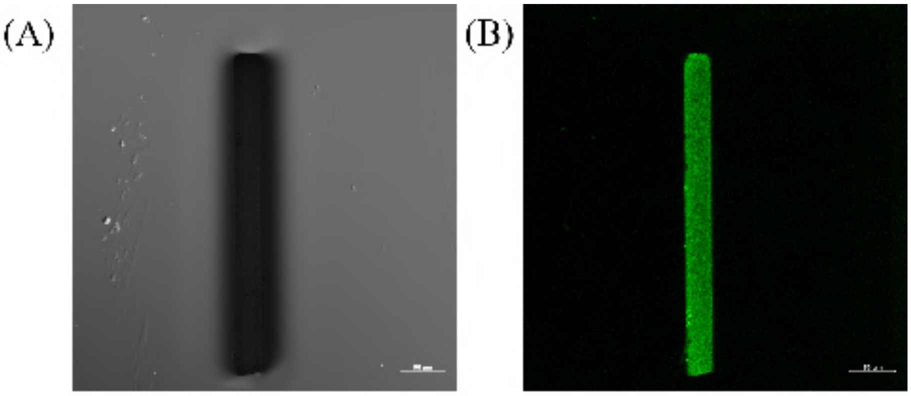

Supplementary Fig. 101: The CLSM images of SiW-POF2(0.3) before and after adsorption of DASP.

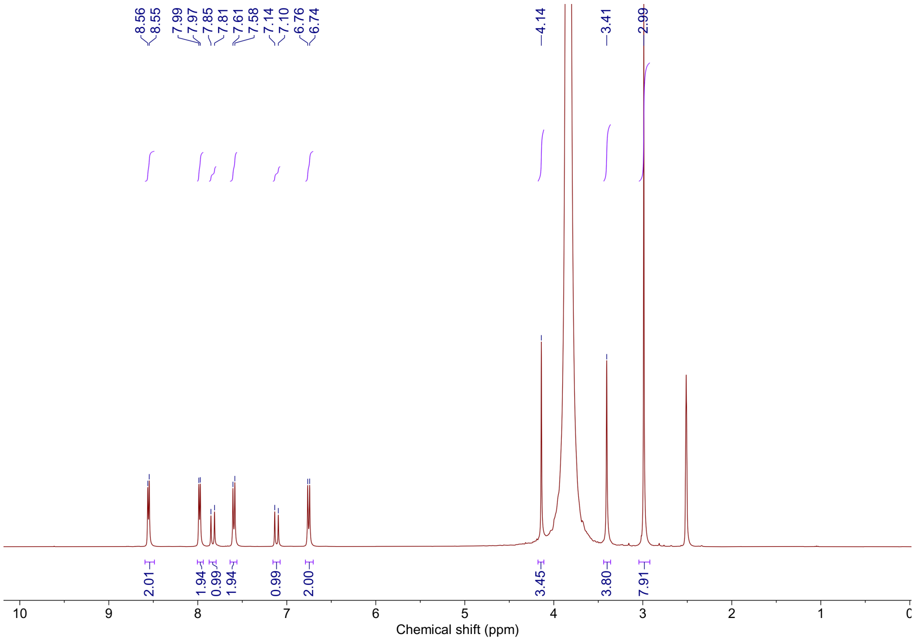

Supplementary Fig. 102: ^{1}H NMR spectrum of DASP.

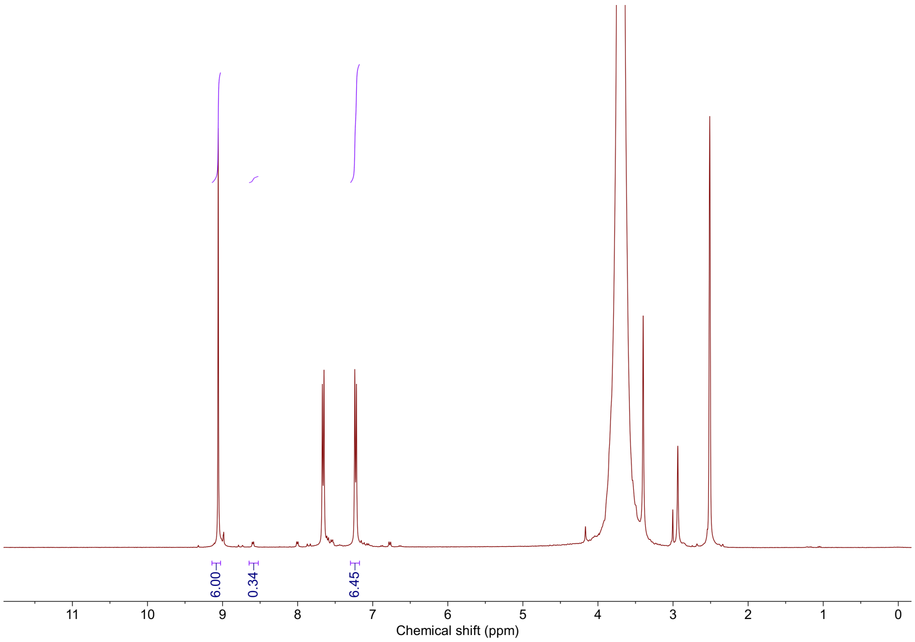

Supplementary Fig. 103: ^{1}H NMR spectrum of digested DASP@SiW-POF1 in DCl, indicating 17% adsorption.

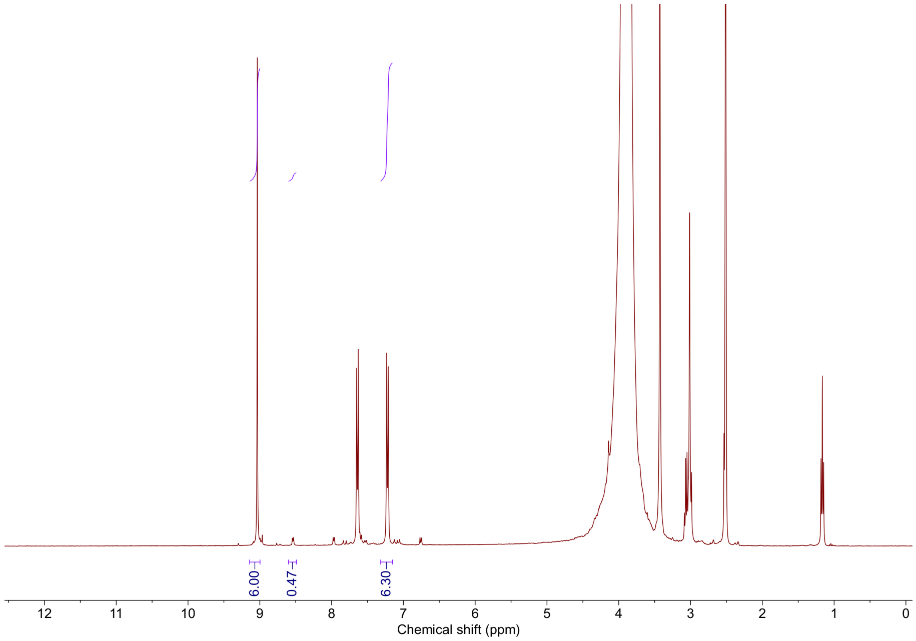

Supplementary Fig. 104: ^{1}H NMR spectrum of digested DASP@SiW-POF2(0.3) in DCl, indicating 23.5% adsorption.

6. Solid State NMR

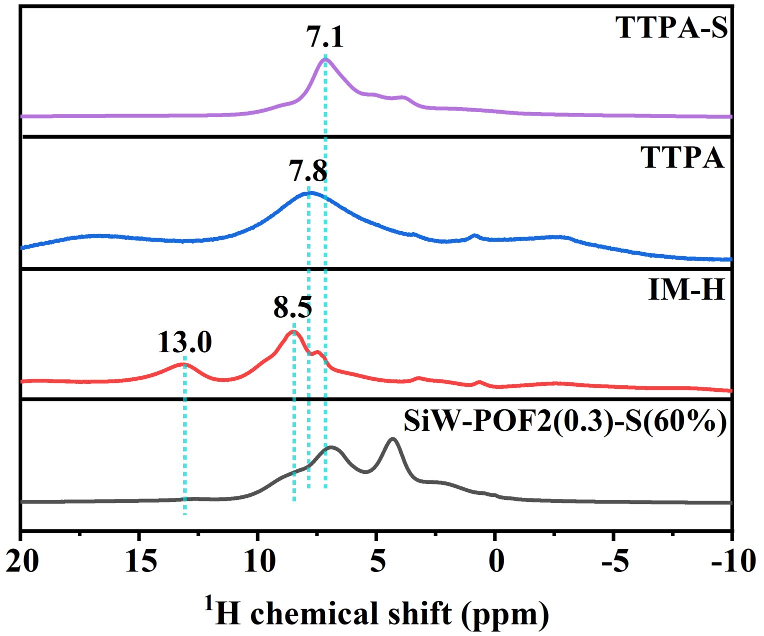

Supplementary Fig. 105: ^{1}H MAS NMR spectra of TTPA-S/H_4SiW_{12}, TTPA-S, TTPA, imidazolium (IM-H) and SiW-POF2-S(60%).

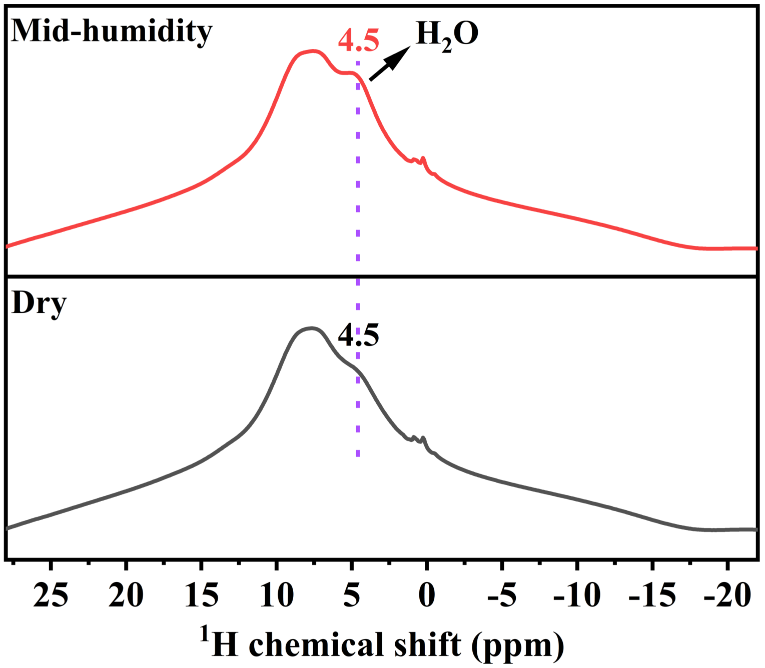

Supplementary Fig. 106: ^{1}H MAS NMR spectra of SiW-POF2(0.3)-S(60%) at different humidity.

7. Conductivity (σ) values of the as-synthesized compounds

Supplementary Table 17: Conductivity (σ) values of the as-synthesized compounds

**Compounds**	**Condition (Temp., RH)**	**σ (S cm^{-1})**
SiW-POF1	30 °C, 50%	3.28×10^{-5}
SiW-POF1	30 °C, 60%	5.96×10^{-5}
SiW-POF1	30 °C, 70%	7.94×10^{-5}
SiW-POF1	30 °C, 80%	1.00×10^{-4}
SiW-POF1	30 °C, 90%	1.61×10^{-4}
SiW-POF1	30 °C, 98%	2.74×10^{-4}
SiW-POF1	85 °C, 98%	1.11×10^{-3}
SiW-POF3	85 °C, 98%	9.18×10^{-4}
BW-POF1	85 °C, 98%	1.28×10^{-3}
BW-POF3	85 °C, 98%	1.07×10^{-3}
PW-POF3	85 °C, 98%	7.50×10^{-4}

Supplementary Table 18: Conductivity (σ) values of the as-synthesized compounds

Compounds	Condition (Temp., RH)	σ (S cm^{-1})
SiW-POF2(0.1)	85 ºC, 98%	3.12×10^{-3}
SiW-POF2(0.3)	85 ºC, 98%	1.00×10^{-2}
SiW-POF2(0.5)	85 ºC, 98%	3.72×10^{-3}
SiW-POF2(1.3)	85 ºC, 98%	3.70×10^{-3}
SiW-POF2(2.0)	85 ºC, 98%	2.09×10^{-3}
PW-POF2(0.3)	85 ºC, 98%	9.82×10^{-3}
BW-POF2(0.3)	85 ºC, 98%	1.00×10^{-2}
BW-POF2(1.3)	85 ºC, 98%	1.16×10^{-3}

Supplementary Table 19: Conductivity (σ) values of the Sulfonic acid post-modified compounds

Compounds	Condition (Temp., RH)	σ (S cm^{-1})
SiW-POF1-S(28%)	85 ºC, 98%	1.35×10^{-2}
SiW-POF2(0.3)-S(17%)	85 ºC, 98%	2.57×10^{-2}
SiW-POF2(0.3)-S(33%)	85 ºC, 98%	3.56×10^{-2}
SiW-POF2(0.3)-S(50%)	85 ºC, 98%	5.55×10^{-2}
SiW-POF2(0.3)-S(60%)	85 ºC, 98%	7.04×10^{-2}

Supplementary Table 20: Comparison of the proton conductivity (σ) values of the SiW-POF series

Compounds	Condition (Temperature, RH)	σ (S cm^{-1})
SiW-POF2	85 ºC, 98%	1.00×10^{-2}
SiW-POF1	85 ºC, 98%	1.11×10^{-3}
SiW-POF3	85 ºC, 98%	9.18×10^{-4}

8. Fabrication and structural characterization of SiW-POF2-S(60%)@Nafion hybrid membranes

The SiW-POF2(0.3)-S(60%)@Nafion hybrid membranes with loadings of 1%, 2%, 3%, 4%, 5%, and 7.5% were prepared by dispersing 1.7 mg, 3.3 mg, 5 mg, 6.8mg, 8.3 mg, and 12.5 mg sample of SiW-POF2(0.3)-S(60%), respectively, in a mixture of DMF (0.7 mL) and 5 wt% Nafion solution (3.5 g). Then the mixtures were sonicated for 1 h and stirred overnight at room temperature to obtain homogeneous solutions. Subsequently, the resultant solutions were poured onto 4 × 4 cm glass plates and dried under vacuum at 100 ℃ for 12 h to afford the corresponding hybrid membranes.

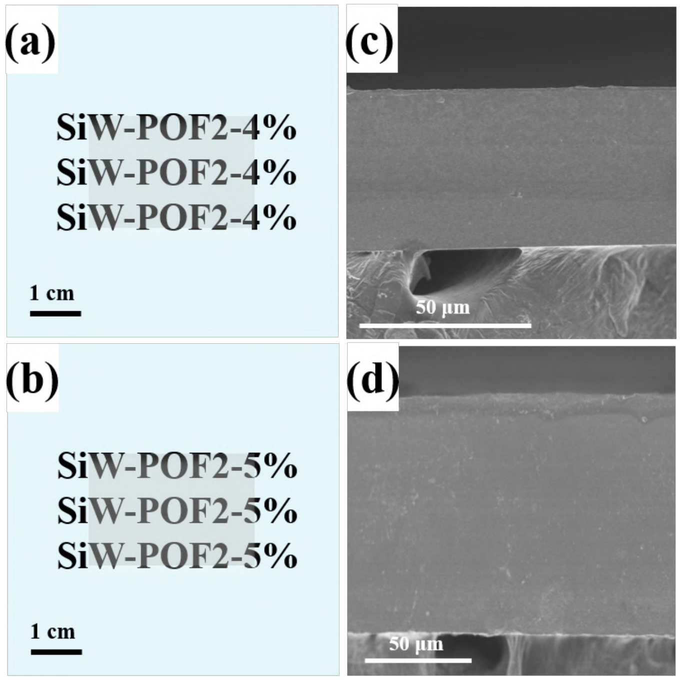

Supplementary Fig. 107: Photographs of hybrid SiW-POF2-S(60%)@Nafion-4% and -5% membranes (a,b); Cross-sectional SEM images of hybrid SiW-POF2-S(60%)@Nafion-4% and -5% membranes (c,d).

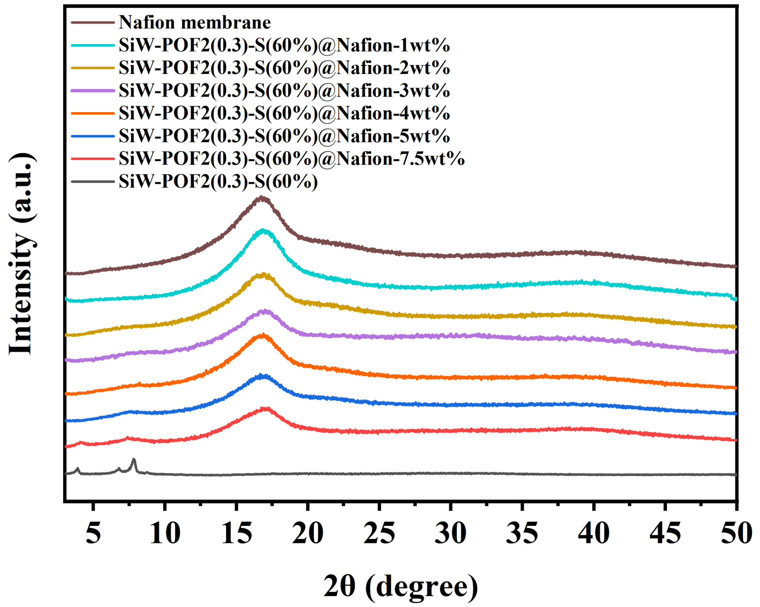

Supplementary Fig. 108: Experimental PXRD patterns of pristine Nafion membrane and SiW-POF2(0.3)–S(60%)@Nafion hybrid membranes (1-7.5%) alongside the simulated PXRD pattern of SiW-POF2(0.3)–S(60%)

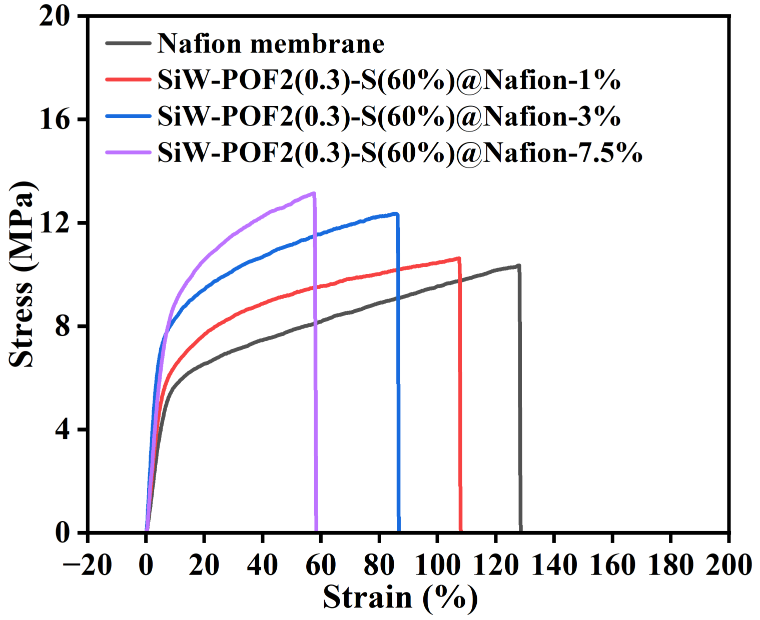

Supplementary Fig. 109: Stress-strain curves for the Nafion and SiW-POF2(0.3)-S(60%)@Nafion hybrid membranes.

Supplementary Table 21: Mechanical properties of Nafion and SiW-POF2(0.3)-S(60%)@Nafion hybrid membranes,

Samples	Tensile strength (MPa)	Elongation at break (%)
Nafion membrane	10.3	128
SiW-POF2(0.3)-S(60%)@Nafion-1%	10.6	107
SiW-POF2(0.3)-S(60%)@Nafion-3%	12.3	86
SiW-POF2(0.3)-S(60%)@Nafion-7.5%	13.1	57

Supplementary Fig. 109 and **Supplementary Table 21** show the stress-strain curve for pristine Nafion with a breaking strength of 10.3 MPa and an elongation of 128%. In comparison, the 1 and 3 wt.% hybrid membranes exhibit elongations of 107% and 86%, respectively, with breaking strengths increasing to 10.6 and 12.3 MPa. Furthermore, the new 7.5 wt.% hybrid membrane shows a much longer elongation of 57%, which is a substantial increase over the 10% observed in the previous samples of the original manuscript. This is despite the heterogeneity found in this overloaded membrane. Overall, these results indicate that the addition of the POF filler increases the breaking strength due to a reinforcement effect, while reducing slightly the elongation at break. This transition – where the breaking strength of the material increases upon the addition of a crystalline filler – is a common phenomenon in composite science and does not

represent a degradation of mechanical robustness. On the contrary, the increased breaking strength suggests that the POF nanoparticles effectively reinforce the Nafion matrix.

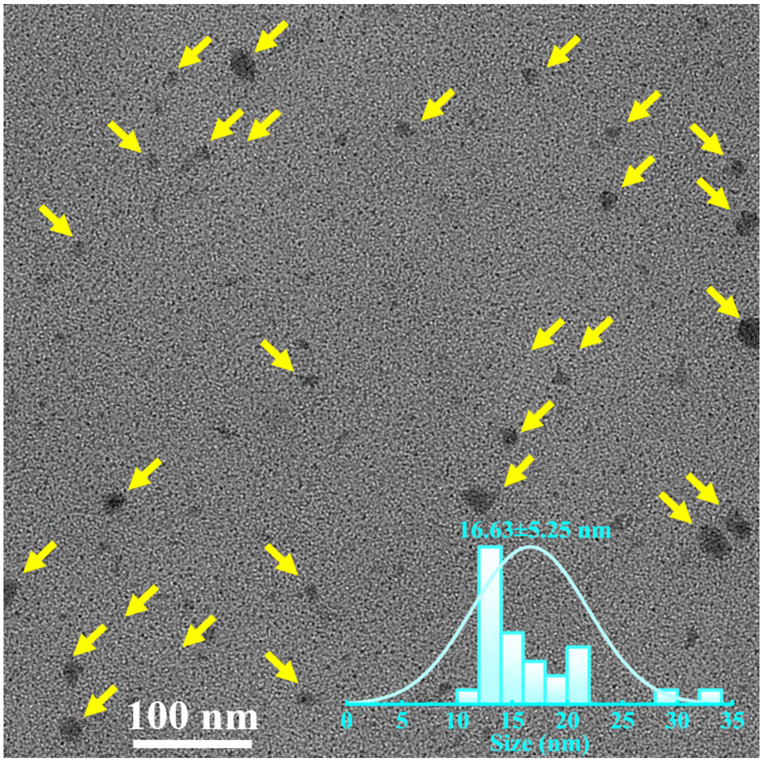

Supplementary Fig. 110: HRTEM image of the SiW-POF2(0.3)–S(60%)@Nafion-3% hybrid membrane with the corresponding particle size distribution shown in the inset.

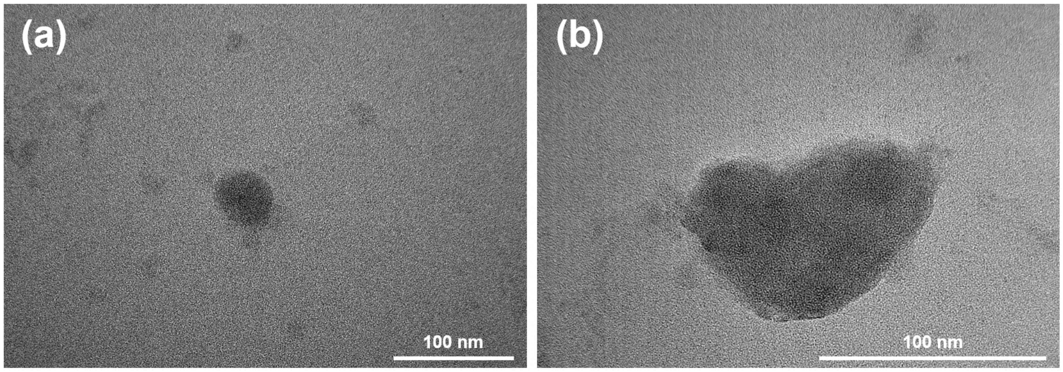

Supplementary Fig. 111: HRTEM images of larger SiW-POF2-S(60%) nanoparticles with size of 50nm-80nm in 3% hybrid membrane.

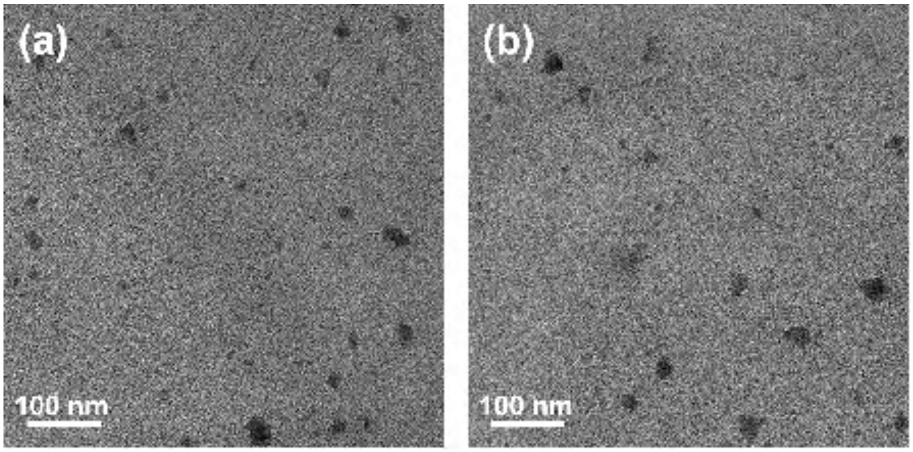

Supplementary Fig. 112: HRTEM images of ultrathin sections of SiW-POF2-S(60%)@Nafion-4% (a) and SiW-POF2-S(60%)@Nafion-5% (b).

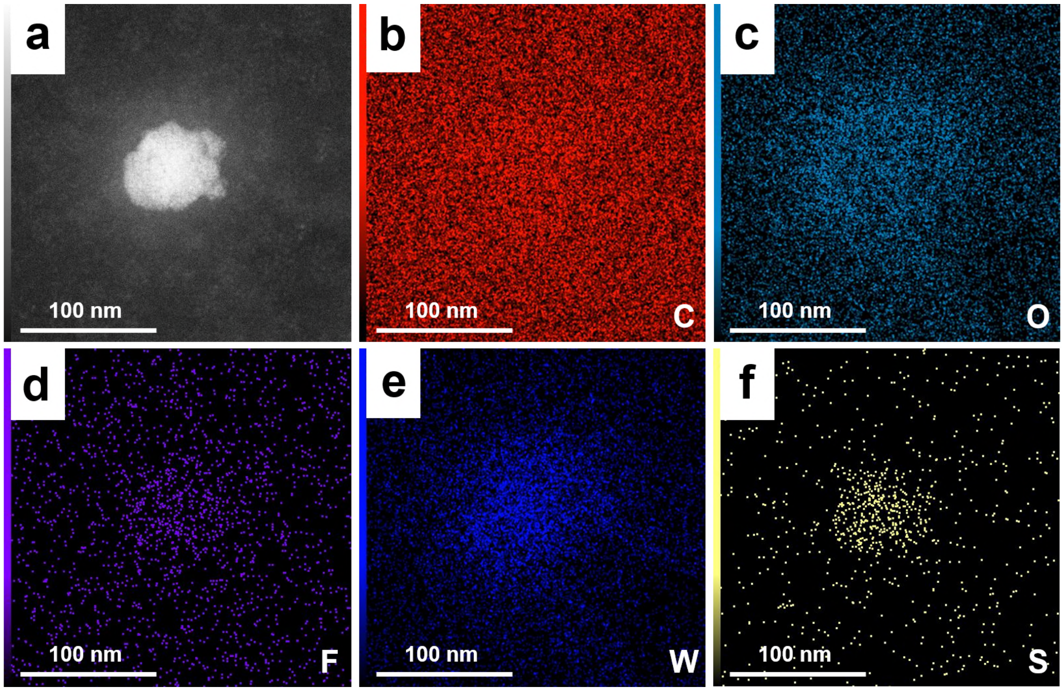

Supplementary Fig. 113: (a) HAADF-STEM image of a SiW-POF2-S(60%) nanoparticle and surrounding area in the 3% hybrid membrane. **(b-f)** EDX mapping of C, O, F, W and S for the nanoparticle and surrounding area.

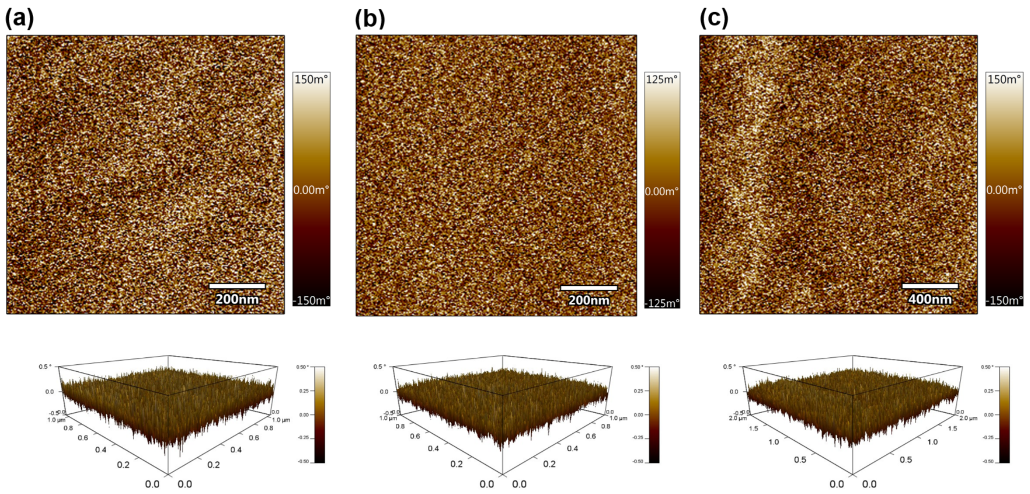

Supplementary Fig. 114: (a) Tapping mode AFM phase images and topography images for Nafion membrane (a), SiW-POF2-S(60%)@Nafion-1% (b) and SiW-POF2-S(60%)@Nafion-3% (c).

.

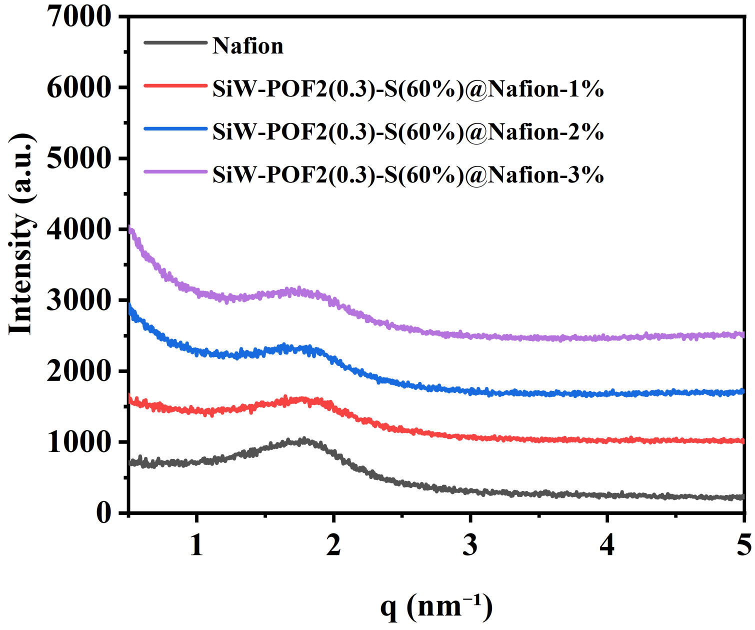

Supplementary Fig. 115: SAXS profiles of Nafion, SiW-POF2-S(60%)@Nafion-1%, SiW-POF2-S(60%)@Nafion-2%, SiW-POF2-S(60%)@Nafion-3%.

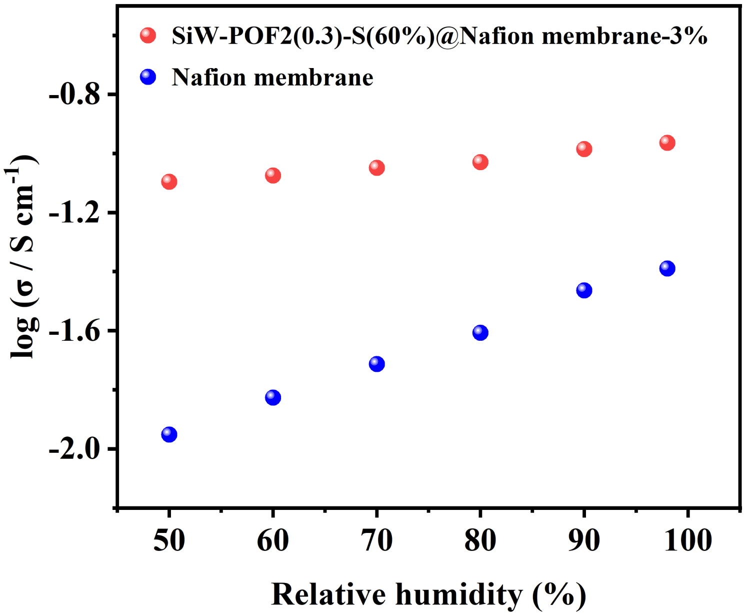

Supplementary Fig. 116: Nyquist plots of Nafion and SiW-POF2(0.3)-S(60%)@Nafion-3% membrane at 30 °C under various RH.

Supplementary Table 22: Conductivity (σ) values of Nafion and SiW-POF2-S(60%)@Nafion membranes.

**Membranes**	**Condition (Temperature, RH)**	**σ (S cm^{-1})**
Nafion	30 °C, 98%	4.53×10^{-2}
SiW-POF2-S(60%)@Nafion-1%	30 °C, 98%	8.97×10^{-2}
SiW-POF2-S(60%)@Nafion-2%	30 °C, 98%	9.13×10^{-2}
SiW-POF2-S(60%)@Nafion-3%	30 °C, 98%	1.085×10^{-1}
SiW-POF2-S(60%)@Nafion-4%	30 °C, 98%	9.14×10^{-2}
SiW-POF2-S(60%)@Nafion-5%	30 °C, 98%	8.83×10^{-2}
SiW-POF2-S(60%)@Nafion-7.5%	30 °C, 98%	7.59×10^{-2}
Nafion	85 °C, 98%	1.04×10^{-1}
SiW-POF2-S(60%)@Nafion-1%	85 °C, 98%	1.42×10^{-1}
SiW-POF2-S(60%)@Nafion-2%	85 °C, 98%	1.54×10^{-1}
SiW-POF2-S(60%)@Nafion-3%	85 °C, 98%	1.91×10^{-1}
SiW-POF2-S(60%)@Nafion-4%	85 °C, 98%	1.56×10^{-1}
SiW-POF2-S(60%)@Nafion-5%	85 °C, 98%	1.47×10^{-1}
SiW-POF2-S(60%)@Nafion-7.5%	85 °C, 98%	1.41×10^{-1}

Supplementary Table 23: Comparison of the conductivity (σ) values of Nafion, SiW-POF2-S(60%)@Nafion-3% and hybrid membranes constructed from the components of SiW-POF2-S(60%) and Nafion

Membranes	Condition (Temperature, RH)	σ (S cm^{-1})
Nafion	85 °C, 98%	1.04×10^{-1}
TTPA-S@Nafion-3%	85 °C, 98%	1.29×10^{-1}
SiW_{12}@Nafion-3%	85 °C, 98%	1.37×10^{-1}
SiW_{12}/TTPA-S @Nafion-3%	85 °C, 98%	1.53×10^{-1}
SiW-POF2-S(60%)@Nafion-3%	85 °C, 98%	1.97×10^{-1}

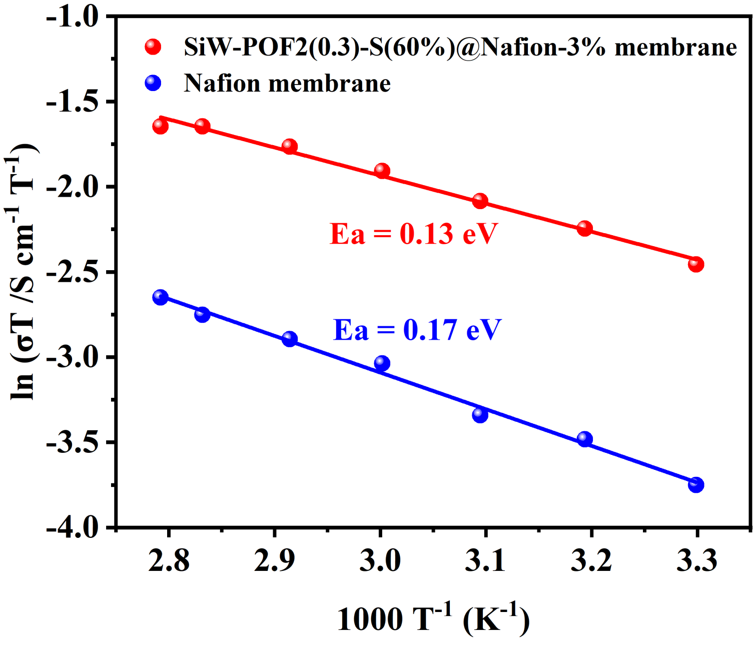

Supplementary Fig. 117: Arrhenius plots of proton conductivity for Nafion and SiW-POF2(0.3)-S(60%)@Nafion-3% membranes under 98% RH conditions.

Supplementary Table 24: Summary of the repeated proton conductivity measurements and statistical parameters for pure Nafion, SiW-POF2(0.3)-S(60%), and SiW-POF2(0.3)-S(60%)@Nafion-3% membrane.

Sample Name	Sample No.	Conductivity (S cm⁻¹)	No. of Tests (n)	Mean (S cm⁻¹)	Std. Dev. (S cm⁻¹)	RSD (%)*
SiW-POF2(0.3)-S(60%) (Pure Filler)	1	0.0705	3	0.0704	0.0010	1.40%
	2	0.0674				
	3	0.0725				
Nafion D520 (Reference)	1	0.1033	3	0.1042	0.0009	0.86%
	2	0.1050				
	3	0.1042				
SiW-POF2(0.3)-S(60%)@Nafion-3% (hybrid membrane)	1	0.1915	3	0.1910	0.0008	0.42%
	2	0.1915				
	3	0.1901				

Measurement Conditions: 98% RH, 80 °C.
*Note: Std. Dev. = Standard Deviation; RSD = Relative Standard Deviation (Std. Dev./Mean × 100%).

Supplementary Table. 25: Comparison of the proton conductivity of reported POM@Nafion composite membranes with SiW-POF2(0.3)-S(60%@Nafion-3%

Composite membrane	$\sigma_{composite-membrane}$ (S cm^{-1})	$\sigma_{pure-polymer}$ (S cm^{-1})	$\sigma_{composite-membrane}/\sigma_{pure-polymer}$	Reference
Nafion/PW-mGO	0.117	0.098	1.19	73
GSiW11-Nf-3%	0.226	0.189	1.19	53
8FSiW11-Nf-3%	0.294	0.189	1.55	54
CsPW/Nafion	0.058	0.036	1.61	74
CsPMo/Nafion	0.052	0.036	1.44	74
RN-1-3	0.068	0.056	1.22	75
BPN-Nafion	0.120	0.100	1.20	76
Nafion/P−COF-4	0.141	0.098	1.44	77
Nafion-(NKFs@NSP/PWA)-15 wt%	0.079	0.068	1.16	78
**SiW-POF2(0.3)-S(60%@Nafion-3%**	**0.191**	**0.104**	**1.84**	**This work**

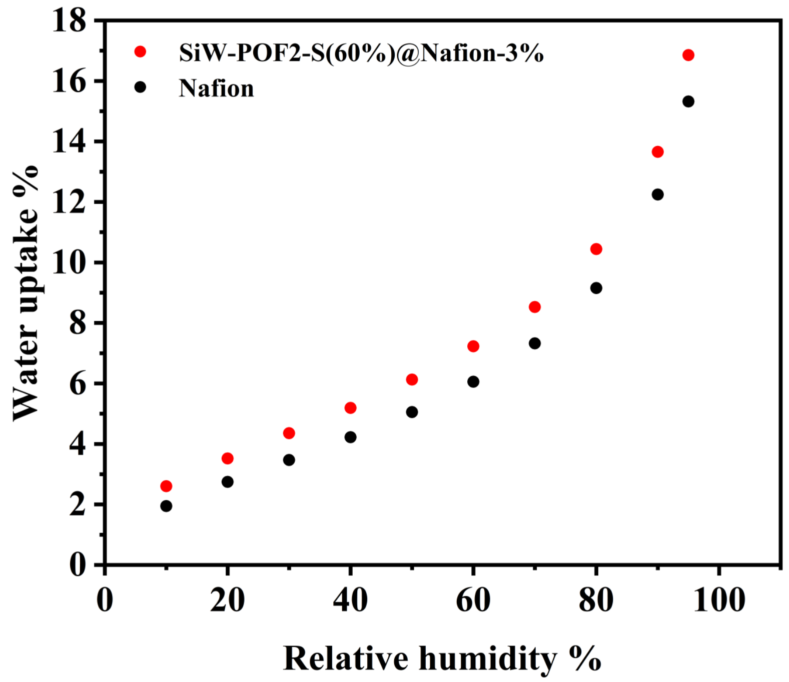

Supplementary Fig. 118: Water uptake versus RH for Nafion and hybrid SiW-POF2-S(60%)@Nafion-3% membranes.

Supplementary Note 6. Stability test for Nafion and hybrid Nafion membranes.

To assess the chemical stability and durability of Nafion and hybrid Nafion membranes, they are fabricated based on the method described in section S10, including pristine Nafion, {SiW_{12}}@Nafion-3% and SiW-POF2-S(60%)@Nafion-3%. Then they are immersed in water at 85 °C for proton conduction durability test (7 days), a variety of aqueous solution with pH ranging from 0-14 and Fenton's reagent to study chemical resistance to acid/base and oxidative degradation (5 days). The membranes after acid/base treatment were characterized by IR spectroscopy and surface SEM, while the samples subjected to Fenton test were evaluated by the weight loss before and after the test.

Preparation of Fenton reagent: In 100 mL water, $FeSO_4 \cdot 7H_2O$ (13.9g, 0.05 mol) and 30 wt% H_2O_2 (20.4 mL) were mixed to afford a homogeneous solution at room temperature.

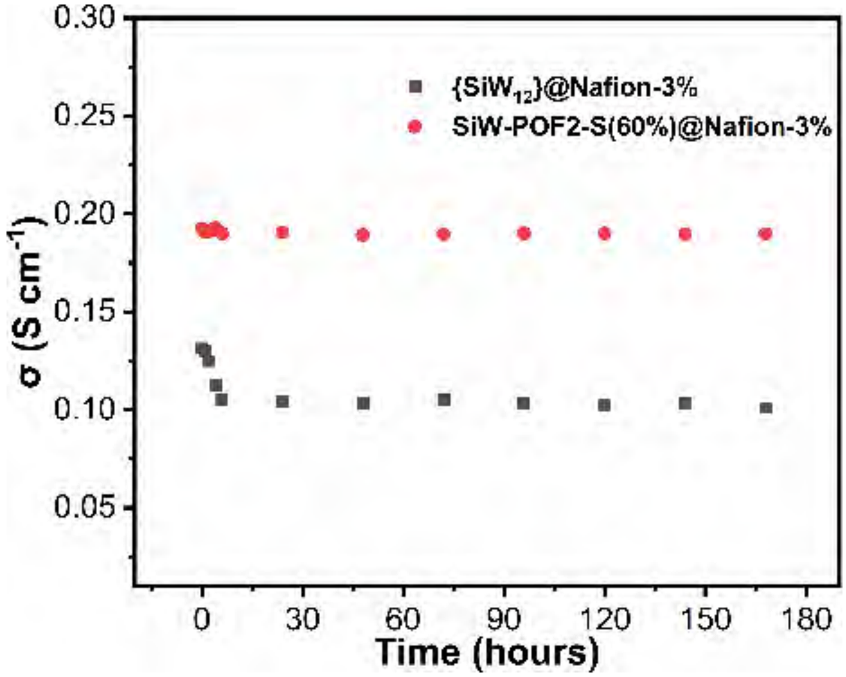

Supplementary Fig. 119: Long-term conducting stability of {SiW_{12}}@Nafion-3% and SiW-POF2-S(60%)@Nafion-3% in water at 85 °C for 168 h.

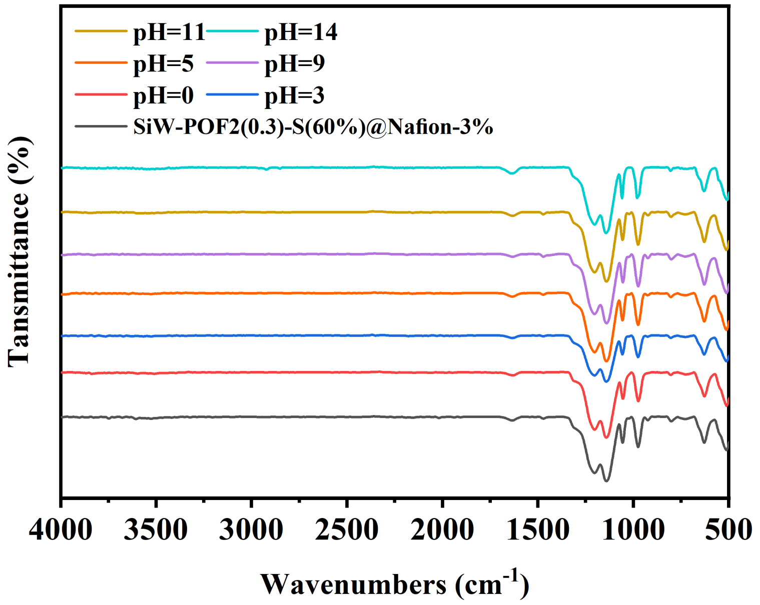

Supplementary Fig. 120: The FTIR spectra of SiW-POF2(0.3)-S(60%)@Nafion-3% membranes before and after immersing in the aqueous solution with pH ranging from 0 to 14 for five days.

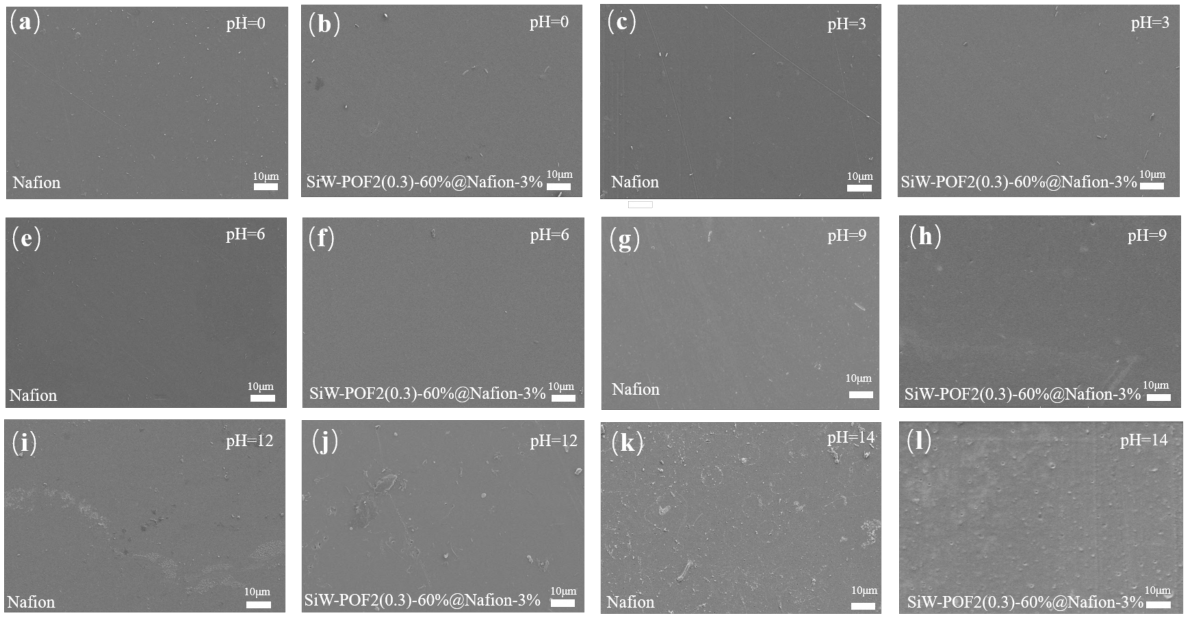

Supplementary Fig. 121: The surface TEM imaging of Nafion and SiW-POF2(0.3)-S(60%)@Nafion-3% membranes after immersing in aqueous solution with pH ranging from 0 to 14 for five days.

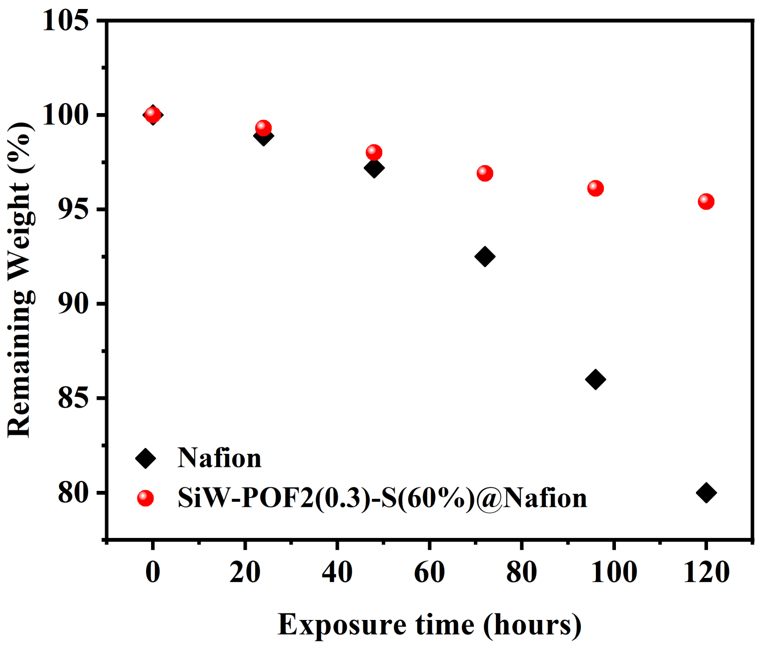

Supplementary Fig. 122: The weight loss of Nafion and SiW-POF2(0.3)-S(60%)@Nafion-3% membranes versus time when exposed to Fenton's reagent.

9. Fuel cell details

The catalyst on the anode side is 0.08 mg cm^{-2} Pt/C, and the catalyst on the cathode side is 0.1 mg cm^{-2} Pt/C. It is sprayed on carbon paper (SGL 39BB), and the membrane is Nafion HP membrane. Between the electrode and the gas diffusion layer, the flow field is set to an active area of 5.0 cm^2. The fuel cell was tested on the Scribner 850e fuel cell system at 85-95 °C and 100 % RH. The back pressure of H_2 and O_2 is 50 kPa. The flow rate of H_2 and O_2 was 0.3/0.3 L min^{-1}.

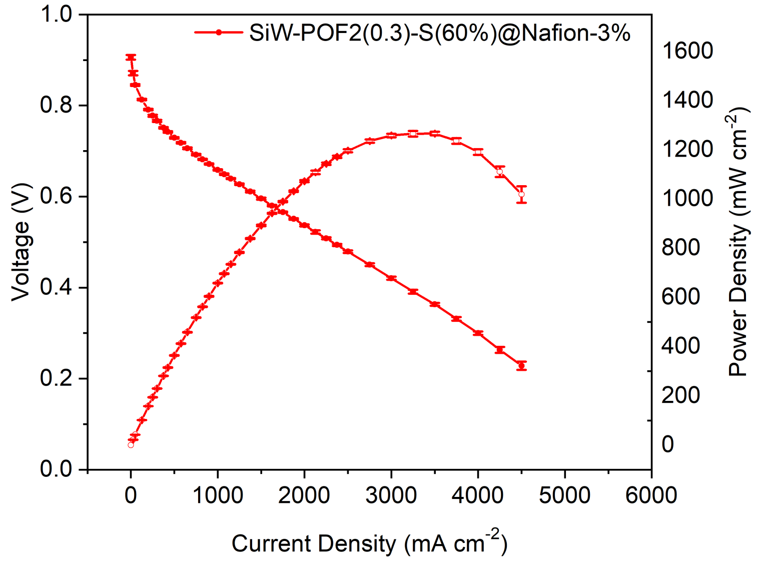

Supplementary Fig. 123: Performance of SiW-POF2(0.3)-S(60%)@Nafion-3% membrane for H_2-O_2 fuel cell at 95 °C and 100% RH. Flow rates of H_2 and O_2 are 0.3/0.3 L min^{-1}. The data are presented as the mean ± s.d. (n = 3).

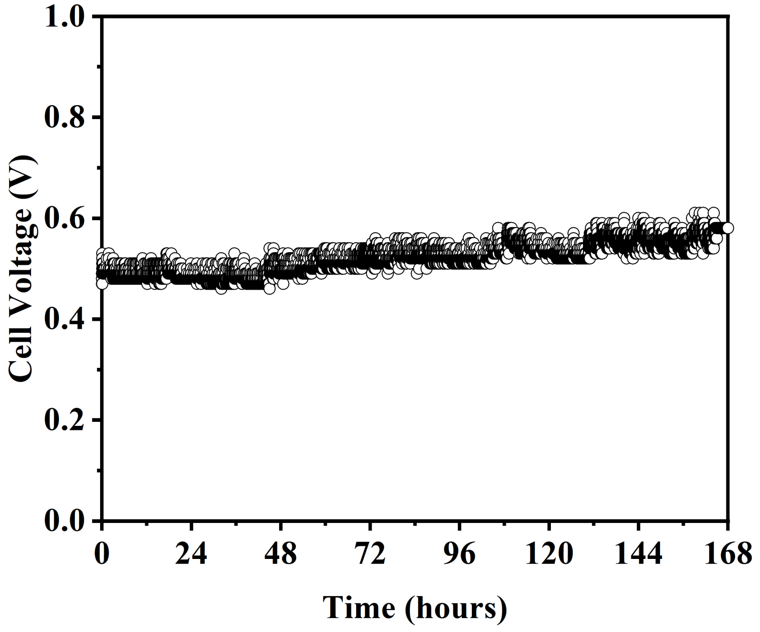

Supplementary Fig. 124: Long-term durability test of the SiW-POF2(0.3)-S(60%)@Nafion membrane at current density of 100 mA cm^{-2} over 168 h. Operating conditions: 90 °C, 100% RH, flow rates of H_2 and O_2 are 0.3/0.3 L min^{-1}.

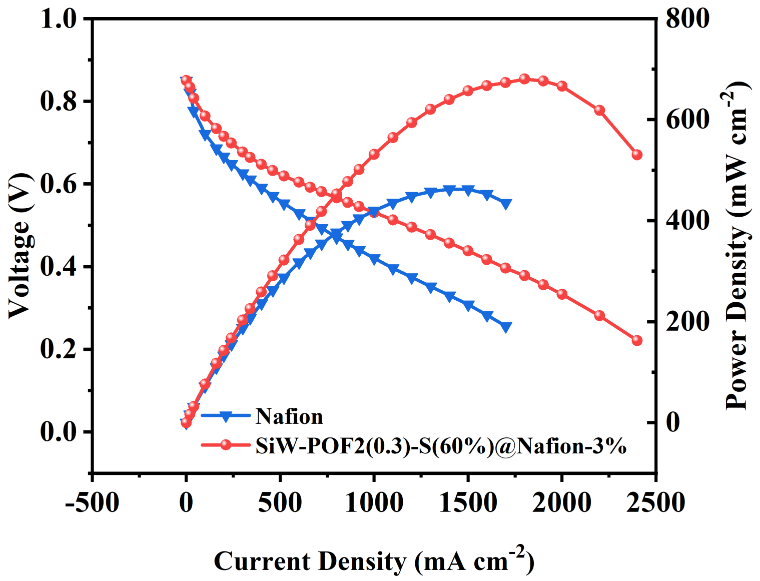

Supplementary Fig. 125: Performance of SiW-POF2(0.3)-S(60%)@Nafion-3% membrane for H_2-O_2 fuel cell at 85 °C and 40% RH. Flow rates of H_2 and O_2 are 0.3/0.3 L min^{-1}.

10. Computational methods

Monte Carlo simulations

Initial host–guest configurations were generated using Monte Carlo (MC) simulations in a rigid SiW-POF2(0.3)-S(60%) framework. The experimental crystal structure was first cleaned to remove disorder and overlapping atoms using Materials Studio,[1] followed by geometry optimisation with the Forcite module, while keeping all framework motion groups fixed, performed using the SMART algorithm in Forcite, a cascade of the steepest descent, adjusted basis set Netwon-Raphson, and quasi-Newton methods. For the geometry optimization, bonded and short-range non-bonded (van der Waals) interactions within the framework were described using the Universal Force Field (UFF)[2]. After geometry optimization, partial atomic charges were calculated and assigned using the PACMAN method[9] and kept fixed throughout all subsequent MC and molecular dynamics (MD) simulations.

Two hydration regimes were considered, with each hydration regime simulated independently in triplicates: i) thermodynamic equilibrium hydration, where water molecules were exchanged with a reservoir under the grand canonical Monte Carlo (GCMC) ensemble at 358.15 K and 94% RH, and ii) high hydration, in which the total number of guest molecules was fixed and sampled using canonical Monte Carlo ensamble (NVT) to generate representative

configuration; the number of water molecules was guided by the experimental water uptake at 94% RH, corresponding to ~ 240 H_2O / unit cell, but, since the water isotherm is of Type I + IV, we did not assume full pore filling and instead used 110 H_2O / unit cell as a conservative estimate of high-hydration loading.

To model proton defects under hydrated conditions, H_3O^+ ions were explicitly included. The number of H_3O^+ ions was chosen such that each excess proton was solvated by approximately ten water molecules ($\lambda = N(H_2O)/N(H_3O^+) \approx 10$). This choice falls within the range commonly used in simulation studies of hydrated proton environments, where extended hydrogen-bonded water networks conducive to proton transport form at similar hydration levels in aqueous and confined systems.[10,11] These excess protons were treated as arising from partial dissociation of sulfonic acid groups under humid conditions. To maintain overall charge neutrality under periodic boundary conditions, an equivalent negative charge was assigned to the corresponding sulfonate groups.

GCMC simulations were performed in a periodic 1 × 1 × 2 supercell of the optimized framework using RASPA. The supercell dimensions were chosen to be at least twice the Lennard-Jones cutoff to avoid spurious self-interactions. The framework was kept rigid throughout. Each GCMC run consisted of 100,000 Monte Carlo cycles, with the first 50,000 cycles treated as equilibration, and the remaining 50,000 as the production run. In each cycle, up to '*N*' trial moves were attempted, where *N* was the current number of guest molecules or 20, whichever was smaller. For equilibrium hydration, trial moves included insertion, deletion, translation and rotation of guest molecules, each selected with equal probability. For the canonical MC, high hydration, only translation and rotation moves were attempted, each with equal probability. In both cases, the number of H_3O^+ ions was fixed, allowing only translation and rotation. Non-bonded interactions were described using Lennard-Jones and Coulomb terms. Lennard-Jones interactions used a 12.8 Å cutoff with Lorentz-Berthelot mixing rules. Long-range electrostatics were treated with Ewald summation with a real-space convergence criterion of 10^{-6}. Framework atoms were assigned LJ parameters from the Dreiding force field and, where unavailable, UFF. Water was described by the SPC/E model,[12] and hydronium employed non-polarizable ion models parametrized for compatibility with SPC/E water.[13] Full force-field parameters and charges are provided in Supplementary Tables S26 – S27. From each GCMC simulation, one representative equilibrated configuration of the 1 × 1 × 2 cell was selected for subsequent MD simulations.

Molecular dynamics simulations

All-atom classical MD simulations were carried out with LAMMPS under periodic boundary conditions.[14] Each 1 × 1 × 2 host-guest configuration obtained from GCMC was replicated 2 × 2 × 2, to generate a 2 × 2 × 4 supercell containing more than 36,000 atoms, including > 1200 H_2O (depending on hydration regime) and 160 H_3O^+ molecules. The resulting triclinic supercell had approximate dimensions of 92.5 × 92.5 × 53.5 Å with a non-orthogonal tilt; the shortest box length of ~ 53.5 Å exceeded 4 times the non-bonded real-space cutoff (Extended Data Figure 3) ensuring proper treatment of long-range interactions. The framework atoms were treated as rigid by zeroing their forces and velocities, so that only the mobile fluid (H_2O and H_3O^+) was thermostatted and propagated. This approximation was used as our primary interest is in the structure and dynamics of the confined fluids. The same force-field parameters used in GCMC were employed in MD. Non-bonded interactions were described using LJ and Coulomb potentials with a 12.0 Å real-space cutoff and Lorentz–Berthelot mixing rules. Long-range electrostatics were treated using a particle–particle particle–mesh (PPPM) Ewald solver with a target accuracy of 10^{-5}. To improve efficiency in the presence of a rigid framework, short-range framework–framework pair interactions were excluded; while all framework-guest and guest-guest interactions were retained. Bonded interactions for water and hydronium (O-H bonds and H-O-H type angles) were represented by harmonic terms and constrained using the SHAKE algorithm, enabling a 1.0 fs timestep in production simulations.

To efficiently relax potential steric overlaps post Monte Carlo insertion into a dense, charged host, we employed a soft 'push-off' protocol before production dynamics.[15] Here, Coulomb interactions and SHAKE constraints were temporarily disabled and the system evolved with a LJ-only pair potential, starting from 5 K. The Lennard-Jones well depth was smoothly ramped from a reduced value to its full magnitude using a time-dependent scaling factor, combined with a small timestep (0.02 – 0.10 fs), under an nve/limit integrator with a Langevin thermostat. After the push-off stage, the full Lennard-Jones + Coulomb potential and PPPM electrostatics were reinstated and SHAKE constraints were re-applied. The system was then gradually heated from 150 K to 358.15 K under an NVT ensemble using a Nosé–Hoover thermostat with a damping time of 100 fs and a timestep of 0.25 – 0.5 fs. The production trajectories were collected in the NVT ensemble at 358.15 K with a 1.0 fs timestep. For each independent starting configuration, we performed 5 ns of production dynamics, discarding the initial equilibration period and using the remaining trajectory for analysis.

We verified that key structural observables (radial distribution functions, hydrogen-bond statistics) and dynamical observables (correlation functions) are converged and do not depend on the specific time window used for averaging; details and correlation-time analyses are provided in Supplementary Note 7. Global virial pressure and total potential energy were not used as observables because rigid host and fixed cell dimensions preclude a direct correspondence to macroscopic pressure.[16]

Supplementary Table 26: Force field parameters for framework atoms used in GCMC and MD simulations.

Atom	ε/k_B(K)	σ (Å)
C[1]	47.856	3.470
N[1]	38.949	3.263
O[1]	48.158	3.033
H[1]	7.649	2.846
Si[1]	155.998	3.804
S[1]	173.107	3.590
W[2]	33.701	3.069

[1]: Dreiding Force Field; [2]: Universal Force Field

Supplementary Table 27: Force field parameters and charges for the guest molecules.

H_2O

Atom	ε/k_B(K)	σ (Å)	Charge
O_water	78.141	3.166	-0.8476
H_water	None	None	0.4238

H_3O^+

Atom	ε/k_B(K)	σ (Å)	Charge
O_hydronium	96.176	3.100	-1.4
H_hydronium	0	0	0.8

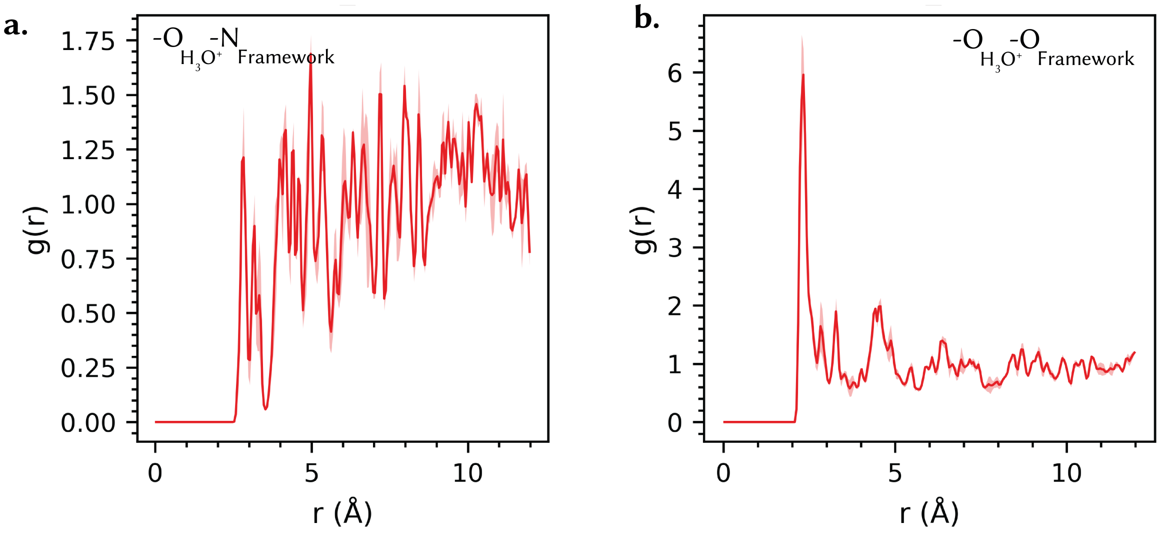

Supplementary Fig. 126: a. RDF of O(H_3O^+) – N(Framework). **b.** RDF of O(H_3O^+) – O(Framework).

Supplementary Note 7. Autocorrelation functions

To quantify relaxation across different dynamical modes and assess equilibration, we computed both velocity autocorrelation functions (VACFs) and orientational autocorrelation functions (OACFs) for H_2O and H_3O^+ in the confined channels (**Supplementary Fig. 127**).[17,18]

The VACF,

$$C_v(t) = \frac{< v(0) \cdot v(t) >}{< v(0) \cdot v(0) >},$$

examines local momentum relaxation. For both H_2O and H_3O^+, the VACFs decay on ultrafast fs timescales, with characteristic relaxation times of $\boldsymbol{\tau_v}$ ~ 0.02 ps (H_2O) and $\boldsymbol{\tau_v}$ ~ 0.009 ps (H_3O^+). This confirms efficient decorrelation of kinetic degrees of freedom and indicates that momentum relaxation is not rate-limiting for longer-time dynamics. Slower collective dynamics associated with hydrogen-bond network reorganization were examined using orientational autocorrelation functions,

$$C_l(t) =< P_l\big(u(0) \cdot u(t)\big) >$$

where $u(t)$ is the instantaneous molecular dipole orientation and P_l is the Legendre polynomial of order l. We focus on the second-rank function $C_2(t)$, which is generally used to characterize rotational relaxation and is invariant to the inversion of the dipole. Both H_2O and H_3O^+ show rapid initial decay within sub-picosecond timescales, corresponding to librational motion, followed by a much slower decay toward a non-zero long-time plateau. The plateau reflects persistent orientational correlations imposed by confinement within the rigid framework – which is generally observed for confined fluids. Convergence to a steady orientational plateau occurs within approximately 1–2 ns. The VACF and OACF analyses yield a clear hierarchy of relaxation times, from ultrafast momentum decorrelation to nanosecond-scale collective orientational relaxation.

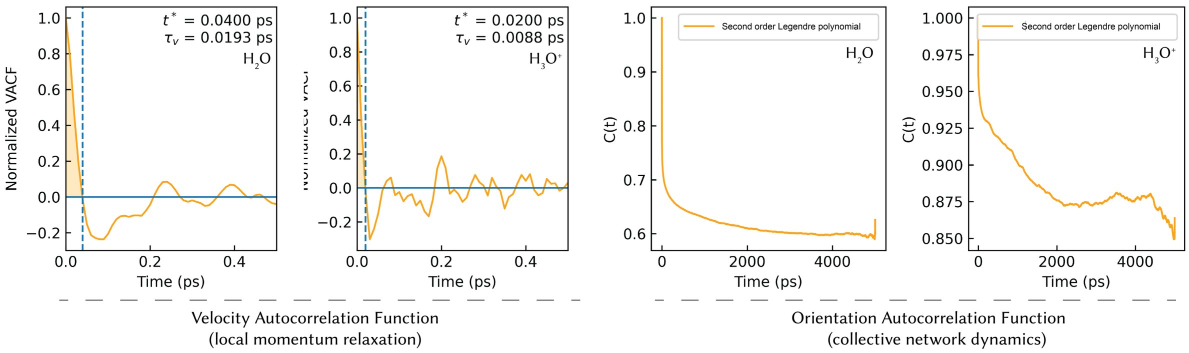

Supplementary Fig. 127. Autocorrelation analysis. Left panels: normalized velocity autocorrelation functions (VACFs) for H_2O and H_3O^+ showing ultrafast momentum relaxation with characteristic times of ~ 0.02 ps (H_2O) and 0.009 ps (H_3O^+). Right panels: orientational autocorrelation functions (OACFs) probing collective reorientation of network dynamics. Both species exhibit rapid librational decay followed by convergence to a long-time plateau – reflecting restricted orientational motion under confinement.

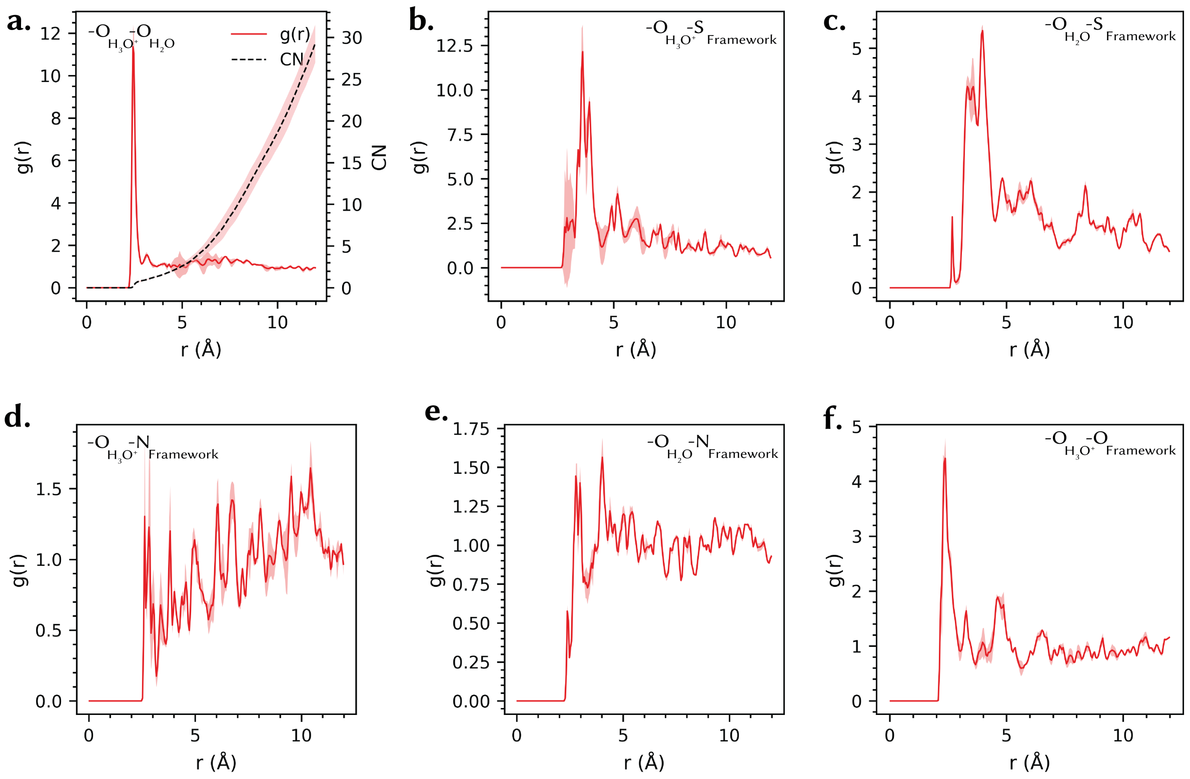

Supplementary Fig. 128. Mechanistic studies in the high-hydration regime. **a.** RDF of O(H_3O^+) – O(H_2O). **b.** RDF of O(H_3O^+) – S(Framework). **c.** RDF of O(H_2O) – S(Framework). **d.** RDF of O(H_3O^+) – N(Framework). **e.** RDF of O(H_2O) – N(Framework). **d.** RDF of O(H_3O^+) – O(Framework).

Supplementary Note 8. On the lower activation energy of the hybrid membranes

We attribute the lower E_a of hybrid membranes to four synergistic factors:

1. The introduction of SiW-POF2(0.3)-S(60%) filler facilitates the fusion of ionic nanophases to generate a more extensive and interconnected network within Nafion matrix.
2. The crystalline SiW-POF2(0.3)-S(60%) nanoparticles provide additional proton-conducting sites and pathways to reinforce proton transport.
3. The surface alkyl sulfonic acids on SiW-POF2(0.3)-S(60%) nanoparticles behave like "cross-linkers" that bridge the ionic domains of Nafion, constructing a more continuous and percolated proton transport pathway.
4. The improved water retention capacity enables a more extensive water hydrogen-bonded network for proton transfer.

In summary, the synergistic interplay between SiW-POF2(0.3)-S(60%) nanoparticles and Nafion matrix enables the proton channels in both materials to become well-percolated. Combined with improved water retention, this architecture creates a more efficient pathway for proton transport, leading to a E_a lower than both the pristine Nafion and SiW-POF2(0.3)-S(60%).

11. References

1. BIOVIA, Dassault Systèmes, Materials Studio, 7.0, San Diego: Dassault Systèmes. (2013).

2. Rappe AK, Casewit CJ, Colwell KS, Goddard WA, III, Skiff WM. UFF, a full periodic table force field for molecular mechanics and molecular dynamics simulations. Journal of the American Chemical Society **114**, 10024-10035 (1992).

3. Dubbeldam D, Calero S, Vlugt TJH. iRASPA: GPU-accelerated visualization software for materials scientists. Molecular Simulation **44**, 653-676 (2018).

4. Sheldrick GM. Crystal structure refinement withSHELXL. Acta Crystallographica Section C Structural Chemistry **71**, 3-8 (2015).

5. Sheldrick GM. SHELXT– Integrated space-group and crystal-structure determination. Acta Crystallographica Section A Foundations and Advances **71**, 3-8 (2015).

6. Dolomanov OV, Bourhis LJ, Gildea RJ, Howard JAK, Puschmann H. OLEX2: a complete structure solution, refinement and analysis program. Journal of Applied Crystallography **42**, 339-341 (2009).

7. Rees B, Jenner L, Yusupov M. Bulk-solvent correction in large macromolecular structures. Acta Crystallographica Section D Biological Crystallography **61**, 1299-1301 (2005).

8. Kohmoto S, et al. Crystal structure of zwitterionic bisimidazolium sulfonates. Journal of Molecular Structure **1015**, 6-11 (2012).

9. Zhao G, Chung YG. PACMAN: A Robust Partial Atomic Charge Predicter for Nanoporous Materials Based on Crystal Graph Convolution Networks. Journal of Chemical Theory and Computation **20**, 5368-5380 (2024)

10. Tripathy M, Sunil Kumar PB, Deshpande AP. Molecular Structuring and Percolation Transition in Hydrated Sulfonated Poly(ether ether ketone) Membranes. The Journal of Physical Chemistry B **121**, 4873-4884 (2017).

11. Sengupta S, Lyulin AV. Molecular Modeling of Structure and Dynamics of Nafion Protonation States. The Journal of Physical Chemistry B **123**, 6882-6891 (2019).

12. Mark P, Nilsson L. Structure and Dynamics of the TIP3P, SPC, and SPC/E Water Models at 298 K. The Journal of Physical Chemistry A **105**, 9954-9960 (2001).

13. Bonthuis DJ, Mamatkulov SI, Netz RR. Optimization of classical nonpolarizable force fields for OH^{-} and H_3O^{+}. Journal of Chemical Physics **144**, 104503 (2016).

14. Gartner TE, Jayaraman A. Modeling and Simulations of Polymers: A Roadmap. Macromolecules 52, 755-786 (2019).

15. Thompson AP, et al. LAMMPS - a flexible simulation tool for particle-based materials modeling at the atomic, meso, and continuum scales. Computer Physics Communications **271**, 108171 (2022).

16. Kapil V, et al. The first-principles phase diagram of monolayer nanoconfined water. Nature **609**, 512-516 (2022).

17. Tan H-S, Piletic IR, Fayer MD. Orientational dynamics of water confined on a nanometer length scale in reverse micelles. Journal of Chemical Physics **122**, 174501 (2005).

18. Balucani U, Brodholt JP, Vallauri R. Analysis of the velocity autocorrelation function of water. J. Phys.: Condens. Matter **8,** 6139-6144 (1996).

19. Li X-X, et al. Ce-mediated molecular tailoring on gigantic polyoxometalate $\{Mo_{132}\}$ into half-closed $\{Ce_{11}Mo_{96}\}$ for high proton conduction. Nature Communications **14**, 5025 (2023).

20. Zhu M, et al. Macrocyclic Polyoxometalates: Selective Polyanion Binding and Ultrahigh Proton Conduction. Angewandte Chemie International Edition **61**, e202200666 (2022).

21. Lin J, et al. Self-Assembly of Giant Mo_{240} Hollow Opening Dodecahedra. Journal of the American Chemical Society **142**, 13982-13988 (2020).

22. Yang P, Alsufyani M, Emwas A-H, Chen C, Khashab NM. Lewis Acid Guests in a $\{P_8W_{48}\}$ Archetypal Polyoxotungstate Host: Enhanced Proton Conductivity via Metal-Oxo Cluster within Cluster Assemblies. Angewandte Chemie International Edition **57**, 13046-13051 (2018).

23. Liu J-C, Han Q, Chen L-J, Zhao J-W, Streb C, Song Y-F. Aggregation of Giant Cerium‐Bismuth Tungstate Clusters into a 3D Porous Framework with High Proton Conductivity. Angewandte Chemie International Edition **57**, 8416-8420 (2018).

24. Cao X-L, et al. A Well-Established POM-based Single-Crystal Proton-Conducting Model Incorporating Multiple Weak Interactions. Chemistry ‐ A European Journal **24**, 2365-2369 (2018).

25. Ji N-N, Shi Z-Q, Xie X-X, Li G. Polyoxometalate-based hydrogen-bonded organic frameworks as a new class of proton conducting materials. CrystEngComm **22**, 8161-8165 (2020).

26. Liu X, Zhang D, Li L, Sun X, Zhang L, Yuan H. Proton conduction in a new 3-D open-framework vanadoborate with an abundant hydrogen bond system. Dalton Transactions **46**, 9103-9109 (2017).

27. Yu Y, et al. Oxalate-assisted assembly of two polyoxotantalate supramolecular frameworks with proton conduction properties. Chemical Communications **59**, 3735-3738 (2023).

28. Cui Y-M, et al. Swift Proton Release Enhancing Proton Conductivity in Anderson-Polyoxometalates-Based Metal‑Organic Frameworks. ACS Materials Letters **6**, 4255-4261 (2024).

29. Liu W-J, et al. Different Protonic Species Affecting Proton Conductivity in Hollow Spherelike Polyoxometalates. ACS Applied Materials & Interfaces **11**, 7030-7036 (2019).

30. Barnett JL, Wenger JS, Getahun A, Johnstone TC, Oliver SRJ. Silver 4,4′-Vinylenedipyridine Coordination Polymers: Linker Effects on Formation Thermodynamics and Anion Exchange. Inorganic Chemistry **64**, 37-49 (2025).

31. Sadakiyo M, Yamada T, Kitagawa H. Rational Designs for Highly Proton-Conductive Metal−Organic Frameworks. Journal of the American Chemical Society **131**, 9906-9907 (2009).

32. Liu L, et al. Enhanced Intrinsic Proton Conductivity of Metal‑Organic Frameworks by Tuning the Degree of Interpenetration. Crystal Growth & Design **18**, 3724-3728 (2018).

33. Liu S-S, Han Z, Yang J-S, Huang S-Z, Dong X-Y, Zang S-Q. Sulfonic Groups Lined along Channels of Metal‑Organic Frameworks (MOFs) for Super-Proton Conductor. Inorganic Chemistry **59**, 396-402 (2020).

34. Phang WJ, et al. Superprotonic Conductivity of a UiO-66 Framework Functionalized with Sulfonic Acid Groups by Facile Postsynthetic Oxidation. Angewandte Chemie International Edition **54**, 5142-5146 (2015).

35. Lu Y-B, et al. Ultra-Stable Metal‑Organic Framework with Concurrent High Proton Conductivity and Fluorescence Sensing for Nitrobenzene. Chemistry of Materials **33**, 7858-7868 (2021).

36. Sharma A, et al. Superprotonic Conductivity of MOF-808 Achieved by Controlling the Binding Mode of Grafted Sulfamate. Angewandte Chemie International Edition **60**, 14334-14338 (2021).

37. Yang F, et al. A flexible metal‑organic framework with a high density of sulfonic acid sites for proton conduction. Nature Energy 2, 877-883 (2017).

38. Wang S, et al. A robust zirconium amino acid metal-organic framework for proton conduction. Nature Communications 9, **4937** (2018).

39. Liu N, et al. Arrangement of Ordered D‑A Components in a Metal-Organic Framework for Cocatalyst-Free Photocatalytic Hydrogen Evolution with Efficient Proton Conduction. Angewandte Chemie International Edition **64**, e202501141 (2025).

40. Yang Y, et al. Combined Intrinsic and Extrinsic Proton Conduction in Robust Covalent Organic Frameworks for Hydrogen Fuel Cell Applications. Angewandte Chemie International Edition **59**, 3678-3684 (2020).

41. Meng Z, Aykanat A, Mirica KA. Proton Conduction in 2D Aza-Fused Covalent Organic Frameworks. Chemistry of Materials **31**, 819-825 (2019).

42. Ranjeesh KC, et al. Imidazole-Linked Crystalline Two-Dimensional Polymer with Ultrahigh Proton-Conductivity. Journal of the American Chemical Society **141**, 14950-14954 (2019).

43. Wu X, et al. Perfluoroalkyl-Functionalized Covalent Organic Frameworks with Superhydrophobicity for Anhydrous Proton Conduction. Journal of the American Chemical Society **142**, 14357-14364 (2020).

44. Ma H, et al. Cationic Covalent Organic Frameworks: A Simple Platform of Anionic Exchange for Porosity Tuning and Proton Conduction. Journal of the American Chemical Society **138**, 5897-5903 (2016).

45. Tao S, Zhai L, Dinga Wonanke AD, Addicoat MA, Jiang Q, Jiang D. Confining H_3PO_4 network in covalent organic frameworks enables proton super flow. Nature Communications **11**, 1981 (2020).

46. Park CH, Lee CH, Guiver MD, Lee YM. Sulfonated hydrocarbon membranes for medium-temperature and low-humidity proton exchange membrane fuel cells (PEMFCs). Progress in Polymer Science **36**, 1443-1498 (2011).

47. Zhang P, et al. Polydopamine-modified sulfonated polyhedral oligomeric silsesquioxane: An appealing nanofiller to address the trade-off between conductivity and stabilities for proton exchange membrane. Journal of Membrane Science **596**, 117734 (2020).

48. Hu M, Zhang B, Chen J, Xu M, Liu D, Wang L. Cross-linked polymer electrolyte membrane based on a highly branched sulfonated polyimide with improved electrochemical properties for fuel cell applications. International Journal of Energy Research **43**, 8753-8764 (2019).

49. Sun X, et al. Phosphoric acid-loaded covalent triazine framework for enhanced the proton conductivity of the proton exchange membrane. Electrochimica Acta **331**, 135235 (2020).

50. Li Y, et al. Fabrication of Nafion/zwitterion-functionalized covalent organic framework composite membranes with improved proton conductivity. Journal of Membrane Science 568, 1-9 (2018).

51. Wu Y, et al. Crystallizing Self-Standing Covalent Organic Framework Membranes for Ultrafast Proton Transport in Flow Batteries. Angewandte Chemie International Edition **62**, e202313571 (2023).

52. Peng Y, et al. Mechanoassisted Synthesis of Sulfonated Covalent Organic Frameworks with High Intrinsic Proton Conductivity. ACS Applied Materials & Interfaces **8**, 18505-18512 (2016).

53. He H, et al. Supramolecular Anchoring of Polyoxometalate Amphiphiles into Nafion Nanophases for Enhanced Proton Conduction. ACS Nano **16**, 19240-19252 (2022).

54. He H, et al. Supramolecular Modifying Nafion with Fluoroalkyl-Functionalized Polyoxometalate Nanoclusters for High-Selective Proton Conduction. Angewandte Chemie International Edition **63**, e202409006 (2024).

55. Li, Shujun, et al. High proton‐conductivity in covalently linked polyoxometalate‐organoboronic acid‐polymers Angew Chem Int Ed. **60**, 16953-16957 (2021).

56. Zhang, Wan-Yu, et al. Incorporation of polyoxometalate-based acid–base pair into a sulfonated MIL-101 for achieving proton-conduction materials with high proton conductivity and high stability Tungsten. 4, 130-137 (2022).

57. Liu, Wen-Jing, et al. Different protonic species affecting proton conductivity in hollow spherelike polyoxometalatesACS Appl. Mater. Interfaces. **11**, 7030-7036 (2019).

58. Cui, Yu-Ming, et al. Swift proton release enhancing proton conductivity in Anderson-polyoxometalates-based metal–organic frameworks. ACS Mater. Lett. **6**, 4255-4261 (2024).

59. Li, Xiao-Min, et al. Superprotonic conductivity of a functionalized metal–organic framework at ambient conditions. ACS Appl. Mater. Interfaces.**14**, 9264-9271 (2022).

60. Ma, Heping, et al. Cationic covalent organic frameworks: a simple platform of anionic exchange for porosity tuning and proton conduction. J. Am. Chem. Soc. **138**, 5897-5903 (2016).

61. Yang, Hui, et al. Proton-conductive Keggin-type clusters decorated by the complex moieties of Cu (II) 2, 2′-bipyridine-4, 4′-dicarboxylate/diethyl analogues. Inorg. Chem. **58**, 1020-1029 (2019).

62. Zhang, Jing, et al. High‐dimensional Polyoxoniobates Constructed from Lanthanide‐incorporated High‐nuclear $\{[Ln(H_2O)_4]_3[Nb_{24}O_{69}(H_2O)_3]_2\}$ Secondary Building Units. Chem. Asian J. **15**, 1574-1579 (2020).

63. Yang, Lu, et al. A crown-shaped 24-molybdate cluster constructed by organotriphosphonate ligand. Inorg. Chem. **52**, 8285-8287 (2013).

64. Bu, Ke, et al. Proton-Conducting Vanadoborate with New [$V_{10}B_{26}$] Clusters. Cryst. Growth Des. **22**, 1824-1830 (2022).

65. Ji, Ning-Ning, et al. Polyoxometalate-based hydrogen-bonded organic frameworks as a new class of proton conducting materials. CrystEngComm. **22**, 8161-8165 (2020).

66. Kong, Cai-Yi, et al. Syntheses, Structures and Proton Conductivities of Two Complexes Based on Decorated Keggin-Type Clusters: {[M (dmphen)$(DMF)_2(H_2O)]_2[SiW_{12}O_{40}]$}·$6H_2O$ (M= Cu and Zn; dmphen= 4, 7-dimethyl-1, 10-phenanthroline). J. Cluster Sci. **28**, 1407-1420 (2017).

67. Zhou, En - Long, et al. Steam - assisted synthesis of an extra - stable polyoxometalate - encapsulating metal azolate framework: applications in reagent purification and proton conduction. Chem. Eur. J. **21**, 13058-13064 (2015).

68. Wang, Ning-Hao, et al. Conjugated acid-base pairs in Keggin-type polyoxometalate-based metal-organic frameworks enhance proton conduction. Chem. Eng. J. **498**, 155821 (2024).

69. Dey, Chandan, Tanay Kundu, and Rahul Banerjee. Reversible phase transformation in proton conducting Strandberg-type POM based metal organic material. Chem. Commun. **48**, 266-268 (2012).

70. Armakola, Eirini, et al. Phosphonate decomposition-induced polyoxomolybdate Dumbbell-type cluster formation: structural analysis, proton conduction, and catalytic sulfoxide reduction. Inorg. Chem. **58**, 11522-11533 (2019).

71. Jiao, Yan-Qing, et al. Self-assembled arrays of polyoxometalate-based metal–organic nanotubes for proton conduction and magnetism. Chem. Commun. **51**, 11313-11316 (2015).

72. Cao, Gao-Juan, et al. A polyoxometalate–organic supramolecular nanotube with high chemical stability and proton-conducting properties. Chem. Commun. **51**, 2048-2051 (2015).

73. Kim, Y., Ketpang, K., Jaritphun, S., Park, J. S. & Shanmugam, S. A polyoxometalate coupled graphene oxide–nafion composite membrane for fuel cells operating at low relative humidity. J. Mater. Chem. A **3**, 8148–8155 (2015).

74. Amirinejad, M., Madaeni, S. S., Rafiee, E. & Amirinejad, S. Cesium hydrogen salt of heteropolyacids/nafion nanocomposite membranes for proton exchange membrane fuel cells. J. Membr. Sci. **377**, 89–98 (2011).

75. Zhai, L. et al. Ionic-nanophase hybridization of nafion by supramolecular patching for enhanced proton selectivity in redox flow batteries. Nano Lett. **23**, 3887–3896 (2023).

76. Hu, B. et al. Structural effects in polyoxometalate-based supramolecular assemblies for enhanced proton conduction. Angew. Chem. Int. Ed. **64**, e202517958 (2025).

77. Zhai, S. et al. High performance nanocomposite proton exchange membranes based on the nanohybrids formed by chemically bonding phosphotungstic acid with covalent organic frameworks. J. Power Sources **554**, 232332 (2023).

78. Yang, X.-B. et al. A highly proton-/vanadium-selective perfluorosulfonic acid membrane for vanadium redox flow batteries. New J. Chem. **43**, 11374–11381 (2019).
